# Correction: *Prevalence of and risk factors for chronic kidney disease of unknown aetiology in India: secondary data analysis of three population-based crosssectional studies*

**DOI:** 10.1136/bmjopen-2018-023353corr1

**Published:** 2019-03-18

O’Callaghan-Gordo C, Shivashankar R, Anand S, *et al*. Prevalence of and risk factors for chronic kidney disease of unknown aetiology in India: secondary data analysis of three population-based crosssectionalstudies. *BMJ Open* 2019;9:e023353.

After the publication of this article, the authors noticed that the map of India showing the study sites needed correction. The borders of India shown in the map were inaccurate and the authors have therefore decided to withdraw this map from the publication. The article has therefore been republished without the original figure 1. Any readers with an academic interest in the information originally displayed in the figure may contact the authors directly in relation to this information.

