# Supplementary material for: Prevalence of and risk factors for chronic kidney disease of unknown aetiology in India: secondary data analysis of three population-based cross-sectional studies
Source: BMJ Open. 2019 Mar 7;9(3):e023353. doi: 10.1136/bmjopen-2018-023353 (PMC6429742; doi:10.1136/bmjopen-2018-023353)
Supplement: Supplementary data [file bmjopen-2018-023353supp001.pdf]

SUPPLEMENTARY MATERIAL

Content

Table S1. Sociodemographic and anthropometric characteristics of overall study participants (prior to exclusion of population with diabetes, hypertension and proteinuria)

Table S2. Associations between sociodemographic and anthropometric characteristics and estimated glomerular filtration rate (eGFR) and eGFR<60 by sex

Table S3. Multiple regression analysis of sociodemographic and anthropometric characteristics associated with eGFR and eGFR<60 including study participants with proteinuria (but without diabetes or hypertension)

Table S4. Multiple regression analysis of sociodemographic and anthropometric characteristics associated with eGFR and eGFR<60 including fasting plasma glucose, HbA1c and systolic blood pressure

Table S1. Sociodemographic and anthropometric characteristics of overall study participants (prior to exclusion of population with diabetes, hypertension and proteinuria)

**Variable**	**n (%)** [a] **n=24774**	**eGFR categories, n(%)** [b]			
		**mean (SD)**	**≥90**	**90 - 60**	**<60**
**Socio-demographic factors**					
Age (years)					
<39	9007 (36)	112.9 (14.9)	8248 (92)	716 (8)	43 (0)
40 - 49	6924 (28)	101.9 (14.8)	5617 (81)	1215 (18)	92 (1)
50 - 59	4524 (18)	92.9 (15.2)	2997 (66)	1378 (30)	149 (3)
60 - 69	3045 (12)	82.8 (17.1)	1410 (46)	1315 (43)	320 (11)
≥70	1274 (5)	72.0 (17.3)	164 (13)	806 (63)	304 (24)
Sex					
Female	13433 (54)	102.6 (19.5)	10404 (77)	2585 (19)	444 (3)
Male	11341 (46)	97.7 (19.3)	8032 (71)	2845 (25)	464 (4)
Education (number of years)					
0	4794 (19)	97.7 (20.2)	3458 (72)	1075 (22)	261 (5)
5	3194 (13)	101.7 (19.4)	2456 (77)	625 (20)	113 (4)
10	8855 (36)	103.2 (18.9)	6995 (79)	1620 (18)	240 (3)
>10	6358 (26)	100.0 (19.2)	4638 (73)	1538 (24)	182 (3)
Missing data	1573 (6)		889 (57)	572 (36)	112 (7)
Area [d]					
Urban	17732 (72)	102 (19.5)	13577 (77)	3602 (20)	553 (3)
Rural	7042 (28)	96.3 (19.1)	4859 (69)	1828 (26)	355 (5)
Latitude [e]					
North	13570 (55)	98.1 (19.1)	9599 (71)	3439 (25)	532 (4)
South	11204 (45)	103.1 (19.7)	8837 (79)	1991 (18)	376 (3)
**Life-style factors**					
Current smoking					
No	18402 (74)	101.5 (19.6)	13920 (76)	3838 (21)	644 (3)
Yes	6372 (26)	97.1 (19.1)	4516 (71)	1592 (25)	264 (4)
Alcohol consumption ever					
No	19588 (79)	100.9 (19.6)	14671 (75)	4203 (21)	714 (4)
Yes	5186 (21)	98.5 (19.1)	3765 (73)	1227 (24)	194 (4)
Vegetarian					
No	15043 (61)	102.7 (19.7)	11721 (78)	2835 (19)	487 (3)
Yes	9731 (39)	96.8 (18.9)	6715 (69)	2595 (27)	421 (4)
**Biological factors**					
Body mass index (kg/m^2)					
Underweight (≤18.5)	10297 (42)	100.1 (19.6)	7626 (74)	2284 (22)	387 (4)
Normal (>18.5 - ≤25)	2403 (10)	101.58 (20.5)	1838 (76)	471 (20)	94 (4)
Overweight (>25 - ≤30)	7221 (29)	99.9 (18.8)	5309 (74)	1680 (23)	232 (3)
Obese (>30)	3286 (13)	99.3 (19.2)	2392 (73)	766 (23)	128 (4)
Missing data	1567 (6)		1271 (81)	229 (15)	67 (4)
Fat free mass (kg)					
1st tertile (≤37)	7141 (29)	101.9 (20.1)	5481 (77)	1381 (19)	279 (4)
2nd tertile (>37 - <45)	7141 (29)	101.3 (19.1)	5419 (76)	1487 (21)	235 (3)

3rd tertile (≥45)	7141 (29)	98.3 (18.6)	5110 (72)	1797 (25)	234 (3)
Missing data	3351 (14)		2426 (72)	765 (23)	160 (5)

[a] Percentages in columns; [b] percentages in rows; [d] Urban areas include Delhi, Chennai and Sonipat district. Rural areas include Sonipat, Vishakhapatnam and Faridabad districts; [e] North areas include Delhi, Sonipat and Faridabad district. South areas include Chennai and Vishakhapatnam districts.

Table S2. Associations between sociodemographic and anthropometric characteristics and estimated glomerular filtration rate (eGFR) and eGFR<60 by sex

	Men, n=5 434			Women, n=7 066		
**Variable**		**eGFR**	**eGFR<60**		**eGFR**	**eGFR<60**
	**n (%)**	**estimate (95%CI) [a]**	**OR (95% CI) [a]**	**n (%)**	**estimate (95%CI) [a]**	**OR (95% CI) [a]**
Age (years) [b]						
<39	2335 (43)	0.00 (ref)	1.00 (ref)	3786 (54)	0.00 (ref)	1.00 (ref)
40-49	1568 (29)	-9 (-9.97, -8.03)	2.36 (1.2, 4.62)	1908 (27)	-12.52 (-13.29, -11.76)	4.5 (1.95, 10.36)
50-59	843 (16)	-16.84 (-18.03, -15.65)	3.82 (1.91, 7.66)	863 (12)	-21.51 (-22.53, -20.48)	11.78 (5.2, 26.68)
60-69	479 (9)	-25.35 (-26.83, -23.86)	13.07 (6.97, 24.49)	414 (6)	-30.05 (-31.46, -28.64)	32.95 (14.87, 73.02)
≥70	209 (4)	-34.26 (-36.4, -32.12)	31.08 (16.33, 59.17)	95 (1)	-34.78 (-37.6, -31.96)	43.43 (15.93, 118.37)
Education (number of completed years)						
0	823 (15)	0.00 (ref)	1.00 (ref)	1997 (28)	0.00 (ref)	1.00 (ref)
≤5	703 (13)	3.28 (1.82, 4.74)	0.24 (0.13, 0.46)	1006 (14)	0.73 (-0.27, 1.73)	0.81 (0.42, 1.56)
6-≤10	2363 (43)	1.68 (0.51, 2.84)	0.31 (0.20, 0.48)	2454 (35)	0.67 (-0.13, 1.48)	0.43 (0.21, 0.86)
>10	1545 (28)	-1.35 (-2.6, -0.1)	0.27 (0.15, 0.47)	1609 (23)	-2.39 (-3.27, -1.5)	0.76 (0.40, 1.46)
Area [c]	3583 (66)					
Urban	1851 (34)	0.00 (ref)	1.00 (ref)	4911 (70)	0.00 (ref)	1.00 (ref)
Rural		-4.02 (-4.85, -3.19)	2.72 (1.84, 4.01)	2155 (30)	-3.69 (-4.36, -3.02)	1.99 (1.26, 3.14)
Latitude [d]						
North	2861 (53)	0.00 (ref)	1.00 (ref)	3402 (48)	0.00 (ref)	1.00 (ref)
South	2573 (47)	-1.52 (-2.3, -0.74)	1.76 (1.21, 2.56)	3664 (52)	2.58 (1.96, 3.19)	1.30 (0.83, 2.05)
Current tobacco consumption						
No	2804 (52)	0.00 (ref)	1.00 (ref)	6553 (93)	0.00 (ref)	1.00 (ref)
Yes	2630 (48)	1.15 (0.36, 1.93)	1.32 (0.91, 1.92)	513 (7)	-1.93 (-3.14, -0.73)	1.54 (0.87, 2.73)
Alcohol consumption ever						
No	3035 (56)	0.00 (ref)	1.00 (ref)	7059 (100)	0.00 (ref)	1.00 (ref)

Yes	2399 (44)	-0.71 (-1.49, 0.06)	1.57 (1.08, 2.27)	7 (0)	-9.29 (-18.97, 0.4)	1.00 (1.00, 1.00)
Vegetarian						
No	3576 (66)	0.00 (ref)	1.00 (ref)	4396 (62)	0.00 (ref)	1.00 (ref)
Yes	1858 (34)	0.65 (-0.18, 1.48)	0.61 (0.41, 0.90)	2670 (38)	-2.11 (-2.75, -1.47)	0.70 (0.44, 1.11)
Body mass index (kg/m2)						
Underweight (≤18.5)	2888 (56)	0.00 (ref)	1.00 (ref)	2991 (44)	0.00 (ref)	1.00 (ref)
Normal (>18.5 - ≤25)	812 (16)	4.05 (2.92, 5.18)	0.69 (0.42, 1.14)	764 (11)	1.61 (0.57, 2.65)	1.07 (0.57, 2.03)
Overweight (>25 - ≤30)	1209 (23)	-1.7 (-2.68, -0.73)	0.71 (0.42, 1.21)	2104 (31)	-0.11 (-0.84, 0.62)	0.67 (0.38, 1.20)
Obese (>30)	243 (5)	-0.71 (-2.61, 1.18)	0.36 (0.09, 1.50)	907 (13)	-0.64 (-1.61, 0.33)	0.55 (0.23, 1.31)
Fat free mass (kg)						
1st tertile (≤37)	361 (8)	0.00 (ref)	1.00 (ref)	3833 (58)	0.00 (ref)	1.00 (ref)
2nd tertile (>37 - <45)	1351 (28)	-0.42 (-2.10, 1.25)	0.78 (0.44, 1.38)	2535 (39)	-1.39 (-2.04, -0.74)	0.67 (0.38, 1.17)
3rd tertile (≥45)	3093 (64)	-3.75 (-5.35, -2.16)	0.50 (0.28, 0.90)	208 (3)	-1.36 (-3.17, 0.45)	0.58 (0.08, 4.25)

[a] Adjusted for age; [b] Not adjusted for age; [c] Urban areas include Delhi, Chennai and Sonipat district. Rural areas include Sonipat, Vishakhapatnam and Faridabad districts; [d] North areas include Delhi, Sonipat and Faridabad district. South areas include Chennai and Vishakhapatnam districts.

Table S3. Multiple regression analysis of sociodemographic characteristics associated with eGFR and eGFR<60 including study participants with proteinuria (but without diabetes or hypertension), n=12533

	**eGFR**	**eGFR<60**
**Variable**	**Coefficient (95%CI)** [a]	**OR (95%CI)** [a]
Area [b]		
Urban	0.00 (ref)	1.00 (ref)
Rural	-4.59 (-5.14, -4.03)	1.93 (1.40, 2.66)
Latitude [c]		
North	0.00 (ref)	1.00 (ref)
South	0.29 (-0.21, 0.78)	1.33 (0.98, 1.80)
Education (number of years)		
0	0.00 (ref)	1.00 (ref)
5	0.83 (0, 1.66)	0.55 (0.35, 0.87)
10	0.04 (-0.64, 0.72)	0.51 (0.35, 0.76)
>10	-3.81 (-4.58, -3.04)	0.66 (0.40, 1.07)
Alcohol consumption ever		
No	0.00 (ref)	1.00 (ref)
Yes	-0.78 (-1.52, -0.05)	1.23 (0.85, 1.79)
Sex		
Female	0.00 (ref)	1.00 (ref)
Male	-2.86 (-3.46, -2.26)	1.38 (0.96, 1.98)
Age (per 10 years)	-9.12 (-9.34, -8.91)	2.23 (2.00, 2.49)

[a] Variables mutually adjusted, [b] Urban areas include Delhi, Chennai and Sonipat district. Rural areas include Sonipat, Vishakhapatnam and Faridabad districts; [c] North areas include Delhi, Sonipat and Faridabad district. South areas include Chennai and Vishakhapatnam districts.

Table S4. Multiple regression analysis of sociodemographic characteristics associated with eGFR and eGFR<60 including plasma fasting glucose, HbA1c and systolic blood pressure

	**eGFR**	**eGFR<60**
**Variable**	**Coefficient (95%CI)** [a]	**OR (95%CI)** [a]
Area [b]		
Urban	0.00 (ref)	1.00 (ref)
Rural	-4.94 (-5.51, -4.38)	2.29 (1.64, 3.20)
Latitude [c]		
North	0.00 (ref)	1.00 (ref)
South	0.23 (-0.26, 0.72)	1.30 (0.95, 1.77)
Education (number of years)		
0	0.00 (ref)	1.00 (ref)
5	1.03 (0.20, 1.86)	0.49 (0.31, 0.79)
10	0.19 (-0.49, 0.87)	0.47 (0.32, 0.71)
>10	-3.53 (-4.30, -2.76)	0.62 (0.38, 1.02)
Alcohol consumption ever		
No	0.00 (ref)	1.00 (ref)
Yes	-0.72 (-1.46, -0.01)	1.32 (0.90, 1.93)
Sex		
Female	0.00 (ref)	1.00 (ref)
Male	-2.69 (-3.29, -2.09)	1.47 (1.01, 2.12)
Age (per 10 years)	-8.93 (-9.16, -8.70)	2.11 (1.89, 2.38)
Fasting plasma glucose (mg/dl)	-0.06 (-0.08, -0.04)	1.01 (1.00, 1.02)
Hb1Ac (%)	0.03 (-0.56, 0.62)	1.95 (1.34, 2.85)
Systolic blood pressure (mm Hg)	-0.06 (-0.84, -0.04)	1.0 (0.99, 1.02)

[a] Variables mutually adjusted, [b] Urban areas include Delhi, Chennai and Sonipat district. Rural areas include Sonipat, Vishakhapatnam and Faridabad districts; [c] North areas include Delhi, Sonipat and Faridabad district. South areas include Chennai and Vishakhapatnam districts.
