# Supplementary material for: Prevalence of and risk factors for chronic kidney disease of unknown aetiology in India: secondary data analysis of three population-based cross-sectional studies
Source: BMJ Open. 2019 Mar 7;9(3):e023353. doi: 10.1136/bmjopen-2018-023353 (PMC6429742; doi:10.1136/bmjopen-2018-023353)
Supplement: Author's manuscript [file bmjopen-2018-023353.draft_revisions.pdf]

BMJ Open is committed to open peer review. As part of this commitment we make the peer review history of every article we publish publicly available.

When an article is published we post the peer reviewers' comments and the authors' responses online. We also post the versions of the paper that were used during peer review. These are the versions that the peer review comments apply to.

The versions of the paper that follow are the versions that were submitted during the peer review process. They are not the versions of record or the final published versions. They should not be cited or distributed as the published version of this manuscript.

BMJ Open is an open access journal and the full, final, typeset and author-corrected version of record of the manuscript is available on our site with no access controls, subscription charges or pay-per-view fees (http://bmjopen.bmj.com).

If you have any questions on BMJ Open's open peer review process please email editorial.bmjopen@bmj.com

BMJ Open

Prevalence of and risk factors for chronic kidney disease of unknown aetiology in India: secondary data analysis of three population-based cross-sectional studies

Journal:	*BMJ Open*
Manuscript ID	bmjopen-2018-023353
Article Type:	Research
Date Submitted by the Author:	04-Apr-2018

Complete List of Authors:	O'Callaghan Gordo, Cristina; Instituto de Salud Global Barcelona, Campus Mar<br>Shivashankar, Roopa; Public Health Foundation of India,<br>Anand, Shuchi; Stanford Hospital and Clinics,<br>Ghosh, Shreeparna; Public Health Foundation of India,<br>Glaser, Jason; La Isla Foundation; London School of Hygiene and Tropical Medicine, 3Department of Non-communicable Disease Epidemiology<br>Gupta, Ruby; Publichealth Foundation of India<br>Jakobsson , Kristina ; Lunds Universitet Arbets- och miljomedicin<br>Kondal, Dimple; Publichealth Foundation of India<br>Krishnan , Anand ; All India Institute of Medical Sciences Centre for Community Medicine<br>Mohan, Sailesh; Public Health Foundation of India,<br>Mohan, V; Madras Diabetes Research Foundation<br>Nitsch, Dorothea; LSHTM<br>PA , Praveen ; All India Institute of Medical Sciences, Department of Endocrinology and Metabolism<br>Tandon, Nikhil; All India Institute of Medical Sciences,<br>Narayan, K; Emory University School of Public Health, Global Health<br>Pearce, Neil; London School of Hygiene and Tropical Medicine<br>Caplin, Ben; University College London Medical School, Centre for Nephrology,<br>Prabhakaran, Dorairaj; Public Health Foundation of India, Centre for Control of Chronic Conditions and Injuries

Keywords:	EPIDEMIOLOGY, NEPHROLOGY, Chronic renal failure < NEPHROLOGY, PUBLIC HEALTH

SCHOLARONE™
Manuscripts

TITLE PAGE

Title: Prevalence of and risk factors for chronic kidney disease of unknown aetiology in India: secondary data analysis of three population-based cross-sectional studies

Authors names and affiliations:

Cristina O'Callaghan-Gordo[1,2,3,4*]; Roopa Shivashankar[5, 6*]; Shuchi Anand [7]; Shreeparna Ghosh[5]; Jason Glaser[4, 8]; Ruby Gupta[5]; Kristina Jakobsson[9,10]; Dimple Kondal[5, 6]; Anand Krishnan[11]; Sailesh Mohan[5]; Viswanathan Mohan[12, 13]; , Dorothea Nitsch[14]; Praveen PA[6, 15]; Nikhil Tandon[15]; K.M. Venkat Narayan[16]; Neil Pearce[4, 17]; Ben Caplin[18**]; Dorairaj Prabakharan[5, 6**].

[1] ISGlobal, Barcelona, Spain; [2] Universitat Pompeu Fabra (UPF), Barcelona, Spain; [3] CIBER Epidemiología y Salud Pública (CIBERESP), Madrid, Spain; [4] Department of Medical Statistics, London School of Hygiene and Tropical Medicine, London, UK; [5] Public Health Foundation of India (PHFI), New Delhi, India; [6] Centre for Control of Chronic Conditions (4Cs), New Delhi, India; [7] Stanford University School of Medicine; [8] La Isla Network; [9] Occupational and Environmental Medicine, Sahlgrenska Academy, Gothenburg University, Sweden; [10] Occupational and Environmental Medicine, Lund University, Sweden; [11] Centre for Community Medicine, All India Institute of Medical Sciences, New Delhi.; [12] Diabetes Research, Madras Diabetes Research Foundation, Chennai, India; [13] Dr. Mohan's Diabetes Specialities Centre, Chennai, India; [14] Department of Epidemiology and Population Health, London School of Hygiene and Tropical Medicine, , London, UK; [15] Department of Endocrinology and Metabolism, All India Institute of Medical Sciences, New Delhi, India; [16] Emory Global Diabetes Research Center, Rollins School of Public Health, Emory University, Atlanta, GA, USA; [17] Centre for Global NCDs, London School of Hygiene and Tropical Medicine, London, UK; [18] Centre for Nephrology, University College London Medical School, London, UK.

[*] Joint first authors; [**] Joint last authors

Corresponding author's name and email address:

Cristina O'Callaghan-Gordo, **cristina.ocallaghan@isglobal.org**

Word count: 3521

For peer review only - http://bmjopen.bmj.com/site/about/guidelines.xhtml

ABSTRACT

Objectives: To assess whether chronic kidney disease of unknown aetiology (CKDu) is present in India and to identify risk factors for it using population-based data and standardised methods.

Design: Secondary data analysis of three population-based cross-sectional studies conducted between 2010-2014.

Setting: Urban and rural areas of Northern India (states of Delhi and Haryana) and Southern India (states of Tamil Nadu and Andhra Pradesh)

Participants: 12,500 individuals without diabetes, hypertension or heavy proteinuria

Outcome measures: Mean estimated the glomerular filtration rate (eGFR) and the prevalence of eGFR below 60ml/min per 1.73m2 (eGFR<60) in individuals without diabetes, hypertension or heavy proteinuria (proxy definition of CKDu).

Results: The mean eGFR was 105.0±17.8 ml/min per 1.73m2. The prevalence of eGFR<60 was 1.6% (95%CI=1.4-1.7), but this figure varied markedly between areas, being highest in rural areas of Southern Indian [4.8% (3.8-5.8)]. In Northern India, older age was the only risk factor associated with lower mean eGFR and eGFR<60 [regression coefficient (95%CI)=-0.94 (0.97 – 0.91); OR (95%CI)=1.10 (1.08-1.11)]. In Southern India, risk factors for lower mean eGFR and eGFR<60 were residence in a rural area [-7.78 (-8.69 - -6.86); 4.95 (2.61-9.39)], older age [-0.90 (-0.93 - -0.86); 1.06 (1.04-1.08)] and less education [-0.94 (-1.32 - -0.56); 0.67 (0.50-0.90) for each five years at school].

Conclusions: CKDu is present in India and is not confined to Central America and Sri Lanka. Identified risk factors are consistent with risk factors previously reported for CKDu in Central America and Sri Lanka.

KEYWORDS

Epidemiology; Chronic kidney disease; Chronic kidney disease of unknown aetiology;; India; Rural population

ARTICLE SUMMARY

Strengths and limitations of this study

- The use of a random selection of population-based participants allows the estimation of CKDu prevalence in the general population.

For peer review only - http://bmjopen.bmj.com/site/about/guidelines.xhtml

- A large sample size including participants from different areas of India (urban and rural, and Northern and Southern India) increases the representativeness of our results.
- The use of standardized definitions of CKDu facilitates international comparisons of CKDu prevalence and risk factors.
- The prevalence of eGFR<60 observed in this study is likely to be underestimated; however, this is unlikely to have biased the internal comparisons conducted in this study.

FUNDING

This work was supported in part by grant MR/P02386X/1 from the United Kingdom Medical Research Council under the Global Challenges Research Fund. It was also supported by grants from the Colt Foundation and the La Isla Foundation. The CARRS study was funded with federal funds from the National Heart, Lung, and Blood Institute, National Institutes of Health, under Contract No. HHSN2682009900026C. UDAY study was funded by Eli Lilly Foundation. ICMR-CHD study was funded by the Indian Council Medical Research (ICMR). The Centre for Global NCDs is supported by the Wellcome Trust Institutional Strategic Support Fund (097834/Z/11/B). CO-G holds a Sara Borrell postdoctoral fellowship awarded from the Carlos III National Institute of Health, Spain (CD13/00072).

For peer review only - http://bmjopen.bmj.com/site/about/guidelines.xhtml

INTRODUCTION

High prevalence of CKDu has mainly been reported in the last decades amongst the working age populations of agricultural communities of tropical/subtropical regions, specifically in Central America and Sri Lanka (C Wesseling et al. 2013; Correa-Rotter, Wesseling, and Johnson 2014; Jayatilake et al. 2013). In Nicaragua and El Salvador, the estimated prevalence of estimated glomerular filtration rate (eGFR; the clinical measure of kidney function) below 60ml/min per 1.73m^2 (eGFR<60), in the absence of diabetes and hypertension, was 10-20% (Lebov et al. 2015; Peraza et al. 2012; Torres et al. 2010). It has been suggested that CKDu may also be highly prevalent in other low and middle income countries (LMICs), including India (Seck et al. 2014; Barsoum 2013; El Minshawy, Ghabrah, and El Bassuoni 2014; Rajapurkar et al. 2012; Reddy and Gunasekar 2013). However, it is not clear in which other regions of the world CKDu occurs, whether the underlying aetiology is the same in different regions and what the risk factors are. Currently, there is no consensus but factors such as heat stress, strenuous work, climatic conditions, agrochemical use, heavy metal exposure and infections have been suggested as risk factors (Jayasumana et al. 2015; C Wesseling et al. 2013; Catharina Wesseling et al. 2015; Garcia-Garcia, Jha, and World Kidney Day Steering Committee 2015; Robey 2014).

Data on CKDu from India are scarce. The recent report of verbal autopsy data from India suggests CKD of all causes is a growing problem. However, it does not provide accurate population-based data on CKDu (Jha and Modi 2017; Dare et al. 2017). Existing reports indicate that CKDu may be common but it is difficult to be definite about this because of the absence of population-based studies using standardised and comparable methods. Data from the Indian CKD Registry, a hospital based registry of incident cases of CKD between 2006-2010, found that CKDu was the second commonest form of CKD after diabetic nephropathy (Rajapurkar et al. 2012). However, this is restricted to referred cases and therefore may not be representative of the general population. There are also sporadic reports of high numbers of CKDu cases among agricultural communities of the South Eastern Indian states of Andhra Pradesh and Odisha (reviewed by Chatterejee (Chatterjee 1026) and Ganguli (Ganguli 2016)). However, population-based data have not been reported for India.

We conducted a secondary analysis of representative sample surveys conducted in India between 2010-2014. Given the absence of a clear case definition for CKDu it is necessary to make a presumptive diagnosis based on measures/estimates of GFR in the absence of known risk factors for kidney disease. We therefore here report both the distribution eGFR and prevalence of eGFR below 60ml/min per 1.73m^2 (eGFR<60), and the risk factors

For peer review only - http://bmjopen.bmj.com/site/about/guidelines.xhtml

associated with these outcomes, in a population restricted to those without known risk factors for CKD, i.e. diabetes, hypertension or heavy proteinuria (a marker of primary glomerular disease).

METHODS

Study population

We used cross-sectional data from three population-based studies conducted in India: the "Centre for Cardiometabolic Risk Reduction in South Asia" cohort study (CARRS study) (Nair et al. 2012), the "Implementing a Comprehensive Diabetes Prevention and Management Program" study (UDAY study) (Mohan et al. 2017) and the "prevalence of coronary heart disease repeat survey" study funded by the Indian Council of Medical Research (ICMR-CHD study) (Prabhakaran et al. 2017). Details on study design and selection of participants from the CARRS, UDAY and ICMR-CHD studies have been previously described (Nair et al. 2012; Prabhakaran et al. 2017; Mohan et al. 2017) and are summarized in Table 1. Participants from CARRS, UDAY and ICMR-CHD studies provided informed consent prior to participation. The three studies obtained ethical clearance from the corresponding institutions.

For the current analyses, we excluded participants with missing information on serum creatinine, sex and age, as these variables were necessary to estimate eGFR. As the focus of our study was CKDu, we excluded participants with known risk factors for CKD (i.e. diabetes and hypertension) or evidence of primary glomerular disease (as assessed by heavy proteinuria) or with missing information for these risk factors. We also excluded participants with missing information on basic co-variables (education) for all the analyses conducted. A study flowchart is presented in Figure 1. We classified participants as having: diabetes, if plasma fasting glucose was ≥126 mg/dl, or glycated haemoglobin A1c (HbA1c) was ≥6.5%, or the participant self-reported diabetes and was on oral hypoglycaemic medication; hypertension, if systolic blood pressure was ≥140 mm Hg, or diastolic blood pressure was ≥90 mm Hg, or the participant self-reported hypertension and was on antihypertensive medication; and heavy proteinuria, if the albumin/creatinine ratio (ACR) in urine was ≥300 mg/g. We used the CKD-EPI equation to estimate GFR (eGFR) (Levey et al. 2009).

Data collection and laboratory analyses

Data collection was conducted between October 2010 and December 2014. All three studies used a standardized questionnaire to collect data on age, sex, completed years of education (0, ≤5, >5-≤ 10, >10), alcohol intake

For peer review only - http://bmjopen.bmj.com/site/about/guidelines.xhtml

(ever, never) and dietary habits (vegetarian yes, no). Body mass index (BMI, kg/m^2) was calculated and categorized (≤18.5: underweight; >18.5-≤25: normal weight; >25-≤30: overweight; >30: obese), fat free mass was derived from bioelectric impedance analysis (BIA) and blood pressure was measured using an electronic sphygmomanometer, as previously reported (Nair et al. 2012; Anand et al. 2015). A fasting venous blood sample was used to measure glucose levels, HbA1c and serum creatinine levels and urine sample to measure albuminuria and creatinuria (Nair et al. 2012). Glucose levels were measured using hexokinase/kinetic methods, HbA1c using high-performance liquid chromatography, and the serum creatinine using the rate-blanked and compensated kinetic Jaffe method, traceable to isotope dilution mass spectrometry (Nair et al. 2012).

Statistical analyses

We reported mean eGFR and prevalence eGFR<60 according to different characteristics of the study populations. UDAY and CARRS studies did not involve fully random population samples (since sampling was based on households, with one participant per household) and the proportions of study participants with particular outcomes (e.g. eGFR<60), will not be exactly the same (but very similar) to what would have been obtained with genuine random population samples; thus in this paper we refer to the prevalence in the study participants, not overall population prevalence estimates. We used linear regression models to estimate the associations between potential risk factors and eGFR and logistic regression models to estimate the associations between potential risk factors and eGFR<60. We also repeated the analyses separately for males and females. Variables associated with eGFR in the basic analyses (adjusted for age and sex) were considered for the multiple regression analysis. In the final multiple regression model, we included all variables that were of a priori interest and/or had shown independent associations with eGFR. We then checked for multicollinearity for each variable in the multiple regression analyses in comparison with the basic analyses (Greenland et al. 2016). 6% of participants had missing values for education, 4% for BMI and 11% for fat free mass. For BMI and fat free mass, we excluded participants with missing values to compare models non-adjusted and adjusted for these variables. We calculated prevalence ratios of eGFR<60 by age-group for rural and urban population. Finally, we estimated potential interactions between urban (versus rural) residence and latitude (Northern India (i.e. states of Delhi and Haryana) versus Southern India (states of Tamil Nadu and Andhra Pradesh)). We conducted all analyses using Stata version 14 (StataCorp, College Station, TX, USA).

Patient and Public Involvement

For peer review only - http://bmjopen.bmj.com/site/about/guidelines.xhtml

Patients were not involved in the design of this analysis.

RESULTS

Characteristics of study participants

12,500 people were eligible for the current analyses (Figure 1). Table 2 summarizes the socio-demographic and anthropometric characteristics of the 12,500 study participants included in this analysis (the same information including participants with known risk factors for CKD (n=24,774) in supplementary material Table S1). The mean (standard deviation (±SD)) age of participants was 41.5±11.6 years. 88% (4,805/5,434) of the male population was formally employed; 76% (5,346/7,066) of women worked on house duties (i.e. housewives). The mean BMI was 24±5.0 kg/m^2 and mean fat free mass was 42±15 kg/m^2. The mean fasting plasma glucose was 91.9±12.3 mg/dl and the mean HbA1c was 5.5±0.4 %. The mean systolic and diastolic blood pressures were 114±12 mm Hg and 74±9 mm Hg, respectively. The median (inter quartile range, IQR) albumin/creatinine ratio (ACR) was 2.4 (4.3) mg/g (after exclusion of those with ACR>300mg/g, n=1,208).

Mean eGFR and prevalence of eGFR<60

The mean eGFR was 105.0±17.8 ml/min per 1.73m^2. The mean eGFR was lower at increasing ages, in males, in inhabitants from rural areas and in those from Northern India, in participants with no formal education, and in participants who reported tobacco consumption, alcohol intake and being vegetarian (Table 2). We observed differences in mean eGFR depending on the area, being 104.5±17.6 in urban areas of Northern India, 100.3±16.2 in rural areas of Northern India, 110.9±15.7 in urban areas of Southern India and 97.4±19.8 in the rural area of Southern India.

The prevalence of eGFR<60 among the study population was 1.6% (95% confidence interval (95% CI)=1.4% - 1.9%). 17% (95% CI=16% - 17%) of study participants had eGFR≥60-<90 ml/min per 1.73m^2 and 82% [95% confidence interval (95% CI)=81% – 82%] had eGFR≥90 ml/min per 1.73m^2. The prevalences of different categories of eGFR differed by formal education, tobacco consumption, alcohol intake and vegetarianism (Table 2). Also, we observed marked differences in the prevalence of eGFR<60 depending on the area, being 1.4 % (95% CI=1.1 – 1.8) in urban areas of Northern India, 1.9 (95% CI=1.4% – 2.6%) in rural areas of Northern India, 0.43% (95% CI =0.03% – 0.07%) in urban areas of Southern India and 4.8 % (95% CI= 3.9% – 5.9%) in

For peer review only - http://bmjopen.bmj.com/site/about/guidelines.xhtml

the rural area of Southern India. The prevalence ratio of eGFR<60 for rural versus urban residence was higher for participants <50 years than for older groups (Figure 2).

Risk factors for lower eGFR and eGFR<60

As expected, age was an important risk factor for reduced eGFR: eGFR was 0.93 ml/min per 1.73 m^2 (95%CI=-0.95 - -0.91, model adjusted for sex) lower for each additional year of age. Additionally, being male, living in a rural setting, living in Southern India and consuming alcohol were associated with decreased mean eGFR (Table 3). Similarly, the odds of eGFR<60 also increased by each year of age [OR adjusted for sex (95%CI)=1.1 (1.1 - 1.1)] and being male, living in a rural setting, living in Southern India and consuming alcohol were also associated with eGFR<60 (Table 3). Risk factors for decreased mean eGFR and for eGFR<60 were similar for men and women (supplementary material, Table S2).

In the multiple regression analyses, decreased mean eGFR remained associated with older age, being male and living in a rural setting and alcohol consumption (Table 4). Risk of eGFR<60 remained associated with older age, being male and living in a rural setting and having no formal education remained associated with increased risk of eGFR<60 (Table 4). We adjusted all the multiple regression models for fat free mass and vegetarianism to assess the possibility that differences observed between urban and rural participants were due to differences in diet and/or body composition. These adjustments had little effect on the results (Table 4).

We observed an interaction between the effects of latitude (North/South) and urban/rural residence in association with reduced eGFR (p-value for interaction<0.001). The mean eGFR was lower in rural settings in both Northern and Southern India (controlling for age, sex, education and alcohol intake). However, this decrease was much more marked in Southern India. In Northern India, age was the only other risk factor associated with reduced eGFR, whereas in Southern India, lower level of formal education was also a risk factor for reduced eGFR (Table 5). We also observed an interaction between the effects of latitude (North/South) and urban/rural residence in association with eGFR<60 (p-value likelihood-ratio test for interaction<0.001). In Northern India, eGFR<60 was not associated with urban/rural residence, and older age was the only factor associated with eGFR<60. In Southern India, rural residence was the strongest risk factor for eGFR<60 but older age and lower level of formal education also increased the risk of eGFR<60 (Table 5).

Sensitivity analyses

For peer review only - http://bmjopen.bmj.com/site/about/guidelines.xhtml

203 We performed a sensitivity analysis including those with ACR>300 (but without hypertension or diabetes, n=33) as we were concerned that those with CKDu might develop proteinuria at more advanced CKD stages. However, this did not alter the mean eGFR (mean eGFR among the overall study population=105.0±17.8, mean eGFR in this sensitivity analysis =105.0±17.8), nor the estimated prevalence of eGFR<60 (prevalence among the overall study population=1.6%; prevalence in this sensitivity analysis =1.7%). The findings on risk factors were also similar to the findings from the primary analyses (supplementary material, Table S3).

209 Given concerns about potentially different thresholds to define diabetes and high blood pressure in different ethnic groups (Herman 2009; Modesti et al. 2016), we performed a further sensitivity analysis including fasting plasma glucose, HbA1c and systolic blood pressure in the multivariate model (even though there is evidence for both causation and reverse causation between these factors and CKD (Verhave et al. 2005)). Systolic blood pressure and fasting plasma glucose were associated with reduced eGFR in this non diabetic population, but inclusion of these variables did not alter the coefficients for the associations with other risk factors observed in the primary analysis (supplementary material, Table S4). HbA1c was associated with eGFR<60 in this non diabetic population but inclusion of this variable did not alter the OR for other risk factors observed in the primary analysis (supplementary material, Table S4). Therefore, although the relationship between sub-clinical diabetes and impaired kidney function requires further prospective investigation, there is no evidence that the excess risk of low eGFR (i.e. lower mean eGFR and higher prevalence of eGFR<60) in rural Southern India is associated with either impaired fasting glucose or higher blood pressure.

221 **DISCUSSION**

222 We report the distribution of eGFR in people without diabetes, hypertension or heavy proteinuria and estimate the prevalence of CKDu in our study population, including participants from urban and rural settings. We found that the rural population from Southern India (Vishakhapatnam district) had the highest risk of low eGFR (lower mean eGFR and higher prevalence of eGFR<60). In Southern India, rural residence, older age and lower education were risk factors for decreased eGFR, and there was also some evidence for higher risks in males. In Northern India, older age was the only risk factor for low eGFR. This is the first population-based evidence, using standardised methods, which indicates that CKDu is present in India and is not confined to Central America and Sri Lanka.

For peer review only - http://bmjopen.bmj.com/site/about/guidelines.xhtml

As in Central America, the risk of low eGFR was higher in rural settings than in urban settings. This is in concordance with a previous study from Hyderabad (India), that has provided evidence of a higher risk of low eGFR in a rural population compared to urban-migrant and urban population (Bailey et al. 2013), and with various studies from other LMICs that have provided evidence of clusters of CKDu among the rural population (Correa-Rotter, Wesseling, and Johnson 2014; Jayatilake et al. 2013). Exposure to some of the suggested potential risk factors for CKDu such as agricultural work and agrochemical exposure, amongst others (Lunyera et al. 2016), may be greater in rural settings. Such exposures may also differ between Southern and Northern India, and potentially explain the differences observed between these areas. The associations between urban/rural residence and lower mean eGFR was much more marked in Southern India than in Northern India, and the associations between urban/rural residence and eGFR<60 was only observed in Southern India. The higher prevalence ratio (for eGFR<60) in the working age population compared to older age groups is consistent with the hypothesis that deceased in eGFR could be potentially explained by occupational exposures. The suggestive sex differences may also support this hypothesis. However, we did not have detailed data on occupation that allowed us to explore these associations in greater detail.

The higher risk of low eGFR in Southern India (Chennai and Vishakhapatnam districts) observed in our study is consistent with the clusters of CKDu cases previously reported in the Southern Indian states of Andhra Pradesh and Odisha (Chatterjee 1026; Ganguli 2016; Reddy and Gunasekar 2013). Visakhapatnam district (state of Andhra Pradesh) and Chennai district (state of Tamil Nadu) have a similar climate than these areas where CKDu clusters have previously reported (Peel, Finlayson, and McMahon 2007). In these districts, mean temperatures range from 18 ºC to 37 ºC and rainfall occurs mainly between June and December (Norwegian Meteorological Institute and the Norwegian Broadcasting Corporation n.d.). On the other hand, sites from Norther India included in the study (Delhi (state of Delhi), Sonipat and Faridabad (Haryana state)), have a different climate. In these districts mean temperature ranges from 8 ºC to 39 ºC and precipitation occurs mainly between July and August (Norwegian Meteorological Institute and the Norwegian Broadcasting Corporation n.d.). A previous study conducted in Costa Rica found a spatial correlation between rates of CKD mortality and temperature and rainfall (Catharina Wesseling et al. 2015).

About 5% of the rural population of Vishakhapatnam (Andra Pradesh, Southern India) without diabetes, hypertension or proteinuria had eGFR<60. This figure is almost as high as the prevalence observed in the USA (i.e. 6.7%) including people with diabetes, hypertension or proteinuria (Levey and Coresh 2012). Moreover, the

For peer review only - http://bmjopen.bmj.com/site/about/guidelines.xhtml

estimates of GFR in our study are likely to be underestimated. The CKD-EPI equation has been standardised for the white and Afro-American population (Levey et al. 2009), but its validity for other ethnic groups has been questioned (Eastwood et al. 2010; Teo et al. 2011). Previous studies using CKD-EPI equation to estimate GFR in Indian populations reported mean eGFR values similar to the mean eGFR reported in our study (i.e. 104.9 ± 25.52 ml/min/1.73 m^2) (Singh et al. 2013). However, two studies conducted among healthy kidney donors in India (population similar to those included in this analysis) have reported mean (measured) GFR between 81.4 and 95.5 ml/min per 1.73 m^2 (Barai et al. 2005; Srinivas et al. 2008), suggesting that the CKD-EPI equation substantially overestimates eGFR in the Indian population. Therefore, the prevalence of eGFR<60 observed in this study is likely to be substantially underestimated (although this is unlikely to have biased the internal comparisons, e.g. between urban and rural settings). The use of a conservative definition of the population susceptible to CKDu, may have also underestimated the prevalence of eGFR<60 in our study, as the population with diabetes, hypertension or glomerular disease may also have reduced eGFR due to other ('unknown') causes. To estimate the actual prevalence of reduced eGFR, future studies should include validated methods to estimate GFR in the Indian population. We were concerned that the validity of CKD-EPI among the Indian population may be also compromised by differences in muscular mass and meat consumption between population groups within India. We adjusted the analyses for fat free mass and vegetarianism, but this did not alter the results, suggesting no confounding effect by these variables.

Our study has at least three potential limitations. First, we only had one measure of eGFR, and therefore we could not differentiate acute kidney injury (AKI) from CKD. This is a common limitation in epidemiological studies, as it is challenging to obtain more than one measure of eGFR at least 3 months apart in large population-based investigations. Therefore, we may have misclassified some cases of AKI as reduced eGFR, and therefore overestimate the prevalence of this condition. Nevertheless, there is no a priori reason to think that potential misclassification was different according to the evaluated risks factors. Second, the three population-based studies included in this analysis used different sampling strategies. CARRS and UDAY studies included only one man and one woman from all the eligible participants of selected households, whereas ICMR-CHD included all eligible adults from each selected household. This could have slightly biased our results (including our prevalence estimates) if risk factors potentially associated with CKDu were different between households inhabited only by a man and a women or by extended families. Third, information on other potential risk factors for CKDu, such as infections by leptospora or hantavirus infection, or use of nonsteroidal anti-inflammatory drugs (NSAIDs) was not available.

For peer review only - http://bmjopen.bmj.com/site/about/guidelines.xhtml

The main strengths of the study are the use of a random selection of population-based participants and a large sample size including participants from different areas of India (urban and rural, and Northern and Southern India). Moreover, we used the definitions proposed in DRGREE study (Caplin et al. 2017), that aims to allow international comparisons of CKDu prevalence and help in the description of risk factors and in identifying the causes and mechanisms leading to CKDu.

In conclusion, our findings indicate that reduced eGFR, consistent with the definition of CKDu, is common in rural settings of Southern India (Vishakhapatnam district). This results support the hypothesis that the epidemic of CKDu, initially described in agricultural communities of Central America and Sri Lanka, may be common in other rural communities of tropical/subtropical countries. This has important implications for global health, since it indicates that CKDu may have a substantial public health burden globally that has been previously unrecognised. Population-based studies in other tropical/subtropical countries are required to assess the global patterns of burden of disease from CKDu (Caplin et al. 2017).

AUTHOR CONTRIBUTIONS AND ACKNOWLEDGEMENTS

CO-G, BC, NP and DP designed the analysis; RS, SM, PPA, DK and SG let the collection of the original data; all authors participated in interpretation and discussion of results; CO-G, BC and NP drafted the manuscript. All authors contributed to critical reading of the report, provided comments and suggested revisions, and approved the final version for publication.

We thank Manolis Kogevinas for his comments on the advanced version of the manuscript.

CONFLICTS OF INTERESTS

The authors declare that they have no competing interests

DATA SHARING STATEMENT

The datasets used and/or analysed during the current study are available from Public Health Foundation of India (PHFI) on reasonable request. Interested investigators should contact PHFI. Computing code can be obtained from the corresponding author.

For peer review only - http://bmjopen.bmj.com/site/about/guidelines.xhtml

REFERENCES

Anand, Shuchi, Roopa Shivashankar, Mohammed K Ali, Dimple Kondal, B Binukumar, Maria E Montez-rath, Vamadevan S Ajay, et al. 2015. "Prevalence of Chronic Kidney Disease in Two Major Indian Cities and Projections for Associated Cardiovascular Disease." *Kidney International* 88 (1). Nature Publishing Group:178–85. https://doi.org/10.1038/ki.2015.58.

Bailey, Phillippa K, Charles R V Tomson, Sanjay Kinra, Shah Ebrahim, K V Radhakrishna, Hannah Kuper, Dorothea Nitsch, and Yoav Ben-shlomo. 2013. "The Effect of Rural-to-Urban Migration on Renal Function in an Indian Population : Cross-Sectional Data from the Hyderabad Arm of the Indian Migration Study."

Barai, Sukanta, G P Bandopadhayaya, C D Patel, Manish Rathi, R Kumar, D Bhowmik, S Gambhir, N Gopendro Singh, A Malhotra, and K D Gupta. 2005. "Do Healthy Potential Kidney Donors in India Have an Average Glomerular Filtration Rate of 81.4 Ml/min?" *Nephron. Physiology* 101 (1):p21-6. https://doi.org/10.1159/000086038.

Barsoum, Rashad S. 2013. "Burden of Chronic Kidney Disease: North Africa." *Kidney International Supplements* 3 (2):164–66. https://doi.org/10.1038/kisup.2013.5.

Caplin, Ben, Kristina Jakobsson, Jason Glaser, Dorothea Nitsch, Vivekanand Jha, Ajay Singh, Ricardo Correa-Rotter, and Neil Pearce. 2017. "International Collaboration for the Epidemiology of eGFR in Low and Middle Income Populations - Rationale and Core Protocol for the Disadvantaged Populations eGFR Epidemiology Study (DEGREE)." *BMC Nephrology* 18 (1):1. https://doi.org/10.1186/s12882-016-0417-1.

Chatterjee, Rhitu. 1026. "Occupational Hazard." *Science*, 1026.

Correa-Rotter, Ricardo, Catharina Wesseling, and Richard J Johnson. 2014. "CKD of Unknown Origin in Central America: The Case for a Mesoamerican Nephropathy." *American Journal of Kidney Diseases : The Official Journal of the National Kidney Foundation* 63 (3):506–20. https://doi.org/10.1053/j.ajkd.2013.10.062.

Dare, Anna J, Sze Hang Fu, Jayadeep Patra, Peter S Rodriguez, J S Thakur, Prabhat Jha, J Coresh, et al. 2017. "Renal Failure Deaths and Their Risk Factors in India 2001–13: Nationally Representative Estimates from the Million Death Study." *The Lancet Global Health* 5 (1). The Author(s). Published by Elsevier Ltd. This is an Open Access article under the CC BY license:e89–95. https://doi.org/10.1016/S2214-109X(16)30308-4.

Eastwood, J. B., S. M. Kerry, J. Plange-Rhule, F. B. Micah, S. Antwi, F. G. Boa, D. Banerjee, and F. P. Cappuccio. 2010. "Assessment of GFR by Four Methods in Adults in Ashanti, Ghana: The Need for an eGFR Equation for Lean African Populations." *Nephrology Dialysis Transplantation* 25 (7):2178–87. https://doi.org/10.1093/ndt/gfp765.

Ganguli, Anirban. 2016. "Uddanam Nephropathy/Regional Nephropathy in India: Preliminary Findings and a Plea for Further Research." *American Journal of Kidney Diseases*. National Kidney Foundation, Inc., 2–6. https://doi.org/10.1053/j.ajkd.2016.04.012.

Garcia-Garcia, Guillermo, Vivekanand Jha, and World Kidney Day Steering Committee. 2015. "Environmental and Occupational Factors in CKD." *Occupational and Environmental Medicine* 72 (3):238. https://doi.org/10.1136/oemed-2015-102859.

Greenland, Sander, Rhian Daniel, Neil Pearce, Sander Greenland, Rhian Daniel, and Neil Pearce. 2016. "Outcome Modelling Strategies in Epidemiology: Traditional Methods and Basic Alternatives." *International Journal of Epidemiology*, no. April:1–11. https://doi.org/10.1093/ije/dyw040.

Herman, William H. 2009. "Do Race and Ethnicity Impact Hemoglobin A1c Independent of Glycemia?" *Journal of Diabetes Science and Technology* 3 (4):656–60. https://doi.org/10.1177/193229680900300406.

Jayasumana, Channa, Priyani Paranagama, Suneth Agampodi, Chinthaka Wijewardane, Sarath Gunatilake, and Sisira Siribaddana. 2015. "Drinking Well Water and Occupational Exposure to Herbicides Is Associated with Chronic Kidney Disease, in Padavi-Sripura, Sri Lanka." *Environmental Health : A Global Access Science Source* 14 (1):6. https://doi.org/10.1186/1476-069X-14-6.

Jayatilake, Nihal, Shanthi Mendis, Palitha Maheepala, and Firdosi R Mehta. 2013. "Chronic Kidney Disease of Uncertain Aetiology: Prevalence and Causative Factors in a Developing Country." *BMC Nephrology* 14 (1). BMC Nephrology:180. https://doi.org/10.1186/1471-2369-14-180.

Jha, Vivekanand, and Gopesh Modi. 2017. "Uncovering the Rising Kidney Failure Deaths in India." *The Lancet*

For peer review only - http://bmjopen.bmj.com/site/about/guidelines.xhtml

Global Health 5 (1). The Author(s). Published by Elsevier Ltd. This is an Open Access article under the CC BY-NC-ND license:e14–15. https://doi.org/10.1016/S2214-109X(16)30299-6.

Lebov, Jill F, Eliette Valladares, Rodolfo Pena, Edgar M Pena, Scott L Sanoff, Efren Castellon Cisneros, Romulo E Colindres, Douglas R Morgan, and Susan L Hogan. 2015. "A Population-Based Study of Prevalence and Risk Factors of Chronic Kidney Disease in Leon, Nicaragua." *Canadian Journal of Kidney Health and Disease* 2. ???6. https://doi.org/10.1186/s40697-015-0041-1.

Levey, Andrew S., and Josef Coresh. 2012. "Chronic Kidney Disease." *The Lancet* 379 (9811). Elsevier Ltd:165–80. https://doi.org/10.1016/S0140-6736(11)60178-5.

Levey, Andrew S, Lesley A Stevens, Christopher H Schmid, Yaping Lucy Zhang, Alejandro F Castro, Harold I Feldman, John W Kusek, et al. 2009. "A New Equation to Estimate Glomerular Filtration Rate." *Annals of Internal Medicine* 150 (9):604–12. http://www.ncbi.nlm.nih.gov/pubmed/19414839.

Lunyera, Joseph, Dinushika Mohottige, Megan von Isenburg, Marc Jeuland, Uptal D. Patel, and John W. Stanifer. 2016. "CKD of Uncertain Etiology: A Systematic Review." *Clinical Journal of the American Society of Nephrology* 11 (3):379–85. https://doi.org/10.2215/CJN.07500715.

Minshawy, Osama El, Tawfik Ghabrah, and Eman El Bassuoni. 2014. "End-Stage Renal Disease in Tabuk Area, Saudi Arabia: An Epidemiological Study." *Saudi Journal of Kidney Diseases and Transplantation : An Official Publication of the Saudi Center for Organ Transplantation, Saudi Arabia* 25 (1):192–95. http://www.ncbi.nlm.nih.gov/pubmed/24434411.

Modesti, Pietro Amedeo, Gianpaolo Reboldi, Francesco P Cappuccio, Charles Agyemang, Giuseppe Remuzzi, Stefano Rapi, Eleonora Perruolo, and Gianfranco Parati. 2016. "Panethnic Differences in Blood Pressure in Europe: A Systematic Review and Meta-Analysis." *PloS One* 11 (1):e0147601. https://doi.org/10.1371/journal.pone.0147601.

Mohan, S;, P; Jarhyan, S; Ghosh, SV; Nikhil, R; Gupta, R; Rana, C; Malhotra, et al. 2017. "UDAY: Protocol of a Comprehensive Diabetes and Hypertension Prevention and Management Program in India." *BMJ Open.* In Press.

Nair, Manisha, Mohammed K Ali, Vamadevan S Ajay, Roopa Shivashankar, Viswanathan Mohan, Rajendra Pradeepa, Mohan Deepa, et al. 2012. "CARRS Surveillance Study: Design and Methods to Assess Burdens from Multiple Perspectives." *BMC Public Health* 12 (1). BMC Public Health:701. https://doi.org/10.1186/1471-2458-12-701.

Norwegian Meteorological Institute and the Norwegian Broadcasting Corporation. n.d. "Yr." Accessed January 19, 2018. https://www.yr.no.

Peel, M. C., B. L. Finlayson, and T. A. McMahon. 2007. "Updated World Map of the Koppen-Geiger Climate Classification." *Hydrology and Earth System Sciences* 11:1633–1644.

Peraza, Sandra, Catharina Wesseling, Aurora Aragon, Ricardo Leiva, Ramón Antonio García-Trabanino, Cecilia Torres, Kristina Jakobsson, Carl Gustaf Elinder, and Christer Hogstedt. 2012. "Decreased Kidney Function among Agricultural Workers in El Salvador." *American Journal of Kidney Diseases : The Official Journal of the National Kidney Foundation* 59 (4):531–40. https://doi.org/10.1053/j.ajkd.2011.11.039.

Prabhakaran, Dorairaj, Ambuj Roy, Pradeep A. Praveen, Lakshmy Ramakrishnan, Ruby Gupta, Ritvik Amarchand, Dimple Kondal, et al. 2017. "20-Year Trend of Cardiovascular Disease Risk Factors." *Global Heart*. https://doi.org/10.1016/j.gheart.2016.11.004.

Rajapurkar, Mohan M, George T John, Ashok L Kirpalani, Georgi Abraham, Sanjay K Agarwal, Alan F Almeida, Sishir Gang, et al. 2012. "What Do We Know about Chronic Kidney Disease in India: First Report of the Indian CKD Registry." *BMC Nephrology* 13 (1):10. https://doi.org/10.1186/1471-2369-13-10.

Reddy, D. V., and A. Gunasekar. 2013. "Chronic Kidney Disease in Two Coastal Districts of Andhra Pradesh, India: Role of Drinking Water." *Environmental Geochemistry and Health* 35 (4):439–54. https://doi.org/10.1007/s10653-012-9506-7.

Robey, R Brooks. 2014. "Cyclical Dehydration-Induced Renal Injury and Mesoamerican Nephropathy: As Sweet by Any Other Name?" *Kidney International* 86 (2):226–29. https://doi.org/10.1038/ki.2014.47.

Seck, Sidy Mohamed, Dominique Doupa, Lamine Gueye, and Charles Abdou Dia. 2014. "Prevalence of Chronic Kidney Disease and Associated Factors in Senegalese Populations: A Community-Based Study in Saint-Louis." *Nephro-Urology Monthly* 6 (5):e19085. https://doi.org/10.5812/numonthly.19085.

Singh, Ajay K., Youssef MK Farag, Bharati V. Mittal, Kuyilan Karai Subramanian, Sai Ram Keithi Reddy,

For peer review only - http://bmjopen.bmj.com/site/about/guidelines.xhtml

Vidya N. Acharya, Alan F. Almeida, et al. 2013. “Epidemiology and Risk Factors of Chronic Kidney Disease in India – Results from the SEEK (Screening and Early Evaluation of Kidney Disease) Study.” *BMC Nephrology* 14 (1). BMC Nephrology:114. https://doi.org/10.1186/1471-2369-14-114.

Srinivas, Sanjay, Rajeev A Annigeri, Muthu Krishna Mani, Budithi Subba Rao, Prakash C Kowdle, and Rajagopalan Seshadri. 2008. “Estimation of Glomerular Filtration Rate in South Asian Healthy Adult Kidney Donors.” *Nephrology (Carlton, Vic.)* 13 (5):440–46. https://doi.org/10.1111/j.1440-1797.2008.00967.x.

Teo, Boon Wee, Hui Xu, Danhua Wang, Jialiang Li, Arvind Kumar Sinha, Borys Shuter, Sunil Sethi, and Evan J C Lee. 2011. “GFR Estimating Equations in a Multiethnic Asian Population.” *American Journal of Kidney Diseases : The Official Journal of the National Kidney Foundation* 58 (1):56–63. https://doi.org/10.1053/j.ajkd.2011.02.393.

Torres, Cecilia, Aurora Aragón, Marvin González, Indiana López, Kristina Jakobsson, Carl-Gustaf Elinder, Ingvar Lundberg, and Catharina Wesseling. 2010. “Decreased Kidney Function of Unknown Cause in Nicaragua: A Community-Based Survey.” *American Journal of Kidney Diseases : The Official Journal of the National Kidney Foundation* 55 (3):485–96. https://doi.org/10.1053/j.ajkd.2009.12.012.

Verhave, Jacobien C., Hans L. Hillege, Johannes G M Burgerhof, Ron T. Gansevoort, Dick De Zeeuw, and Paul E. De Jong. 2005. “The Association between Atherosclerotic Risk Factors and Renal Function in the General Population.” *Kidney International* 67 (5):1967–73. https://doi.org/10.1111/j.1523-1755.2005.00296.x.

Wesseling, C, J Crowe, C Hogstedt, K Jakobsson, R Lucas, and D Wegman. 2013. “Mesoamerican Nephropathy: Report from the First International Research Workshop on MeN.” Heredia, Costa Rica. http://www.regionalnephropathy.org/wp-content/uploads/2013/04/Technical-Report-for-Website-Final.pdf.

Wesseling, Catharina, Berna van Wendel de Joode, Jennifer Crowe, Ralf Rittner, Negin A Sanati, Christer Hogstedt, and Kristina Jakobsson. 2015. “Mesoamerican Nephropathy: Geographical Distribution and Time Trends of Chronic Kidney Disease Mortality between 1970 and 2012 in Costa Rica.” *Occupational and Environmental Medicine* 72 (10):714–21. https://doi.org/10.1136/oemed-2014-102799.

World Health Organization. 2015. “STEPS Manual.” 2015.

For peer review only

For peer review only - http://bmjopen.bmj.com/site/about/guidelines.xhtml

TABLES

Table 1. Design and methods of the three studies included in the current analysis

	CARRS		UDAY				ICMR-CHD	
**Latitude (North/South)**	North	South	North		South		North	
**Residence (Urban/Rural)**	Urban		Urban	Rural	Urban	Rural	Urban	Rural
**District (and State)**	Delhi (state of Delhi)	Chennai (state of Tamil Nadu)	Sonipat (state of Haryana)		Vishakhapatnam (state of Andhra Pradesh)		National Capital Territory of Delhi (state of Delhi)	Faridabad (state of Haryana)
**Household sampling**	Multistage cluster random (wards - census enumeration blocks - households)		Multistage cluster random (Census Enumeration blocks (urban) or villages (rural) - households)				Multistage cluster random (wards - census enumeration blocks - households)	Simple cluster random (based on Health and Demographic Surveillance System)
**Individual sampling**	1 man and 1 woman from each household (selected by Kish method, (World Health Organization. 2015).)		1 man and 1 woman from each household (selected by Kish method, (World Health Organization. 2015).)				All adults	
**Age groups included**	≥ 20		≥ 30				≥ 30	
**Exclusion criteria**	Pregnant, bedridden and participants who were unable to comprehend the questionnaires due cognitive deficiencies were excluded							
**Study period**	October 2010 - November 2011		July 2014 - December 2014				August 2010 - January 2012	
**Laboratory** [a]	PHFI [b]	MDRF [c]	PHFI [b]				PHFI [b]	

[a] Study laboratories participated in Randox International Quality Assurance Scheme (RIQAS) for clinical chemistry and HbA1c during the entire study periods. [b] Public Health Foundation of India; [c] Madras Diabetes Research Foundation

For peer review only - http://bmjopen.bmj.com/site/about/guidelines.xhtml

454 **Table 2**. Sociodemographic and anthropometric characteristics of study participants (population without
455 diabetes, hypertension or heavy proteinuria)

Variable	n (%) [a] n=12,500	eGFR	eGFR categories, n(%) [b]		
		mean (SD)	≥90	90-60	<60
**Socio-demographic**					
Age (years)					
<39	6121 (49)	113.8 (14.6)	5656 (92)	443 (7)	22 (0)
40-49	3476 (28)	102.5 (14.2)	2864 (82)	572 (16)	40 (1)
50-59	1706 (14)	93.9 (14.3)	1163 (68)	503 (29)	40 (2)
60-69	893 (7)	85.3 (16.2)	463 (52)	368 (41)	62 (7)
>=70	304 (2)	77.5 (15.1)	62 (20)	201 (66)	41 (13)
Sex					
Female	7066 (57)	107.9 (17.1)	6039 (85)	945 (13)	82 (1)
Male	5434 (43)	101.3 (17.9)	4169 (77)	1142 (21)	123 (2)
Education (number completed years)					
0	2820 (23)	100.7 (19.0)	2165 (77)	551 (20)	104 (4)
≤5	1709 (14)	105.9 (17.3)	1412 (83)	273 (16)	24 (1)
6-≤10	4817 (39)	107.2 (16.8)	4095 (85)	675 (14)	47 (1)
> 10	3154 (25)	105.0 (17.5)	2536 (80)	588 (19)	30 (1)
Area [c]					
Urban	8494 (68)	107.8 (16.1)	7247 (85)	1171 (14)	76 (1)
Rural	4006 (32)	99.0 (18.0)	2961 (74)	916 (23)	129 (3)
Latitude [d]					
North	6263 (50)	103.0 (17.2)	4967 (79)	1197 (19)	99 (2)
South	6237 (50)	107.0 (18.1)	5241 (84)	890 (14)	106 (2)
**Life-style factors**					
Current tobacco consumption					
No	9357 (75)	106.8 (17.3)	7836 (84)	1406 (15)	115 (1)
Yes	3143 (25)	99.8 (18.1)	2372 (75)	681 (22)	90 (3)
Alcohol consumption ever					
No	10094 (81)	105.9 (17.4)	8362 (83)	1589 (16)	143 (1)
Yes	2406 (19)	101.1 (18.5)	1846 (77)	498 (21)	62 (3)
Vegetarian					

For peer review only - http://bmjopen.bmj.com/site/about/guidelines.xhtml

No	7972 (64)	107.0 (18.0)	6690 (84)	1154 (14)	128 (2)
Yes	4528 (36)	101.6 (16.6)	3518 (78)	933 (21)	77 (2)
**Biological factors**					
Body mass index (kg/m^2)					
Underweight (≤18.5)	5879 (47)	104.2 (17.9)	4734 (81)	1029 (18)	116 (2)
Normal (>18.5 - ≤25)	1576 (13)	104.7 (19.3)	1283 (81)	257 (16)	36 (2)
Overweight (>25 - ≤30)	3313 (27)	105.0 (16.9)	2710 (82)	568 (17)	35 (1)
Obese (>30)	1150 (9)	105.5 (16.4)	948 (82)	194 (17)	8 (1)
Missing data	582 (5)		533 (92)	39 (7)	10 (2)
Fat free mass (kg/m^2)					
1st tertile (≤37)	3746 (30)	106.6 (18.1)	3146 (84)	532 (14)	68 (2)
2nd tertile (>37 - <45)	3801 (30)	105.9 (17.2)	3145 (83)	601 (16)	55 (1)
3rd tertile (≤45)	3834 (31)	102.1 (17.0)	2981 (78)	801 (21)	52 (1)
Missing data	1119 (9)		936 (84)	153 (14)	30 (3)

[a] Percentages in columns; [b] percentages in rows; [c] Urban areas include Delhi, Chennai and Sonipat district. Rural areas include Sonipat, Vishakhapatnam and Faridabad districts; [d] North areas include Delhi, Sonipat and Faridabad district. South areas include Chennai and Vishakhapatnam districts.

For peer review only - http://bmjopen.bmj.com/site/about/guidelines.xhtml

Table 3. Associations between sociodemographic and anthropometric characteristics and eGFR and eGFR<60

	eGFR	eGFR<60
Variable	Coefficient (95% CI) [a]	OR (95% CI) [a]
Age (years) [b]		
<39		1
40-49	-11 (-12 - -10)	3.1 (1.9 - 5.3)
50-59	-19 (-20 - -19)	6.4 (3.8 - 10)
60-69	-28 (-29 - -27)	20 (12 - 32)
>=70	-35 (-37 - -33)	39 (23 - 67)
Sex [c]		
Female		1
Male	-3.5 (- 4.0 - -3.1)	1.3 (0.99 - 1.8)
Education (number of completed years)		
0		1
≤5	1.9 (1.1 - 2.8)	0.41 (0.26 - 0.65)
6-≤10	1.3 (0.6 - 1.9)	0.36 (0.25 - 0.53)
> 10	-1.9 (-2.6 - -1.1)	0.40 (0.26 - 0.62)
Area [d]		
Urban		1
Rural	-3.8 (-4.4 - -3.3)	2.4 (1.8 - 3.2)
Latitude [e]		
North		1
South	0.86 (0.37 - 1.3)	1.5 (1.2 - 2.1)
Current tobacco consumption		
No		1
Yes	0.38 (-0.26 - 1.0)	1.4 (1.0 - 1.9)
Alcohol consumption ever		
No		1
Yes	-0.81 (-1.5 - -0.08)	1.6 (1.09 - 2.3)
Vegetarian		
No		1
Yes	-0.99 (-1.5 - -0.47)	0.65 (0.48 - 0.88)

For peer review only - http://bmjopen.bmj.com/site/about/guidelines.xhtml

Body mass index (kg/m^2) [g]		
Underweight (≤18.5)	3.0 (2.2 - 3.7)	0.81 (0.55 - 1.2)
Normal (>18.5 - ≤25)		1
Overweight (>25 - ≤30)	-0.75 (-1.3 - -0.16)	0.7 (0.46 - 1.0)
Obese (>30)	-0.71 (-1.6 - 0.17)	0.47 (0.23 - 0.98)
Fat free mass (kg/m^2) [g]		
1st tertile (≤37)		1
2nd tertile (>37 - <45)	-0.91 (-1.5 - -0.28)	0.69 (0.47 - 1.0)
3rd tertile (≤45)	-3.9 (-4.8 - -3.0)	0.49 (0.31 - 0.80)

461 [a] Adjusted for age and sex; [b] Adjusted just for sex; [c] Adjusted just for age; [d] Urban areas include Delhi,
462 Chennai and Sonipat district. Rural areas include Sonipat, Vishakhapatnam and Faridabad districts; [e]
463 North areas include Delhi, Sonipat and Faridabad district. South areas include Chennai and
464 Vishakhapatnam districts.

For peer review only - http://bmjopen.bmj.com/site/about/guidelines.xhtml

465 **Table 4**. Multiple regression analyses of sociodemographic characteristics associated with eGFR and eGFR<6). Models adjusting for all variables, plus models further
466 adjusted for fat free mass and vegetarianism.

	eGFR Coefficient (95% CI)			eGFR<60 OR (95% CI)		
Variable	Model 1[a]	Model 2[b]	Model 3[c]	Model 1[a]	Model 2[b]	Model 3[c]
Area [d]						
Urban				1	1	1
Rural	-4.6 (-5.1 - -4.0)	-3.9 (-4.5 - -3.4)	-4.1 (-4.7 - -3.5)	2.0 (1.4 - 2.8)	1.6 (1.1 - 2.3)	1.6 (1.1 - 2.4)
Latitude [e]						
North				1	1	1
South	0.31 (-0.18 - 0.80)	-0.10 (-0.61 - 0.41)	0.26 (-0.37 - 0.89)	1.3 (1.0 - 1.8)	1.60 (1.1 - 2.2)	1.33 (0.86 - 2.0)
Education (number of completed years)						
0				1	1	1
≤5	0.93 (0.01 - 1.8)	1.2 (0.30 - 2.0)	1.2 (0.32 - 2.0)	0.50 (0.31 - 0.80)	0.44 (0.26 - 0.74)	0.45 (0.26 - 0.75)
6-≤10	0.31 (-0.18 - 0.80)	0.21 (-0.49 - 0.91)	0.21 (-0.50 - 0.92)	0.50 (0.34 - 0.75)	0.38 (0.24 - 0.60)	0.39 (0.25 - 0.62)
> 10	-4.0 (-4.6 - -3.0)	-3.8 (-4.6 - -3.0)	-3.8 (-4.6 - -230)	0.68 (0.42 - 1.11)	0.61 (0.36 - 1.0)	0.6 (0.38 - 1.1)
Alcohol consumption ever						

For peer review only - http://bmjopen.bmj.com/site/about/guidelines.xhtml

No				1	1	1
Yes	-0.85 (-1.6 - -0.11)	-0.69 (-1.5 - 0.08)	-0.63 (-1.4 - 0.15)	1.3 (0.88 - 1.9)	1.2 (0.78 - 1.8)	1.1 (0.76 - 1.7)
Sex						
Female				1	1	1
Male	-2.8 (-2.2 - -3.4)	3.0 (2.4 - 3.6)	2.5 (1.9 - 3.2)	0.72 (0.50 - 1.0)	0.67 (0.45 - 1.0)	0.67 (0.43 - 1.0)
Age (years)	-0.91 (-0.93 - -0.89)	-0.91 (-0.93 - -0.89)	-0.91 (-0.94 - -0.89)	1.1 (1.1 - 1.1)	1.1 (1.1 - 1.1)	1.1 (1.1 - 1.1)
Fat free mass (kg/m^2)			-0.04 (-0.06 - -0.02)			1.0 (0.98 - 1.0)
Vegetarian						
No						1
Yes			0.66 (-0.03 - 1.3)			0.74 (0.47 - 1.2)

[a] Model 1: Variables mutually adjusted, n=12,500; [b] Model 2: Variables mutually adjusted. Model excluding missing on fat free mass, n=11,381; [c] Model 3: Variables mutually adjusted. Model includes further adjustment for fat free mass and vegetarianism, n=11,381. [d] Urban areas include Delhi, Chennai and Sonipat district. Rural areas include Sonipat, Vishakhapatnam and Faridabad districts; [e] North areas include Delhi, Sonipat and Faridabad district. South areas include Chennai and Vishakhapatnam districts.

For peer review only - http://bmjopen.bmj.com/site/about/guidelines.xhtml

Table 5. Multivariate analysis of sociodemographic characteristics associated with eGFR and with eGFR<60 according to latitude [a]

	eGFR (n=12,500)		eGFR<60(n=12,500)	
	North (n=6263) [a]	South (n= 6237) [a]	North (n=6263) [a]	South (n= 6237) [a]
Variables	Coefficient (95% CI)	Coefficient (95% CI)	OR (95% CI)	OR (95% CI)
Area [b]				
Urban				
Rural	-1.4 (-2.1 - -0.70)	-7.9 (-8.8 - -7.0)	1	1
Education (number of completed years)			0.88 (0.57 - 1.4)	4.7 (2.5 - 8.8)
0				
≤5	-1.3 (-2.6 - -0.05)	1.0 (-0.06 - 2.2)	1	1
6-≤10	-3.5 (-4.5 - -2.5)	0.28 (-0.74 - 1.3)	1.2 (0.57 - 2.3)	0.40 (0.20 - 0.80)
> 10	-6.9 (-8.0 - -5.9)	-2.8 (-4.0 - -1.7)	1.3 (0.74 - 2.4)	0.35 (0.16 - 0.74)
Alcohol consumption ever			1.3 (0.69 - 2.6)	0.61 (0.24 - 1.6)
No				
Yes	-0.54 (-1.5 - 0.47)	-0.06 (-1.1 - 0.99)	1	1
Sex			1.1 (0.62 - 1.9)	1.3 (0.74 - 2.2)
Female				
Male	0.17 (-0.63 - 0.96)	5.4 (4.5 - 6.3)	1	1
Age (years)	-0.93 (-0.96 - -0.90)	-0.90 (-0.93 - -0.86)	1.0 (0.63 - 1.7)	0.63 (0.36 - 1.1)

** Likelihood ratio test for linear trend <0.05, OR (95% CI)=0.67 (0.50-0.90). [a] North areas include Delhi, Sonipat and Faridabad district. South areas include Chennai and Vishakhapatnam districts. [b] Urban areas include Delhi, Chennai and Sonipat district. Rural areas include Sonipat, Vishakhapatnam and Faridabad districts

For peer review only - http://bmjopen.bmj.com/site/about/guidelines.xhtml

479 **FIGURES LEGENDS**

480 **Figure 1** Study flowchart

481 **Figure 2** Prevalence ratio of eGFR<60 by age group between rural and urban areas

For peer review only

For peer review only - http://bmjopen.bmj.com/site/about/guidelines.xhtml

SUPPLEMENTARY MATERIAL

Content

Table S1. Sociodemographic and anthropometric characteristics of overall study participants (prior to exclusion of population with diabetes, hypertension and proteinuria)

Table S2. Associations between sociodemographic and anthropometric characteristics and estimated glomerular filtration rate (eGFR) and eGFR<60 by sex

Table S3. Multiple regression analysis of sociodemographic and anthropometric characteristics associated with eGFR and eGFR<60 including study participants with proteinuria (but without diabetes or hypertension)

Table S4. Multiple regression analysis of sociodemographic and anthropometric characteristics associated with eGFR and eGFR<60 including fasting plasma glucose, HbA1c and systolic blood pressure

For peer review only

For peer review only - http://bmjopen.bmj.com/site/about/guidelines.xhtml

494 **Table S1.** Sociodemographic and anthropometric characteristics of overall study participants (prior to
495 exclusion of population with diabetes, hypertension and proteinuria)

496

**Variable**	**n (%)[*] n=12 500**	**eGFR**		**eGFR categories, n(%)[**]**			
		**mean (SD)**	**p-value[§]**	**≥90**	**90-60**	**<60**	**p-value[§§]**
**Socio-demographic factors**							
Age (years)							
<39	9007 (36)	112.9 (14.9)	<0.001	8248 (92)	716 (8)	43 (0)	<0.001
40-49	6924 (28)	101.9 (14.8)		5617 (81)	1215 (18)	92 (1)	
50-59	4524 (18)	92.9 (15.2)		2997 (66)	1378 (30)	149 (3)	
60-69	3045 (12)	82.8 (17.1)		1410 (46)	1315 (43)	320 (11)	
>=70	1274 (5)	72.0 (17.3)		164 (13)	806 (63)	304 (24)	
Sex							
Female	13433 (54)	102.6 (19.5)		10404 (77)	2585 (19)	444 (3)	<0.001
Male	11341 (46)	97.7 (19.3)	0.33	8032 (71)	2845 (25)	464 (4)	
Education (number of years)							
0	4794 (19)	97.7 (20.2)	<0.001	3458 (72)	1075 (22)	261 (5)	<0.001
5	3194 (13)	101.7 (19.4)		2456 (77)	625 (20)	113 (4)	
10	8855 (36)	103.2 (18.9)		6995 (79)	1620 (18)	240 (3)	
> 10	6358 (26)	100.0 (19.2)		4638 (73)	1538 (24)	182 (3)	
Missing data	1573 (6)			889 (57)	572 (36)	112 (7)	
Area [e]							
Urban	17732 (72)	102 (19.5)	0.03	13577 (77)	3602 (20)	553 (3)	<0.001
Rural	7042 (28)	96.3 (19.1)		4859 (69)	1828 (26)	355 (5)	
Latitude [f]							
North	13570 (55)	98.1 (19.1)	<0.001	9599 (71)	3439 (25)	532 (4)	<0.001
South	11204 (45)	103.1 (19.7)		8837 (79)	1991 (18)	376 (3)	
**Life-style factors**							
Current smoking							
No	18402 (74)	101.5 (19.6)	0.01	13920 (76)	3838 (21)	644 (3)	<0.001
Yes	6372 (26)	97.1 (19.1)		4516 (71)	1592 (25)	264 (4)	
Alcohol consumption ever							
No	19588 (79)	100.9 (19.6)	0.01	14671 (75)	4203 (21)	714 (4)	0.01
Yes	5186 (21)	98.5 (19.1)		3765 (73)	1227 (24)	194 (4)	
Vegetarian							
No	15043 (61)	102.7 (19.7)	<0.001	11721 (78)	2835 (19)	487 (3)	<0.001
Yes	9731 (39)	96.8 (18.9)		6715 (69)	2595 (27)	421 (4)	
**Biological factors**							
Body mass index (kg/m^2)							
Underweight (≤18.5)	10297 (42)	100.1 (19.6)	<0.001	7626 (74)	2284 (22)	387 (4)	0.01
Normal (>18.5 - ≤25)	2403 (10)	101.58 (20.5)		1838 (76)	471 (20)	94 (4)	
Overweight (>25 - ≤30)	7221 (29)	99.9 (18.8)		5309 (74)	1680 (23)	232 (3)	
Obese (>30)	3286 (13)	99.3 (19.2)		2392 (73)	766 (23)	128 (4)	
Missing data	1567 (6)			1271 (81)	229 (15)	67 (4)	
Fat free mass (kg/m^2)							
1st tertile (≤37)	7141 (29)	101.9 (20.1)	<0.001	5481 (77)	1381 (19)	279 (4)	<0.001

For peer review only - http://bmjopen.bmj.com/site/about/guidelines.xhtml

2nd tertile (>37 - <45)	7141 (29)	101.3 (19.1)		5419 (76)	1487 (21)	235 (3)
3rd tertile (≤45)	7141 (29)	98.3 (18.6)		5110 (72)	1797 (25)	234 (3)
Missing data	3351 (14)			2426 (72)	765 (23)	160 (5)

[a] Percentages in columns; [b] percentages in rows; [c] Bartlett's test for equal variance; [d] Chi-square test; [e] Urban areas include Delhi, Chennai and Sonipat district. Rural areas include Sonipat, Vishakhapatnam and Faridabad districts; [f] North areas include Delhi, Sonipat and Faridabad district. South areas include Chennai and Vishakhapatnam districts.

For peer review only

For peer review only - http://bmjopen.bmj.com/site/about/guidelines.xhtml

Table S2. Associations between sociodemographic and anthropometric characteristics and estimated glomerular filtration rate (eGFR) and eGFR<60 by sex

	Men, n=5 434			Women, n=7 066		
**Variable**		**eGFR**	**eGFR<60**		**eGFR**	**eGFR<60**
	**n (%)**	**Coefficient (95%CI) [a]**	**OR (95% CI) [a]**	**n (%)**	**Coefficient (95%CI) [a]**	**OR (95% CI) [a]**
Age (years) [b]						
<39	2335 (43)			3786 (54)		
40-49	1568 (29)	-9.0 (-10 - -8.0)	2.4 (1.2 - 4.6)	1908 (27)	-12 (-13 --12)	4.5 (1.9 - 10)
50-59	843 (16)	-17 (-18 - -16)	3.8 (1.9 - 7.7)	863 (12)	-21 (-22 --20)	12 (5.2 - 27)
60-69	479 (9)	-25 (-27 - -24)	13 (7.0 - 24)	414 (6)	-30 (-31 --29)	33 (15 - 73)
>=70	209 (4)	-34 (-36 - -32)	31 (16 - 59)	95 (1)	-35 (-38 --32)	43 (16 - 118)
Education (number of completed years)						
0	823 (15)		1	1997 (28)		1
≤5	703 (13)	3.3 (1.8 - 4.7)	0.24 (0.13 - 0.46)	1006 (14)	0.73 (-0.27 -1.7)	0.81 (0.42 - 1.6)
6-≤10	2363 (43)	1.7 (0.51 - 2.8)	0.31 (0.20 - 0.48)	2454 (35)	0.67 (-0.13 -1.5)	0.43 (0.21 - 0.9)
> 10	1545 (28)	-1.3 (-2.6 - -0.10)	0.27 (0.15 - 0.47)	1609 (23)	-2.4 (-3.3 --1.5)	0.76 (0.40 - 1.5)
Area [c]	3583 (66)					
Urban	1851 (34)		1	4911 (70)		1
Rural		-4.0 (-4.8 - -3.2)	2.7 (1.8 - 4.0)	2155 (30)	-3.7 (-4.4 --3.0)	2.0 (1.3 - 3.1)
Latitude [d]						
North	2861 (53)		1	3402 (48)		1
South	2573 (47)	-1.5 (-2.3 - -0.74)	1.8 (1.2 - 2.6)	3664 (52)	2.6 (2.0 -3.2)	1.3 (0.83 - 2.0)
Current tobacco consumption						
No	2804 (52)		1	6553 (93)		1
Yes	2630 (48)	1.1 (0.36 - 1.9)	1.3 (0.91 - 1.9)	513 (7)	-1.9 (-3.1 --0.73)	1.5 (0.87 - 2.7)
Alcohol consumption ever						
No	3035 (56)		1	7059 (100)		1

Yes	2399 (44)	-0.71 (-1.5 - 0.06)	1.6 (1.08 - 2.3)	7 (0)	-9.3 (-19 -0.4)	1.0 (1.0 - 1.0)
Vegetarian						
No	3576 (66)		1	4396 (62)		1
Yes	1858 (34)	0.65 (-0.18 - 1.5)	0.61 (0.41 - 0.90)	2670 (38)	-2.1 (-2.7 --1.5)	0.70 (0.44 - 1.1)
Body mass index (kg/m2) [e]						
Underweight (≤18.5)	2888 (56)		1	2991 (44)		1
Normal (>18.5 - ≤25)	812 (16)	4.0 (2.9 - 5.2)	0.69 (0.42 - 1.1)	764 (11)	1.6 (0.57 -2.6)	1.1 (0.57 - 2.0)
Overweight (>25 - ≤30)	1209 (23)	-1.7 (-2.7 - -0.73)	0.71 (0.42 - 1.2)	2104 (31)	-0.11 (-0.84 -0.62)	0.67 (0.38 - 1.2)
Obese (>30)	243 (5)	-0.71 (-2.6 - 1.2)	0.36 (0.09 - 1.5)	907 (13)	-0.64 (-1.6 -0.33)	0.55 (0.23 - 1.3)
Fat free mass (kg/m2) [e]						
1st tertile (≤37)	361 (8)		1	3833 (58)		1
2nd tertile (>37 - <45)	1351 (28)	-0.42 (-2.1 - 1.2)	0.78 (0.44 - 1.4)	2535 (39)	-1.4 (-2.0 --0.74)	0.67 (0.38 - 1.2)
3rd tertile (≤45)	3093 (64)	-3.7 (-5.3 - -2.2)	0.50 (0.28 - 0.90)	208 (3)	-1.4 (-3.2 -0.45)	0.58 (0.08 - 4.2)

[a] Adjusted for age; [b] Not adjusted for age; [c] Urban areas include Delhi, Chennai and Sonipat district. Rural areas include Sonipat, Vishakhapatnam and Faridabad districts; [d] North areas include Delhi, Sonipat and Faridabad district. South areas include Chennai and Vishakhapatnam districts; [e] Variables with missing values.

For peer review only - http://bmjopen.bmj.com/site/about/guidelines.xhtml

Table S3. Multiple regression analysis of sociodemographic characteristics associated with eGFR and eGFR<60 including study participants with proteinuria (but without diabetes or hypertension), n=12533

	**eGFR**	**eGFR<60**
**Variable**	**Coefficient (95%CI)[*]**	**OR (95%CI)[*]**
Area [¥]		
Urban		1
Rural	-4.6 (-5.1 - -4.0)	1.9 (1.4 - 2.7)
Latitude [‡]		
North		1
South	0.29 (-0.21 - 0.78)	1.3 (0.98 - 1.8)
Education (number of years)		
0		1
5	0.83 (0 - 1.7)	0.55 (0.35 - 0.87)
10	0.04 (-0.64 - 0.72)	0.51 (0.35 - 0.76)
> 10	-3.8 (-4.6 - -3.0)	0.66 (0.4 - 1.1)
Alcohol consumption ever		
No		1
Yes	-0.78 (-1.5 - -0.05)	1.2 (0.85 - 1.8)
Sex		
Female		1
Male	-2.9 (-3.5 - -2.3)	1.4 (0.96 - 2.0)
Age	-0.91 (-0.93 - -0.89)	1.1 (1.1 - 1.1)

[a]Variables mutually adjusted, [b] Urban areas include Delhi, Chennai and Sonipat district. Rural areas include Sonipat, Vishakhapatnam and Faridabad districts; [c] North areas include Delhi, Sonipat and Faridabad district. South areas include Chennai and Vishakhapatnam districts.

For peer review only - http://bmjopen.bmj.com/site/about/guidelines.xhtml

Table S4. Multiple regression analysis of sociodemographic characteristics associated with eGFR and eGFR<60 including plasma fasting glucose, HbA1c and systolic blood pressure

	**eGFR**	**eGFR<60**
**Variable**	**Coefficient (95%CI)***	**OR (95%CI)***
Area ¥		
Urban		1
Rural	-4.9 (-5.5 - -4.4)	2.3 (1.6 – 3.2)
Latitude ‡		
North		1
South	0.23 (-0.26 - 0.72)	1.3 (0.95 - 1.8)
Education (number of years)		
0		1
5	1.0 (0.20 - 1.9)	0.49 (0.31 - 0.79)
10	0.19 (-0.49 - 0.87)	0.47 (0.31 - 0.71)
> 10	-3.5 (-4.3 - -2.8)	0.62 (0.40 - 1.0)
Alcohol consumption ever		
No		1
Yes	-0.72 (-1.4 - -0.01)	1.3 (0.90 - 1.9)
Sex		
Female		1
Male	-2.7 (-3.3 - -2.1)	1.5 (0.01 – 2.1)
Age	-0.89 (-0.92 - -0.87)	1.1 (1.1 – 1.1)
Systolic blood pressure (mm Hg)	-0.06 (-0.08 - -0.04)	1.0 (0.99 – 1.0)
Hb1Ac (%)	0.03 (-0.56 - 0.62)	1.9 (1.3 – 2.8)
Fasting plasma glucose (mg/dl)	-0.06 (-0.84 - -0.04)	1.0 (1.0 – 1.0)

[a] Variables mutually adjusted, [b] Urban areas include Delhi, Chennai and Sonipat district. Rural areas include Sonipat, Vishakhapatnam and Faridabad districts; [c] North areas include Delhi, Sonipat and Faridabad district. South areas include Chennai and Vishakhapatnam districts.

For peer review only - http://bmjopen.bmj.com/site/about/guidelines.xhtml

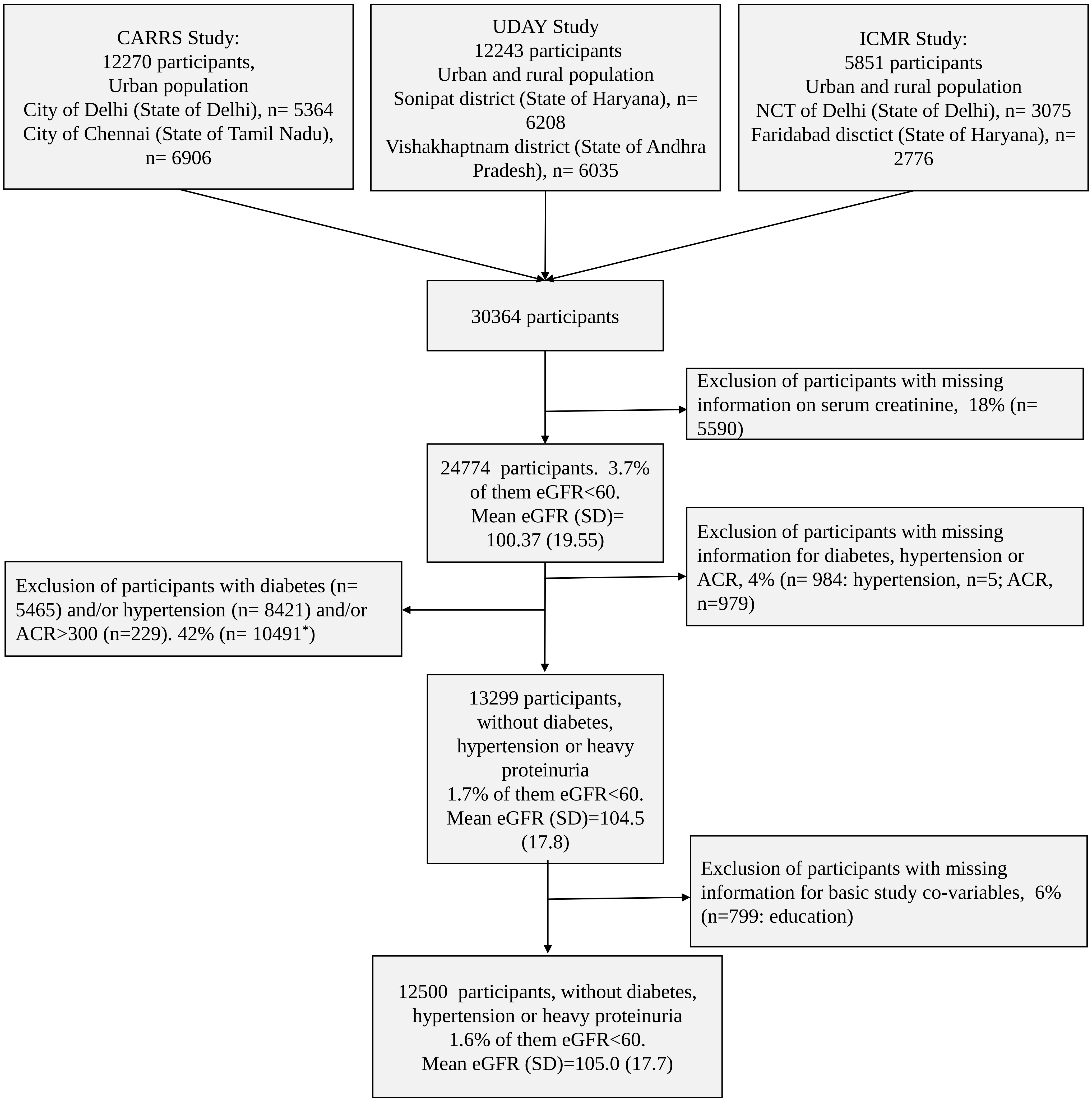

* 2353 participants with diabetes only; 5185 participants with hypertension only; 35 participants with ACR>30 only; 2724 participants with diabetes, and hypertension; 35 participants with diabetes and ACR>30; 47 participants with hypertension and ACR>30; 112 participants with diabetes, hypertension and ACR>30.

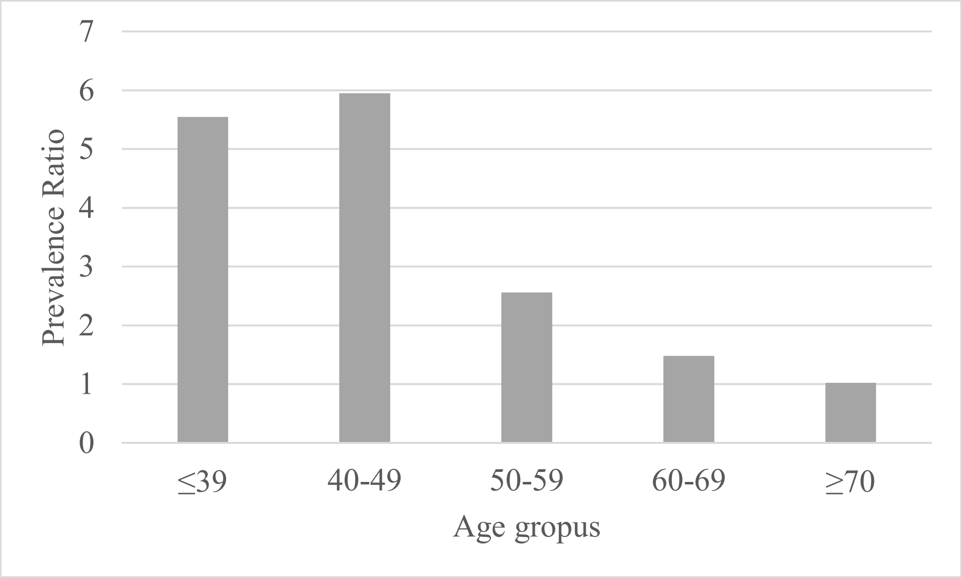

For peer review only

Supplementary material

Prevalence of and risk factors for chronic kidney disease of unknown aetiology in India: secondary data analysis of three population-based cross-sectional studies

Cristina O'Callaghan-Gordo, Roopa Shivashankar , Shuchi Anand, Shreeparna Ghosh , Jason Glaser, Ruby Gupta, Kristina Jakobsson, Dimple Kondal, Anand Krishnan, Sailesh Mohan, Viswanathan Mohan, Dorothea Nitsch, Praveen PA, Nikhil Tandon, K.M. Venkat Narayan, Neil Pearce Ben Caplin, Dorairaj Prabakharan

Content

Table 1. Sociodemographic and anthropometric characteristics of overall study participants (prior to exclusion of population with diabetes, hypertension and proteinuria)

Table 2. Associations between sociodemographic and anthropometric characteristics and estimated glomerular filtration rate (eGFR) and eGFR<60 by sex

Table 3. Multiple regression analysis of sociodemographic and anthropometric characteristics associated with eGFR and eGFR<60 including study participants with proteinuria (but without diabetes or hypertension)

Table 4. Multiple regression analysis of sociodemographic and anthropometric characteristics associated with eGFR and eGFR<60 including fasting plasma glucose, HbA1c and systolic blood pressure

For peer review only

Table 1. Sociodemographic and anthropometric characteristics of overall study participants (prior to exclusion of population with diabetes, hypertension and proteinuria)

Variable	n (%)[*] n=12 500	eGFR		eGFR categories, n(%)[**]			
		mean (SD)	p-value[§]	≥90	90-60	<60	p-value[§§]
**Socio-demographic factors**							
Age (years)							
<39	9007 (36)	112.9 (14.9)	<0.001	8248 (92)	716 (8)	43 (0)	<0.001
40-49	6924 (28)	101.9 (14.8)		5617 (81)	1215 (18)	92 (1)	
50-59	4524 (18)	92.9 (15.2)		2997 (66)	1378 (30)	149 (3)	
60-69	3045 (12)	82.8 (17.1)		1410 (46)	1315 (43)	320 (11)	
>=70	1274 (5)	72.0 (17.3)		164 (13)	806 (63)	304 (24)	
Sex							
Female	13433 (54)	102.6 (19.5)		10404 (77)	2585 (19)	444 (3)	<0.001
Male	11341 (46)	97.7 (19.3)	0.33	8032 (71)	2845 (25)	464 (4)	
Education (number of years)							
0	4794 (19)	97.7 (20.2)	<0.001	3458 (72)	1075 (22)	261 (5)	<0.001
5	3194 (13)	101.7 (19.4)		2456 (77)	625 (20)	113 (4)	
10	8855 (36)	103.2 (18.9)		6995 (79)	1620 (18)	240 (3)	
> 10	6358 (26)	100.0 (19.2)		4638 (73)	1538 (24)	182 (3)	
Missing data	1573 (6)			889 (57)	572 (36)	112 (7)	
Area [e]							
Urban	17732 (72)	102 (19.5)	0.03	13577 (77)	3602 (20)	553 (3)	<0.001
Rural	7042 (28)	96.3 (19.1)		4859 (69)	1828 (26)	355 (5)	
Latitude [f]							
North	13570 (55)	98.1 (19.1)	<0.001	9599 (71)	3439 (25)	532 (4)	<0.001
South	11204 (45)	103.1 (19.7)		8837 (79)	1991 (18)	376 (3)	
**Life-style factors**							
Current smoking							
No	18402 (74)	101.5 (19.6)	0.01	13920 (76)	3838 (21)	644 (3)	<0.001
Yes	6372 (26)	97.1 (19.1)		4516 (71)	1592 (25)	264 (4)	
Alcohol consumption ever							
No	19588 (79)	100.9 (19.6)	0.01	14671 (75)	4203 (21)	714 (4)	0.01
Yes	5186 (21)	98.5 (19.1)		3765 (73)	1227 (24)	194 (4)	
Vegetarian							
No	15043 (61)	102.7 (19.7)	<0.001	11721 (78)	2835 (19)	487 (3)	<0.001
Yes	9731 (39)	96.8 (18.9)		6715 (69)	2595 (27)	421 (4)	
**Biological factors**							
Body mass index (kg/m^2)							
Underweight (≤18.5)	10297 (42)	100.1 (19.6)	<0.001	7626 (74)	2284 (22)	387 (4)	0.01
Normal (>18.5 - ≤25)	2403 (10)	101.58 (20.5)		1838 (76)	471 (20)	94 (4)	
Overweight (>25 - ≤30)	7221 (29)	99.9 (18.8)		5309 (74)	1680 (23)	232 (3)	
Obese (>30)	3286 (13)	99.3 (19.2)		2392 (73)	766 (23)	128 (4)	
Missing data	1567 (6)			1271 (81)	229 (15)	67 (4)	
Fat free mass (kg/m^2)							
1st tertile (≤37)	7141 (29)	101.9 (20.1)	<0.001	5481 (77)	1381 (19)	279 (4)	<0.001

2nd tertile (>37 - <45)	7141 (29)	101.3 (19.1)	5419 (76)	1487 (21)	235 (3)
3rd tertile (≤45)	7141 (29)	98.3 (18.6)	5110 (72)	1797 (25)	234 (3)
Missing data	3351 (14)		2426 (72)	765 (23)	160 (5)

[a] Percentages in columns; [b] percentages in rows; [c] Bartlett's test for equal variance; [d] Chi-square test; [e] Urban areas include Delhi, Chennai and Sonipat district. Rural areas include Sonipat, Vishakhapatnam and Faridabad districts; [f] North areas include Delhi, Sonipat and Faridabad district. South areas include Chennai and Vishakhapatnam districts.

For peer review only

Table 2. Associations between sociodemographic and anthropometric characteristics and estimated glomerular filtration rate (eGFR) and eGFR<60 by sex

	Men, n=5 434			Women, n=7 066		
**Variable**		**eGFR**	**eGFR<60**		**eGFR**	**eGFR<60**
	**n (%)**	**Coefficient (95%CI) [a]**	**OR (95% CI) [a]**	**n (%)**	**Coefficient (95%CI) [a]**	**OR (95% CI) [a]**
Age (years) [b]						
<39	2335 (43)			3786 (54)		
40-49	1568 (29)	-9.0 (-10 - -8.0)	2.4 (1.2 - 4.6)	1908 (27)	-12 (-13 --12)	4.5 (1.9 - 10)
50-59	843 (16)	-17 (-18 - -16)	3.8 (1.9 - 7.7)	863 (12)	-21 (-22 --20)	12 (5.2 - 27)
60-69	479 (9)	-25 (-27 - -24)	13 (7.0 - 24)	414 (6)	-30 (-31 --29)	33 (15 - 73)
>=70	209 (4)	-34 (-36 - -32)	31 (16 - 59)	95 (1)	-35 (-38 --32)	43 (16 - 118)
Education (number of completed years)						
0	823 (15)		1	1997 (28)		1
≤5	703 (13)	3.3 (1.8 - 4.7)	0.24 (0.13 - 0.46)	1006 (14)	0.73 (-0.27 -1.7)	0.81 (0.42 - 1.6)
6-≤10	2363 (43)	1.7 (0.51 - 2.8)	0.31 (0.20 - 0.48)	2454 (35)	0.67 (-0.13 -1.5)	0.43 (0.21 - 0.9)
> 10	1545 (28)	-1.3 (-2.6 - -0.10)	0.27 (0.15 - 0.47)	1609 (23)	-2.4 (-3.3 --1.5)	0.76 (0.40 - 1.5)
Area [c]	3583 (66)					
Urban	1851 (34)		1	4911 (70)		1
Rural		-4.0 (-4.8 - -3.2)	2.7 (1.8 - 4.0)	2155 (30)	-3.7 (-4.4 --3.0)	2.0 (1.3 - 3.1)
Latitude [d]						
North	2861 (53)		1	3402 (48)		1
South	2573 (47)	-1.5 (-2.3 - -0.74)	1.8 (1.2 - 2.6)	3664 (52)	2.6 (2.0 -3.2)	1.3 (0.83 - 2.0)
Current tobacco consumption						
No	2804 (52)		1	6553 (93)		1
Yes	2630 (48)	1.1 (0.36 - 1.9)	1.3 (0.91 - 1.9)	513 (7)	-1.9 (-3.1 --0.73)	1.5 (0.87 - 2.7)
Alcohol consumption ever						
No	3035 (56)		1	7059 (100)		1

Yes	2399 (44)	-0.71 (-1.5 - 0.06)	1.6 (1.08 - 2.3)	7 (0)	-9.3 (-19 -0.4)	1.0 (1.0 - 1.0)
Vegetarian						
No	3576 (66)		1	4396 (62)		1
Yes	1858 (34)	0.65 (-0.18 - 1.5)	0.61 (0.41 - 0.90)	2670 (38)	-2.1 (-2.7 --1.5)	0.70 (0.44 - 1.1)
Body mass index (kg/m2) [e]						
Underweight (≤18.5)	2888 (56)		1	2991 (44)		1
Normal (>18.5 - ≤25)	812 (16)	4.0 (2.9 - 5.2)	0.69 (0.42 - 1.1)	764 (11)	1.6 (0.57 -2.6)	1.1 (0.57 - 2.0)
Overweight (>25 - ≤30)	1209 (23)	-1.7 (-2.7 - -0.73)	0.71 (0.42 - 1.2)	2104 (31)	-0.11 (-0.84 -0.62)	0.67 (0.38 - 1.2)
Obese (>30)	243 (5)	-0.71 (-2.6 - 1.2)	0.36 (0.09 - 1.5)	907 (13)	-0.64 (-1.6 -0.33)	0.55 (0.23 - 1.3)
Fat free mass (kg/m2) [e]						
1st tertile (≤37)	361 (8)		1	3833 (58)		1
2nd tertile (>37 - <45)	1351 (28)	-0.42 (-2.1 - 1.2)	0.78 (0.44 - 1.4)	2535 (39)	-1.4 (-2.0 --0.74)	0.67 (0.38 - 1.2)
3rd tertile (≤45)	3093 (64)	-3.7 (-5.3 - -2.2)	0.50 (0.28 - 0.90)	208 (3)	-1.4 (-3.2 -0.45)	0.58 (0.08 - 4.2)

[a] Adjusted for age; [b] Not adjusted for age; [c] Urban areas include Delhi, Chennai and Sonipat district. Rural areas include Sonipat, Vishakhapatnam and Faridabad districts; [d] North areas include Delhi, Sonipat and Faridabad district. South areas include Chennai and Vishakhapatnam districts; [e] Variables with missing values.

For peer review only - http://bmjopen.bmj.com/site/about/guidelines.xhtml

Table 3. Multiple regression analysis of sociodemographic characteristics associated with eGFR and eGFR<60 including study participants with proteinuria (but without diabetes or hypertension), n=12533

	eGFR	eGFR<60
**Variable**	**Coefficient (95%CI)***	**OR (95%CI)***
Area ¥		
Urban		1
Rural	-4.6 (-5.1 - -4.0)	1.9 (1.4 - 2.7)
Latitude ‡		
North		1
South	0.29 (-0.21 - 0.78)	1.3 (0.98 - 1.8)
Education (number of years)		
0		1
5	0.83 (0 - 1.7)	0.55 (0.35 - 0.87)
10	0.04 (-0.64 - 0.72)	0.51 (0.35 - 0.76)
> 10	-3.8 (-4.6 - -3.0)	0.66 (0.4 - 1.1)
Alcohol consumption ever		
No		1
Yes	-0.78 (-1.5 - -0.05)	1.2 (0.85 - 1.8)
Sex		
Female		1
Male	-2.9 (-3.5 - -2.3)	1.4 (0.96 - 2.0)
Age	-0.91 (-0.93 - -0.89)	1.1 (1.1 - 1.1)

[a]Variables mutually adjusted, [b] Urban areas include Delhi, Chennai and Sonipat district. Rural areas include Sonipat, Vishakhapatnam and Faridabad districts; [c] North areas include Delhi, Sonipat and Faridabad district. South areas include Chennai and Vishakhapatnam districts.

.

view only

Table 4. Multiple regression analysis of sociodemographic characteristics associated with eGFR and eGFR<60 including plasma fasting glucose, HbA1c and systolic blood pressure

	**eGFR**	**eGFR<60**
**Variable**	**Coefficient (95%CI)[*]**	**OR (95%CI)[*]**
Area [¥]		
Urban		1
Rural	-4.9 (-5.5 - -4.4)	2.3 (1.6 – 3.2)
Latitude [‡]		
North		1
South	0.23 (-0.26 - 0.72)	1.3 (0.95 - 1.8)
Education (number of years)		
0		1
5	1.0 (0.20 - 1.9)	0.49 (0.31 - 0.79)
10	0.19 (-0.49 - 0.87)	0.47 (0.31 - 0.71)
> 10	-3.5 (-4.3 - -2.8)	0.62 (0.40 - 1.0)
Alcohol consumption ever		
No		1
Yes	-0.72 (-1.4 - -0.01)	1.3 (0.90 - 1.9)
Sex		
Female		1
Male	-2.7 (-3.3 - -2.1)	1.5 (0.01 – 2.1)
Age	-0.89 (-0.92 - -0.87)	1.1 (1.1 – 1.1)
Systolic blood pressure (mm Hg)	-0.06 (-0.08 - -0.04)	1.0 (0.99 – 1.0)
Hb1Ac (%)	0.03 (-0.56 - 0.62)	1.9 (1.3 – 2.8)
Fasting plasma glucose (mg/dl)	-0.06 (-0.84 - -0.04)	1.0 (1.0 – 1.0)

[a] Variables mutually adjusted, [b] Urban areas include Delhi, Chennai and Sonipat district. Rural areas include Sonipat, Vishakhapatnam and Faridabad districts; [c] North areas include Delhi, Sonipat and Faridabad district. South areas include Chennai and Vishakhapatnam districts.

BMJ Open

Prevalence of and risk factors for chronic kidney disease of unknown aetiology in India: secondary data analysis of three population-based cross-sectional studies

Journal:	*BMJ Open*
Manuscript ID	bmjopen-2018-023353.R1
Article Type:	Research
Date Submitted by the Author:	17-Aug-2018
Complete List of Authors:	O'Callaghan Gordo, Cristina; Instituto de Salud Global Barcelona, Campus Mar<br>Shivashankar, Roopa; Public Health Foundation of India,<br>Anand, Shuchi; Stanford Hospital and Clinics,<br>Ghosh, Shreeparna; Public Health Foundation of India,<br>Glaser, Jason; La Isla Foundation; London School of Hygiene and Tropical Medicine, 3Department of Non-communicable Disease Epidemiology<br>Gupta, Ruby; Publichealth Foundation of India<br>Jakobsson , Kristina ; Lunds Universitet Arbets- och miljomedicin<br>Kondal, Dimple; Publichealth Foundation of India<br>Krishnan , Anand ; All India Institute of Medical Sciences Centre for Community Medicine<br>Mohan, Sailesh; Public Health Foundation of India,<br>Mohan, V; Madras Diabetes Research Foundation<br>Nitsch, Dorothea; LSHTM<br>PA , Praveen ; All India Institute of Medical Sciences, Department of Endocrinology and Metabolism<br>Tandon, Nikhil; All India Institute of Medical Sciences,<br>Narayan, K; Emory University School of Public Health, Global Health<br>Pearce, Neil; London School of Hygiene and Tropical Medicine<br>Caplin, Ben; University College London Medical School, Centre for Nephrology,<br>Prabhakaran, Dorairaj; Public Health Foundation of India, Centre for Control of Chronic Conditions and Injuries
**Primary Subject Heading**:	Epidemiology
Secondary Subject Heading:	Global health
Keywords:	EPIDEMIOLOGY, NEPHROLOGY, Chronic renal failure < NEPHROLOGY, PUBLIC HEALTH

TITLE PAGE

Title: Prevalence of and risk factors for chronic kidney disease of unknown aetiology in India: secondary data analysis of three population-based cross-sectional studies

Authors names and affiliations:

Cristina O'Callaghan-Gordo[1,2,3,4*]; Roopa Shivashankar[5, 6*]; Shuchi Anand [7]; Shreeparna Ghosh[5]; Jason Glaser[4, 8]; Ruby Gupta[5]; Kristina Jakobsson[9,10]; Dimple Kondal[5, 6]; Anand Krishnan[11]; Sailesh Mohan[5]; Viswanathan Mohan[12, 13]; , Dorothea Nitsch[14]; Praveen PA[6, 15]; Nikhil Tandon[15]; K.M. Narayan[16]; Neil Pearce[4, 17]; Ben Caplin[18**]; Dorairaj Prabhakaran[5, 6**].

[1] ISGlobal, Barcelona, Spain; [2] Universitat Pompeu Fabra (UPF), Barcelona, Spain; [3] CIBER Epidemiología y Salud Pública (CIBERESP), Madrid, Spain; [4] Department of Medical Statistics, London School of Hygiene and Tropical Medicine, London, UK; [5] Public Health Foundation of India (PHFI), New Delhi, India; [6] Centre for Control of Chronic Conditions (4Cs), New Delhi, India; [7] Stanford University School of Medicine; [8] La Isla Network; [9] Occupational and Environmental Medicine, Sahlgrenska Academy, Gothenburg University, Sweden; [10] Occupational and Environmental Medicine, Lund University, Sweden; [11] Centre for Community Medicine, All India Institute of Medical Sciences, New Delhi.; [12] Diabetes Research, Madras Diabetes Research Foundation, Chennai, India; [13] Dr. Mohan's Diabetes Specialities Centre, Chennai, India; [14] Department of Epidemiology and Population Health, London School of Hygiene and Tropical Medicine, , London, UK; [15] Department of Endocrinology and Metabolism, All India Institute of Medical Sciences, New Delhi, India; [16] Emory Global Diabetes Research Center, Rollins School of Public Health, Emory University, Atlanta, GA, USA; [17] Centre for Global NCDs, London School of Hygiene and Tropical Medicine, London, UK; [18] Centre for Nephrology, University College London Medical School, London, UK.

[*] Joint first authors; [**] Joint last authors

Corresponding author's name and email address:

Cristina O'Callaghan-Gordo, **cristina.ocallaghan@isglobal.org**

Word count: 3919

For peer review only - http://bmjopen.bmj.com/site/about/guidelines.xhtml

ABSTRACT

Objectives: To assess whether chronic kidney disease of unknown aetiology (CKDu) is present in India and to identify risk factors for it using population-based data and standardised methods.

Design: Secondary data analysis of three population-based cross-sectional studies conducted between 2010-2014.

Setting: Urban and rural areas of Northern India (states of Delhi and Haryana) and Southern India (states of Tamil Nadu and Andhra Pradesh)

Participants: 12,500 individuals without diabetes, hypertension or heavy proteinuria

Outcome measures: Mean estimated the glomerular filtration rate (eGFR) and the prevalence of eGFR below 60ml/min per 1.73m^2 (eGFR<60) in individuals without diabetes, hypertension or heavy proteinuria (proxy definition of CKDu).

Results: The mean eGFR was 105.0±17.8 ml/min per 1.73m2. The prevalence of eGFR<60 was 1.6% (95%CI=1.4, 1.7), but this figure varied markedly between areas, being highest in rural areas of Southern Indian [4.8% (3.8, 5.8)]. In Northern India, older age was the only risk factor associated with lower mean eGFR and eGFR<60 [regression coefficient (95%CI)=-0.94 (0.97, 0.91); OR (95%CI)=1.10 (1.08, 1.11)]. In Southern India, risk factors for lower mean eGFR and eGFR<60 respectively were residence in a rural area [-7.78 (-8.69, -6.86); 4.95 (2.61, 9.39)], older age [-0.90 (-0.93, -0.86); 1.06 (1.04, 1.08)] and less education [-0.94 (-1.32, -0.56); 0.67 (0.50, 0.90) for each five years at school].

Conclusions: CKDu is present in India and is not confined to Central America and Sri Lanka. Identified risk factors are consistent with risk factors previously reported for CKDu in Central America and Sri Lanka.

KEYWORDS

Epidemiology; Chronic kidney disease; Chronic kidney disease of unknown aetiology; India; Rural population

ARTICLE SUMMARY

Strengths and limitations of this study

- The use of a random selection of population-based participants allows the estimation of CKDu prevalence in the general population.

For peer review only - http://bmjopen.bmj.com/site/about/guidelines.xhtml

- A large sample size including participants from different areas of India (urban and rural, and Northern and Southern India) increases the representativeness of our results.
- The use of standardized definitions of CKDu facilitates international comparisons of CKDu prevalence and risk factors.
- The prevalence of eGFR<60 observed in this study is likely to be underestimated; however, this is unlikely to have biased the internal comparisons conducted in this study.

FUNDING

This work was supported in part by grant MR/P02386X/1 from the United Kingdom Medical Research Council under the Global Challenges Research Fund. It was also supported by grants from the Colt Foundation and the La Isla Foundation. The CARRS study was funded with federal funds from the National Heart, Lung, and Blood Institute, National Institutes of Health, under Contract No. HHSN2682009900026C. UDAY study was funded by Eli Lilly Foundation. ICMR-CHD study was funded by the Indian Council Medical Research (ICMR). The Centre for Global NCDs is supported by the Wellcome Trust Institutional Strategic Support Fund (097834/Z/11/B). CO-G holds a Sara Borrell postdoctoral fellowship awarded from the Carlos III National Institute of Health, Spain (CD13/00072).

For peer review only - http://bmjopen.bmj.com/site/about/guidelines.xhtml

INTRODUCTION

High prevalence of CKDu has mainly been reported in the last decades amongst the working age populations of agricultural communities of tropical/subtropical regions, specifically in Central America and Sri Lanka [1–3]. In Nicaragua and El Salvador, the estimated prevalence of estimated glomerular filtration rate (eGFR; the clinical measure of kidney function) below 60ml/min per 1.73m^2 (eGFR<60), in the absence of diabetes and hypertension, was 10-20% [4–6]. It has been suggested that CKDu may also be highly prevalent in other low and middle income countries (LMICs), including India [7–11]. However, it is not clear in which other regions of the world CKDu occurs, whether the underlying aetiology is the same in different regions and what the risk factors are. Currently, there is no consensus but factors such as heat stress, strenuous work, climatic conditions, agrochemical use, heavy metal exposure and infections have been suggested as risk factors [1,12–15].

Data on CKDu from India are scarce. The recent report of verbal autopsy data from India suggests CKD of all causes is a growing problem. However, it does not provide accurate population-based data on CKDu [16,17]. Existing reports indicate that CKDu may be common but it is difficult to be definite about this because of the absence of population-based studies using standardised and comparable methods. Data from the Indian CKD Registry, a hospital based registry of incident cases of CKD between 2006-2010, found that CKDu was the second commonest form of CKD after diabetic nephropathy [10]. However, this is restricted to referred cases and therefore may not be representative of the general population. There are also sporadic reports of high numbers of CKDu cases among agricultural communities of the South Eastern Indian states of Andhra Pradesh and Odisha (reviewed by Chatterejee [18] and Ganguli [19]). However, population-based data have not been reported for India.

We conducted a secondary analysis of representative sample surveys conducted in India between 2010-2014. Given the absence of a clear case definition for CKDu it is necessary to make a presumptive diagnosis based on measures/estimates of GFR in the absence of known risk factors for kidney disease. The overall aim of the current study was to use a methodology which is comparable to previous studies elsewhere in the world (particularly in Central America) to assess the extent to which reduced kidney function is a problem in India, and which areas and subpopulations are most affected. We therefore: (i) assessed the distribution eGFR and prevalence of eGFR below 60ml/min per 1.73m^2 (eGFR<60) in Indian populations restricted to those without known risk factors for CKD, i.e. diabetes, hypertension or heavy proteinuria; ii) compared these outcomes in

For peer review only - http://bmjopen.bmj.com/site/about/guidelines.xhtml

North and South India and in urban and rural populations; and (iii) identified the risk factors associated with these outcomes.

METHODS

Study population

We used cross-sectional data from three population-based studies conducted in India: the "Centre for Cardiometabolic Risk Reduction in South Asia" cohort study (CARRS study) [20], the "Implementing a Comprehensive Diabetes Prevention and Management Program" study (UDAY study) [21] and the "prevalence of coronary heart disease repeat survey" study funded by the Indian Council of Medical Research (ICMR-CHD study) [22]. Details on study design and selection of participants from the CARRS, UDAY and ICMR-CHD studies have been previously described [20–22] and are summarized in Table 1. Participants from CARRS, UDAY and ICMR-CHD studies provided informed consent prior to participation. The three studies obtained ethical clearance from the corresponding institutions.

For peer review only

For peer review only - http://bmjopen.bmj.com/site/about/guidelines.xhtml

Table 1. Design and methods of the three studies included in the current analysis

<table>
<tr><th></th><th colspan="2">CARRS</th><th colspan="4">UDAY</th><th colspan="2">ICMR-CHD</th></tr>
<tr><td>Latitude (North/South)</td><td>North</td><td>South</td><td colspan="2">North</td><td colspan="2">South</td><td colspan="2">North</td></tr>
<tr><td>Residence (Urban/Rural)</td><td colspan="2">Urban</td><td>Urban</td><td>Rural</td><td>Urban</td><td>Rural</td><td>Urban</td><td>Rural</td></tr>
<tr><td>District (and State)</td><td>Delhi (state of Delhi)</td><td>Chennai (state of Tamil Nadu)</td><td colspan="2">Sonipat (state of Haryana)</td><td colspan="2">Vishakhapatnam (state of Andhra Pradesh)</td><td>National Capital Territory of Delhi (state of Delhi)</td><td>Faridabad (state of Haryana)</td></tr>
<tr><td>Household sampling</td><td colspan="2">Multistage cluster random (wards - census enumeration blocks - households)</td><td colspan="4">Multistage cluster random (Census Enumeration blocks (urban) or villages (rural) - households)</td><td>Multistage cluster random (wards - census enumeration blocks - households)</td><td>Simple cluster random (based on Health and Demographic Surveillance System)</td></tr>
<tr><td>Individual sampling</td><td colspan="2">1 man and 1 woman from each household (selected by Kish method, [23].) [b]</td><td colspan="4">1 man and 1 woman from each household (selected by Kish method, [23].) [b]</td><td colspan="2">All adults</td></tr>
<tr><td>Age groups included</td><td colspan="2">≥ 20</td><td colspan="4">≥ 30</td><td colspan="2">≥ 30</td></tr>
<tr><td>Exclusion criteria</td><td colspan="8">Pregnant, bedridden and participants who were unable to comprehend the questionnaires due cognitive deficiencies were excluded</td></tr>
<tr><td>Study period</td><td colspan="2">October 2010 - November 2011</td><td colspan="4">July 2014 - December 2014</td><td colspan="2">August 2010 - January 2012</td></tr>
<tr><td>Laboratory [a]</td><td>PHFI [c]</td><td>MDRF [d]</td><td colspan="4">PHFI [c]</td><td colspan="2">PHFI [c]</td></tr>
</table>

[a] Study laboratories participated in Randox International Quality Assurance Scheme (RIQAS) for clinical chemistry and HbA1c during the entire study periods. [b] In households where only eligible men or only eligible women were present, we selected just one adult. [c] Public Health Foundation of India; [d] Madras Diabetes Research Foundation

For peer review only

For peer review only - http://bmjopen.bmj.com/site/about/guidelines.xhtml

For the current analyses, we excluded participants with missing information on serum creatinine, as this variable was necessary to estimate eGFR. As the focus of our study was CKDu, we excluded participants with known risk factors for CKD (i.e. diabetes and hypertension) or evidence of primary glomerular disease (as assessed by heavy proteinuria) or with missing information for these risk factors. We also excluded participants with missing information on basic co-variables (education) for all the analyses conducted. A study flowchart is presented in Figure 1. We classified participants as having: diabetes, if plasma fasting glucose was ≥126 mg/dl, or glycated haemoglobin A1c (HbA1c) was ≥6.5%, or the participant self-reported diabetes; hypertension, if systolic blood pressure was ≥140 mm Hg, or diastolic blood pressure was ≥90 mm Hg, or the participant self-reported hypertension; and heavy proteinuria, if the albumin/creatinine ratio (ACR) in urine was ≥300 mg/g. We used the CKD-EPI equation to estimate GFR (eGFR) [24].

Data collection and laboratory analyses

Data collection was conducted between October 2010 and December 2014. All three studies used a standardized questionnaire to collect data on age, sex, completed years of education (0, ≤5, >5-≤ 10, >10), alcohol intake (ever, never) and dietary habits (vegetarian yes, no). Weight, height and body composition were measured using stadiometers (SECA 214 in the three studies) and electronic bioimpedance measuring instruments (Tanita BC 418 in CARRS and ICMR-CHD studies, and Tanita BC 601 in UDAY study). Body mass index (BMI, kg/m^2) was calculated and categorized (≤18.5: underweight; >18.5-≤25: normal weight; >25-≤30: overweight; >30: obese) and fat free mass was derived from bioelectric impedance analysis (BIA). In CARRS and ICMR-CHD studies, fat free mass (Kg) was directly measured as previously described [25], whereas in UDAY study, fat free mass was estimated from the percentage of total body fat. To estimate total fat free mass from the percentage of body fat, we calculated the amount of total body fat by multiplying the percentage of body fat by the weight of the participant, and from that value we estimated the amount of fat free mass by subtracting the weight of total body fat from the total weight of the participant. Blood pressure was measured using electronic sphygmomanometers (OMRON (HEM-7080) in CARRS and ICMR-CHD studies, and OMRON (HEM 7200) in UDAY study), as previously reported [20,26]. Stadiometers, electronic bioimpedance measuring instruments, and electronic sphygmomanometes were calibrated before each study, and no re-calibration was needed during the duration of different studies. A fasting venous blood sample was used to measure glucose levels, HbA1c and serum creatinine levels and urine sample to measure albuminuria and creatinuria [20]. Glucose levels were measured using hexokinase/kinetic methods, HbA1c using high-performance liquid chromatography, serum

For peer review only - http://bmjopen.bmj.com/site/about/guidelines.xhtml

creatinine using the rate-blanked and compensated kinetic Jaffe method, traceable to isotope dilution mass spectrometry, and albuminuria using immune turbidmetric method [20]. Samples from UDAY, ICMR-CHD, and samples from CARRS from Delhi were analysed at Public Health Foundation of India (PHFI) laboratory and samples from CARRS from Chennai were analysed at Madras Diabetes Research Foundation (MDRF) laboratory. Both PHFI and MDRF laboratories used the same methodologies and protocols to analyse the samples and participated in Randox International Quality Assurance Scheme (RIQAS) for clinical chemistry and HbA1c during the entire study periods. Data from the three studies were homogenized and merged in a single data set.

Statistical analyses

We reported mean eGFR and prevalence eGFR<60 according to different characteristics of the study populations. UDAY and CARRS studies did not involve fully random population samples (since sampling was based on households, with one participant per household) and the proportions of study participants with particular outcomes (e.g. eGFR<60), will not be exactly the same (but very similar) to what would have been obtained with genuine random population samples; thus in this paper we refer to the prevalence in the study participants, not overall population prevalence estimates. We used linear regression models to estimate the associations between potential risk factors and eGFR and logistic regression models to estimate the associations between potential risk factors and eGFR<60. We also repeated the analyses separately for males and females. Variables associated with eGFR in the basic analyses (adjusted for age and sex) were considered for the multiple regression analysis. In the final multiple regression model, we included all variables that were of a priori interest and/or had shown independent associations with eGFR. We then checked for multicollinearity for each variable in the multiple regression analyses in comparison with the basic analyses [27]. 6% had missing values for basic co-variables (i.e. education) and were excluded from the analysis. 4% and 11% of participants had missing values for BMI and for fat free mass respectively. These participants were included in the main analysis, but we excluded them to compare models non-adjusted and adjusted for these variables. We calculated prevalence ratios of eGFR<60 by age-group for rural and urban areas. Urban areas were defined as "all places with a municipality, corporation, cantonment board or notified town area committee, etc., and all other places which satisfied the following criteria: a minimum population of 5,000; at least 75 per cent of the male main working population engaged in non-agricultural pursuits; and a density of population of at least 400 persons per km^2", according to the 2011 Census of India definition [28]. Finally, we estimated potential interactions between urban (versus

For peer review only - http://bmjopen.bmj.com/site/about/guidelines.xhtml

rural) residence and latitude (Northern India (i.e. states of Delhi and Haryana) versus Southern India (states of Tamil Nadu and Andhra Pradesh). Classification of latitude was done in concordance with the classification of major geographical areas on India defined by the Indian Council of Medical Research [29], figure 1. We conducted all analyses using Stata version 14 (StataCorp, College Station, TX, USA).

Patient and Public Involvement

Patients were not involved in the design of this analysis.

RESULTS

Characteristics of study participants

12,500 people were eligible for the current analyses (Figure 2). Table 2 summarizes the socio-demographic and anthropometric characteristics of the 12,500 study participants included in this analysis (the same information including participants with known risk factors for CKD (n=24,774) in supplementary material Table S1). The mean (standard deviation (±SD)) age of participants was 41.5±11.6 years. 88% (4,805/5,434) of the male population was formally employed; 76% (5,346/7,066) of women worked on house duties (i.e. housewives). The mean BMI was 24±5.0 kg/m^2 and mean fat free mass was 42±15 kg/m^2. The mean fasting plasma glucose was 91.9±12.3 mg/dl and the mean HbA1c was 5.5±0.4 %. The mean systolic and diastolic blood pressures were 114±12 mm Hg and 74±9 mm Hg, respectively. The median (inter quartile range, IQR) albumin/creatinine ratio (ACR) was 2.4 (4.3) mg/g (after exclusion of those with ACR>300mg/g, n=1,208).

Mean eGFR and prevalence of eGFR<60

The mean eGFR was 105.0±17.8 ml/min per 1.73m^2. The mean eGFR was lower at increasing ages, in males, in inhabitants from rural areas and in those from Northern India, in participants with no formal education, and in participants who reported tobacco consumption, alcohol intake and being vegetarian (Table 2). We observed differences in mean eGFR depending on the area, being 104.5±17.6 in urban areas of Northern India, 100.3±16.2 in rural areas of Northern India, 110.9±15.7 in urban areas of Southern India and 97.4±19.8 in the rural area of Southern India.

The prevalence of eGFR<60 among the study population was 1.6% (95% confidence interval (95% CI)=1.4, 1.9). Seventeen per cent (95% CI=16, 17) of study participants had eGFR≥60-<90 ml/min per 1.73m^2 and 82%

For peer review only - http://bmjopen.bmj.com/site/about/guidelines.xhtml

[95% confidence interval (95% CI)=81, 82] had eGFR≥90 ml/min per 1.73m^2. The prevalences of different categories of eGFR differed by formal education, tobacco consumption, alcohol intake and vegetarianism (Table 2). Also, we observed marked differences in the prevalence of eGFR<60 depending on the area, being 1.4 % (95% CI=1.1, 1.8) in urban areas of Northern India, 1.9 (95% CI=1.4, 2.6) in rural areas of Northern India, 0.43% (95% CI =0.03, 0.07) in urban areas of Southern India and 4.8 % (95% CI= 3.9, 5.9) in the rural area of Southern India. The prevalence ratio of eGFR<60 for rural versus urban residence was higher for participants <50 years than for older groups (Figure 3).

For peer review only

Table 2. Sociodemographic and anthropometric characteristics of study participants (population without diabetes, hypertension or heavy proteinuria)

**Variable**	**n (%)** [a] **n=12,500**	**eGFR**	**eGFR categories, n(%)** [b]		
		**mean (SD)**	**≥90**	**90-60**	**<60**
**Socio-demographic**					
Age (years)					
<39	6121 (49)	113.8 (14.6)	5656 (92)	443 (7)	22 (0)
40-49	3476 (28)	102.5 (14.2)	2864 (82)	572 (16)	40 (1)
50-59	1706 (14)	93.9 (14.3)	1163 (68)	503 (29)	40 (2)
60-69	893 (7)	85.3 (16.2)	463 (52)	368 (41)	62 (7)
>=70	304 (2)	77.5 (15.1)	62 (20)	201 (66)	41 (13)
Sex					
Female	7066 (57)	107.9 (17.1)	6039 (85)	945 (13)	82 (1)
Male	5434 (43)	101.3 (17.9)	4169 (77)	1142 (21)	123 (2)
Education (number completed years)					
0	2820 (23)	100.7 (19.0)	2165 (77)	551 (20)	104 (4)
≤5	1709 (14)	105.9 (17.3)	1412 (83)	273 (16)	24 (1)
6-≤10	4817 (39)	107.2 (16.8)	4095 (85)	675 (14)	47 (1)
> 10	3154 (25)	105.0 (17.5)	2536 (80)	588 (19)	30 (1)
Area [c]					
Urban	8494 (68)	107.8 (16.1)	7247 (85)	1171 (14)	76 (1)
Rural	4006 (32)	99.0 (18.0)	2961 (74)	916 (23)	129 (3)
Latitude [d]					
North	6263 (50)	103.0 (17.2)	4967 (79)	1197 (19)	99 (2)
South	6237 (50)	107.0 (18.1)	5241 (84)	890 (14)	106 (2)
**Life-style factors**					
Current tobacco consumption					
No	9357 (75)	106.8 (17.3)	7836 (84)	1406 (15)	115 (1)
Yes	3143 (25)	99.8 (18.1)	2372 (75)	681 (22)	90 (3)
Alcohol consumption ever					
No	10094 (81)	105.9 (17.4)	8362 (83)	1589 (16)	143 (1)
Yes	2406 (19)	101.1 (18.5)	1846 (77)	498 (21)	62 (3)
Vegetarian					

No	7972 (64)	107.0 (18.0)	6690 (84)	1154 (14)	128 (2)
Yes	4528 (36)	101.6 (16.6)	3518 (78)	933 (21)	77 (2)
**Biological factors**					
Body mass index (kg/m^2)					
Underweight (≤18.5)	5879 (47)	104.2 (17.9)	4734 (81)	1029 (18)	116 (2)
Normal (>18.5 - ≤25)	1576 (13)	104.7 (19.3)	1283 (81)	257 (16)	36 (2)
Overweight (>25 - ≤30)	3313 (27)	105.0 (16.9)	2710 (82)	568 (17)	35 (1)
Obese (>30)	1150 (9)	105.5 (16.4)	948 (82)	194 (17)	8 (1)
Missing data	582 (5)		533 (92)	39 (7)	10 (2)
Fat free mass (kg)					
1st tertile (≤37)	3746 (30)	106.6 (18.1)	3146 (84)	532 (14)	68 (2)
2nd tertile (>37 - <45)	3801 (30)	105.9 (17.2)	3145 (83)	601 (16)	55 (1)
3rd tertile (≥45)	3834 (31)	102.1 (17.0)	2981 (78)	801 (21)	52 (1)
Missing data	1119 (9)		936 (84)	153 (14)	30 (3)

205 [a] Percentages in columns; [b] percentages in rows; [c] Urban areas include Delhi, Chennai and Sonipat district. Rural
206 areas include Sonipat, Vishakhapatnam and Faridabad districts; [d] North areas include Delhi, Sonipat and
207 Faridabad district. South areas include Chennai and Vishakhapatnam districts.

For peer review only - http://bmjopen.bmj.com/site/about/guidelines.xhtml

Risk factors for lower eGFR and eGFR<60

As expected, age was an important risk factor for reduced eGFR: eGFR was 9.30 ml/min per 1.73 m^2 (95%CI=-9.51, -9.09, model adjusted for sex) lower for each additional 10 years of age. Additionally, being male, living in a rural setting, and consuming alcohol were associated with decreased mean eGFR (Table 3). Similarly, the odds of eGFR<60 also increased with age [OR per 10 years, adjusted for sex (95%CI)=2.34 (2.12, 2.59)] and being male, living in a rural setting, living in Southern India and consuming alcohol were also associated with eGFR<60 (Table 3). In general, risk factors for decreased mean eGFR and for eGFR<60 were similar for men and women (supplementary material, Table S2), but few differences were observed. Regarding mean eGFR, living in Southern India was associated with decreased mean eGFR in men and with increased mean eGFR in women; tobacco consumption was associated with increased mean eGFR in men and with decreased mean eGFR in women; vegetarianism was associated with decreased mean eGFR in women but not in men; and being overweight was associated with decreased mean eGFR but in men but not in women. Regarding risk of eGFR<60, living in Southern India was associated with increased risk of eGFR<60 in men but not in women.

For peer review only - http://bmjopen.bmj.com/site/about/guidelines.xhtml

221 **Table 3**. Associations between sociodemographic and anthropometric characteristics and eGFR and eGFR<60

	eGFR	eGFR<60
Variable	Coefficient (95 CI) [a]	OR (95 CI) [a]
Age (years) [b]		
<39	0.00 (ref)	1.00 (ref)
40-49	-11.08 (-11.68, -10.47)	3.15 (1.87, 5.32)
50-59	-19.43 (-20.20, -18.65)	6.41 (3.80, 10.83)
60-69	-27.84 (-28.86, -26.82)	19.68 (12.01, 32.26)
>=70	-35.04 (-36.71, -33.37)	39.23 (22.87, 67.23)
Sex c		
Female	0.00 (ref)	1.00 (ref)
Male	-3.55 (- 4.05, -3.06)	1.33 (0.99, 1.78)
Education (number of completed years)		
0	0.00 (ref)	1.00 (ref)
≤5	1.92 (1.09, 2.76)	0.41 (0.26, 0.65)
6-≤10	1.27 (0.61, 1.93)	0.36 (0.25, 0.53)
> 10	-1.86 (-2.59, -1.14)	0.40 (0.26, 0.62)
Area [d]		
Urban	0.00 (ref)	1.00 (ref)
Rural	-3.84 (-4.37, -3.32)	2.39 (1.78, 3.22)
Latitude [e]		
North	0.00 (ref)	1.00 (ref)
South	0.86 (0.37, 1.35)	1.55 (1.16, 2.07)
Current tobacco consumption		
No	0.00 (ref)	1.00 (ref)
Yes	0.38 (-0.26, 1.02)	1.39 (1.01, 1.91)
Alcohol consumption ever		
No	0.00 (ref)	1.00 (ref)
Yes	-0.81 (-1.55, -0.08)	1.57 (1.09, 2.27)

Vegetarian		
No	0.00 (ref)	1.00 (ref)
Yes	-0.99 (-1.50, -0.47)	0.65 (0.48, 0.88)
Body mass index (kg/m2)		
Underweight (≤18.5)	2.96 (2.20, 3.73)	0.81 (0.55, 1.20)
Normal (>18.5 - ≤25)	0.00 (ref)	1.00 (ref)
Overweight (>25 - ≤30)	-0.75 (-1.34, -0.16)	0.68 (0.46, 1.01)
Obese (>30)	-0.71 (-1.59, 0.17)	0.47 (0.23, 0.98)
Fat free mass (kg)		
1st tertile (≤37)	0.00 (ref)	1.00 (ref)
2nd tertile (>37 - <45)	-0.91 (-1.54, -0.28)	0.69 (0.47, 1.03)
3rd tertile (≥45)	-3.90 (-4.77, -3.04)	0.49 (0.31, 0.80)

[a] Adjusted for age and sex; [b] Adjusted just for sex; [c] Adjusted just for age; [d] Urban areas include Delhi, Chennai and Sonipat district. Rural areas include Sonipat, Vishakhapatnam and Faridabad districts; [e] North areas include Delhi, Sonipat and Faridabad district. South areas include Chennai and Vishakhapatnam districts.

For peer review only - http://bmjopen.bmj.com/site/about/guidelines.xhtml

In the multiple regression analyses, decreased mean eGFR remained associated with older age, being male and living in a rural setting and alcohol consumption (Table 4). Risk of eGFR<60 remained associated with older age, being male and living in a rural setting and having no formal education remained associated with increased risk of eGFR<60 (Table 4). We adjusted all the multiple regression models for fat free mass and vegetarianism to assess the possibility that differences observed between urban and rural participants were due to differences in diet and/or body composition. These adjustments had little effect on the results (Table 4).

For peer review only

For peer review only - http://bmjopen.bmj.com/site/about/guidelines.xhtml

Table 4. Multiple regression analyses of sociodemographic characteristics associated with eGFR and eGFR<6).

	eGFR Coefficient (95% CI)			eGFR<60 OR (95% CI)		
Variable	Model 1[a]	Model 2[b]	Model 3[c]	Model 1[a]	Model 2[b]	Model 3[c]
Area [d]						
Urban	0.00 (ref)	0.00 (ref)	0.00 (ref)	1.00 (ref)	1.00 (ref)	1.00 (ref)
Rural	-4.57 (-5.13, -4.02)	-3.94 (-4.53, -3.36)	-4.10 (-4.70, -3.51)	1.99 (1.43, 2.76)	1.61 (1.12, 2.30)	1.65 (1.14, 2.37)
Latitude [e]						
North	0.00 (ref)	0.00 (ref)	0.00 (ref)	1.00 (ref)	1.00 (ref)	1.00 (ref)
South	0.31 (-0.18, 0.80)	-0.10 (-0.61, 0.41)	0.26 (-0.37, 0.89)	1.33 (0.98, 1.81)	1.60 (1.14, 2.32)	1.33 (0.86, 2.04)
Education (number of completed years)						
0	0.00 (ref)	0.00 (ref)	0.00 (ref)	1.00 (ref)	1.00 (ref)	1.00 (ref)
≤5	0.94 (0.01, 1.77)	1.16 (0.30, 2.02)	1.18 (0.32, 2.04)	0.50 (0.31, 0.80)	0.44 (0.26, 0.74)	0.45 (0.26, 0.75)
6-≤10	0.04 (-0.64, 0.72)	0.21 (-0.49, 0.91)	0.21 (-0.50, 0.92)	0.50 (0.34, 0.75)	0.38 (0.24, 0.60)	0.39 (0.25, 0.62)
> 10	-3.81 (-4.6, -3.0)	-3.81 (-4.60, -3.02)	-3.78 (-4.59, -2.97)	0.68 (0.42, 1.11)	0.61 (0.36, 1.03)	0.65 (0.38, 1.11)
Alcohol consumption ever						
No	0.00 (ref)	0.00 (ref)	0.00 (ref)	1.00 (ref)	1.00 (ref)	1.00 (ref)

Yes	-0.85 (-1.58, -0.12)	-0.69 (-1.47, 0.08)	-0.63 (-1.41, 0.15)	1.28 (0.88, 1.87)	1.18 (0.78, 1.79)	1.15 (0.76, 1.74)
Sex						
Female	0.00 (ref)	0.00 (ref)	0.00 (ref)	1.00 (ref)	1.00 (ref)	1.00 (ref)
Male	-2.85 (-3.44, -2.25)	-3.00 (-3.62, -2.38)	-2.52 (-3.18, -1.86)	1.39 (0.96, 2.01)	1.49 (1.00, 2.21)	1.50 (0.97, 2.31)
Age (per 10 years)	-9.10 (-9.32, -8.88)	-9.09 (-9.32, -8.86)	-9.15 (-9.38, -8.91)	2.21 (1.98, 2.47)	2.25 (2.00, 2.55)	2.27 (2.00, 2.57)
Fat free mass (kg)			-0.04 (-0.06, -0.02)			1.0 (0.98, 1.02)
Vegetarian						
No			0.00 (ref)			1.00 (ref)
Yes			0.66 (-0.03, 1.35)			0.74 (0.47, 1.18)

[a] Model 1 included the following variables: area, latitude, education, alcohol consumption, sex and age; n=12,500**;** [b] Model 2 included the same variables than model 1. Participants with missing information on fat free mass were excluded from the analysis, n=11,381; [c] Model 3 included the same variables than model 1 plus fat free mass and vegetarianism, n=11,381. [d] Urban areas include Delhi, Chennai and Sonipat district. Rural areas include Sonipat, Vishakhapatnam and Faridabad districts; [e] North areas include Delhi, Sonipat and Faridabad district. South areas include Chennai and Vishakhapatnam districts.

review only

For peer review only - http://bmjopen.bmj.com/site/about/guidelines.xhtml

We observed an interaction between the effects of latitude (North/South) and urban/rural residence in association with reduced eGFR (p-value for interaction<0.001). The mean eGFR was lower in rural settings in both Northern and Southern India (controlling for age, sex, education and alcohol intake). However, this decrease was much more marked in Southern India. In Northern India, rural residence, formal education (and duration) and age were the only other risk factor associated with reduced eGFR. In Southern India, being male was also a risk factor for reduced eGFR, whereas formal education was only a risk factor for reduced eGFR among those with more than 10 years of schooling (Table 5). We also observed an interaction between the effects of latitude (North/South) and urban/rural residence in association with eGFR<60 (p-value likelihood-ratio test for interaction<0.001). In Northern India, eGFR<60 was not associated with urban/rural residence, and older age was the only factor associated with eGFR<60. In Southern India, rural residence was the strongest risk factor for eGFR<60 but older age and lower years of formal education also increased the risk of eGFR<60 (Table 5).

For peer review only

For peer review only - http://bmjopen.bmj.com/site/about/guidelines.xhtml

Table 5. Multivariate analysis of sociodemographic characteristics associated with eGFR and with eGFR<60 according to latitude [a]

	eGFR (n=12,500)		eGFR<60(n=12,500)	
	North (n=6263) [a]	South (n= 6237) [b]	North (n=6263) [a]	South (n= 6237) [b]
Variables	Coefficient (95% CI)	Coefficient (95% CI)	OR (95% CI)	OR (95% CI)
Area [c]				
Urban	0.00 (ref)	0.00 (ref)	1.00 (ref)	1.00 (ref)
Rural	-1.42 (-2.15, -0.70)	-7.90 (-8.81, -7.00)	0.88 (0.57, 1.37)	4.68 (2.50, 8.77)
Education (number of completed years)				
0	0.00 (ref)	0.00 (ref)	1.00 (ref)	1.00 (ref) **
≤5	-1.32 (-2.58, -0.05)	1.05 (-0.06, 2.16)	1.16 (0.57, 2.35)	0.40 (0.20, 0.80)
6-≤10	-3.50 (-4.48, -2.52)	0.28 (-0.74, 1.30)	1.34 (0.74, 2.41)	0.35 (0.16, 0.74)
> 10	-6.93 (-7.97, -5.89)	-2.85 (-4.03, -1.67)	1.34 (0.69, 2.58)	0.61 (0.24, 1.57)
Alcohol consumption ever				
No	0.00 (ref)	0.00 (ref)	1.00 (ref)	1.00 (ref)
Yes	-0.54 (-1.55, 0.47)	-0.06 (-1.11, 0.99)	1.09 (0.62, 1.92)	1.36 (0.74, 2.17)
Sex				
Female	0.00 (ref)	0.00 (ref)	1.00 (ref)	1.00 (ref)
Male	-0.17 (-0.96, 0.63)	-5.40 (-6.29, -4.51)	0.97 (0.59, 1.59)	1.58 (0.91, 2.75)
Age (per 10 years)	-9.26 (-9.55, -8.97)	-8.96 (-9.28, -8.64)	2.51 (2.15, 2.93)	2.10 (1.77, 2.50)

** Likelihood ratio test for linear trend <0.05, OR (95% CI)=0.68 (0.51, 0.91). [a] North areas include Delhi, Sonipat and Faridabad district. [b] South areas include Chennai and Vishakhapatnam districts. [c] Urban areas include Delhi, Chennai and Sonipat district. Rural areas include Sonipat, Vishakhapatnam and Faridabad districts

For peer review only - http://bmjopen.bmj.com/site/about/guidelines.xhtml

Sensitivity analyses

We performed a sensitivity analysis including those with ACR>300 (but without hypertension or diabetes, n=33) as we were concerned that those with CKDu might develop proteinuria at more advanced CKD stages. However, this did not alter the mean eGFR (mean eGFR among the overall study population=105.0±17.8, mean eGFR in this sensitivity analysis =105.0±17.8), nor the estimated prevalence of eGFR<60 (prevalence among the overall study population=1.6%; prevalence in this sensitivity analysis =1.7%). The findings on risk factors were also similar to the findings from the primary analyses (supplementary material, Table S3).

Given concerns about potentially different thresholds to define diabetes and high blood pressure in different ethnic groups [30,31], we performed a further sensitivity analysis including fasting plasma glucose, HbA1c and systolic blood pressure in the multivariate model (even though there is evidence for both causation and reverse causation between these factors and CKD [32]). Systolic blood pressure and fasting plasma glucose were associated with reduced eGFR in this non diabetic population, but inclusion of these variables did not alter the coefficients for the associations with other risk factors observed in the primary analysis (supplementary material, Table S4). HbA1c was associated with eGFR<60 in this non diabetic population but inclusion of this variable did not alter the OR for other risk factors observed in the primary analysis (supplementary material, Table S4). Therefore, although the relationship between sub-clinical diabetes and impaired kidney function requires further prospective investigation, there is no evidence that the excess risk of low eGFR (i.e. lower mean eGFR and higher prevalence of eGFR<60) in rural Southern India is associated with either impaired fasting glucose or higher blood pressure.

DISCUSSION

We report the distribution of eGFR in people without diabetes, hypertension or heavy proteinuria and estimate the prevalence of CKDu in our study population, including participants from urban and rural settings. This is the first population-based evidence, using standardised methods, which indicates that CKDu is present in India and is not confined to Central America and Sri Lanka. We found that the rural population from Southern India (Vishakhapatnam district) had the highest risk of decreased eGFR (lower mean eGFR and higher prevalence of eGFR<60). Risk factors of decreased eGFR were different between Southern and Northern India. In Southern India, rural residence, older age and being male were risk

For peer review only - http://bmjopen.bmj.com/site/about/guidelines.xhtml

factors for both lower mean eGFR and eGFR<60; education was associated with decreased risk for eGFR<60 but not with lower mean eGFR. In Northern India, older age was the only risk factor for both lower mean eGFR and eGFR<60; rural residence and years of formal education were associated with lower mean eGFR but not with eGFR<60. In summary, in Southern India, older age, being male and rural residence were the main risk factors for decreased eGFR, whereas in Norther India older age was the main risk factors for decreased eGFR.

As in Central America, the risk of low eGFR was higher in rural settings than in urban settings. This is in concordance with a previous study from Hyderabad (India), that has provided evidence of a higher risk of low eGFR in a rural population compared to urban-migrant and urban population [33], and with various studies from other LMICs that have provided evidence of clusters of CKDu among the rural population [2,3]. Exposure to some of the suggested potential risk factors for CKDu such as agricultural work and agrochemical exposure, amongst others [34], may be greater in rural settings. Such exposures may also differ between Southern and Northern India, and potentially explain the differences observed between these areas. The associations between urban/rural residence and lower mean eGFR was much more marked in Southern India than in Northern India, and the associations between urban/rural residence and eGFR<60 was only observed in Southern India. The higher prevalence ratio (for eGFR<60) in the working age population compared to older age groups is consistent with the hypothesis that deceased in eGFR could be potentially explained by occupational exposures. The suggestive sex differences may also support this hypothesis. However, we did not have detailed data on occupation that allowed us to explore these associations in greater detail.

The higher risk of low eGFR in Southern India (Chennai and Vishakhapatnam districts) observed in our study is consistent with the clusters of CKDu cases previously reported in the Southern Indian states of Andhra Pradesh and Odisha [11,18,19]. Visakhapatnam district (state of Andhra Pradesh) and Chennai district (state of Tamil Nadu) have a similar climate than these areas where CKDu clusters have previously reported [35]. In these districts, mean temperatures range from 18 ºC to 37 ºC and rainfall occurs mainly between June and December [36]. On the other hand, sites from Norther India included in the study (Delhi (state of Delhi), Sonipat and Faridabad (Haryana state)), have a different climate. In these districts mean temperature ranges from 8 ºC to 39 ºC and precipitation occurs mainly between July

For peer review only - http://bmjopen.bmj.com/site/about/guidelines.xhtml

and August [36]. A previous study conducted in Costa Rica found a spatial correlation between rates of CKD mortality and temperature and rainfall [13].

About 5% of the rural population of Vishakhapatnam (Andra Pradesh, Southern India) without diabetes, hypertension or proteinuria had eGFR<60. This figure is almost as high as the prevalence observed in the USA (i.e. 6.7%) including people with diabetes, hypertension or proteinuria [37]. Moreover, the estimates of GFR in our study are likely to be underestimated. The CKD-EPI equation has been standardised for the white and Afro-American population [24], but its validity for other ethnic groups has been questioned [38,39]. Previous studies using CKD-EPI equation to estimate GFR in Indian populations reported mean eGFR values similar to the mean eGFR reported in our study (i.e. 104.9 ± 25.52 ml/min/1.73 m^2) [40]. However, two studies conducted among healthy kidney donors in India (population similar to those included in this analysis) have reported mean (measured) GFR between 81.4 and 95.5 ml/min per 1.73 m^2 [41,42], suggesting that the CKD-EPI equation substantially overestimates eGFR in the Indian population. Therefore, the prevalence of eGFR<60 observed in this study is likely to be substantially underestimated (although this is unlikely to have biased the internal comparisons, e.g. between urban and rural settings). The use of a conservative definition of the population susceptible to CKDu, may have also underestimated the prevalence of eGFR<60 in our study, as the population with diabetes, hypertension or glomerular disease may also have reduced eGFR due to other ('unknown') causes. To estimate the actual prevalence of reduced eGFR, future studies should include validated methods to estimate GFR in the Indian population. We were concerned that the validity of CKD-EPI among the Indian population may be also compromised by differences in muscular mass and meat consumption between population groups within India. We adjusted the analyses for fat free mass and vegetarianism, but this did not alter the results, suggesting no confounding effect by these variables.

Our study has at least three potential limitations. First, we only had one measure of eGFR, and therefore we could not differentiate acute kidney injury (AKI) from CKD. This is a common limitation in epidemiological studies, as it is challenging to obtain more than one measure of eGFR at least 3 months apart in large population-based investigations. Therefore, we may have misclassified some cases of AKI as reduced eGFR, and therefore overestimate the prevalence of this condition. Nevertheless, there is no a priori reason to think that potential misclassification was different according to the evaluated risks factors. Second, the three population-based studies included in this analysis used different sampling strategies.

For peer review only - http://bmjopen.bmj.com/site/about/guidelines.xhtml

CARRS and UDAY studies included only one man and one woman from all the eligible participants of selected households, whereas ICMR-CHD included all eligible adults from each selected household. This could have slightly biased our results (including our prevalence estimates) if risk factors potentially associated with CKDu were different between households inhabited only by a man and a women or by extended families. Third, information on other potential risk factors for CKDu, such as infections by leptospora or hantavirus infection, or use of nonsteroidal anti-inflammatory drugs (NSAIDs) was not available.

The main strengths of the study are the use of a random selection of population-based participants and a large sample size including participants from different areas of India (urban and rural, and Northern and Southern India). Moreover, we used the definitions proposed in DRGREE study [43], that aims to allow international comparisons of CKDu prevalence and help in the description of risk factors and in identifying the causes and mechanisms leading to CKDu.

In conclusion, our findings indicate that reduced eGFR, consistent with the definition of CKDu, is common in rural settings of Southern India (Vishakhapatnam district). This results support the hypothesis that the epidemic of CKDu, initially described in agricultural communities of Central America and Sri Lanka, may be common in other rural communities of tropical/subtropical countries. This has important implications for global health, since it indicates that CKDu may have a substantial public health burden globally that has been previously unrecognised. Population-based studies in other tropical/subtropical countries are required to assess the global patterns of burden of disease from CKDu [43].

AUTHOR CONTRIBUTIONS AND ACKNOWLEDGEMENTS

CO-G, BC, NP and DP designed the work; RS, SA, SG, RG, AK, SM, VM, PPA, NT, and KMN collected the data; CO-G and DK conducted the analysis of the data; CO-G, RS, SA, JG, KJ, DN, SM, KMN, NP, BC, and DP interpreted the data of the work. CO-G, RS, BC, and NP drafted the manuscript; RS, SA, SG, JG, RG, KJ, DK, AK, SM, VM, DN, PPA, NT, KMN, and DP revised the manuscript for important intellectual content, provided comments and suggested revisions. All authors approved the final version for publication.

We thank Manolis Kogevinas for his comments on the advanced version of the manuscript.

For peer review only

For peer review only - http://bmjopen.bmj.com/site/about/guidelines.xhtml

CONFLICTS OF INTERESTS

The authors declare that they have no competing interests

DATA SHARING STATEMENT

The datasets used and/or analysed during the current study are available from Public Health Foundation of India (PHFI) on reasonable request. Interested investigators should contact PHFI. Computing code can be obtained from the corresponding author.

For peer review only

For peer review only - http://bmjopen.bmj.com/site/about/guidelines.xhtml

REFERENCES

1 Wesseling C, Crowe J, Hogstedt C, *et al.* Mesoamerican Nephropathy: Report from the First International Research Workshop on MeN. Heredia, Costa Rica: 2013.

2 Correa-Rotter R, Wesseling C, Johnson RJ. CKD of unknown origin in Central America: the case for a Mesoamerican nephropathy. *Am J Kidney Dis* 2014;**63**:506–20. doi:10.1053/j.ajkd.2013.10.062

3 Jayatilake N, Mendis S, Maheepala P, *et al.* Chronic kidney disease of uncertain aetiology: Prevalence and causative factors in a developing country. *BMC Nephrol* 2013;**14**:1. doi:10.1186/1471-2369-14-180

4 Lebov JF, Valladares E, Pena R, *et al.* A population-based study of prevalence and risk factors of chronic kidney disease in Leon, Nicaragua. *Can J kidney Heal Dis* 2015;**2**:6. doi:10.1186/s40697-015-0041-1

5 Peraza S, Wesseling C, Aragon A, *et al.* Decreased kidney function among agricultural workers in El Salvador. *Am J Kidney Dis* 2012;**59**:531–40. doi:10.1053/j.ajkd.2011.11.039

6 Torres C, Aragón A, González M, *et al.* Decreased kidney function of unknown cause in Nicaragua: a community-based survey. *Am J Kidney Dis* 2010;**55**:485–96. doi:10.1053/j.ajkd.2009.12.012

7 Seck SM, Doupa D, Gueye L, *et al.* Prevalence of chronic kidney disease and associated factors in senegalese populations: a community-based study in saint-louis. *Nephrourol Mon* 2014;**6**:e19085. doi:10.5812/numonthly.19085

8 Barsoum RS. Burden of chronic kidney disease: North Africa. *Kidney Int Suppl* 2013;**3**:164–6. doi:10.1038/kisup.2013.5

9 El Minshawy O, Ghabrah T, El Bassuoni E. End-stage renal disease in Tabuk Area, Saudi Arabia: an epidemiological study. *Saudi J Kidney Dis Transpl* 2014;**25**:192–5.

10 Rajapurkar MM, John GT, Kirpalani AL, *et al.* What do we know about chronic kidney disease in India: First report of the Indian CKD registry. *BMC Nephrol* 2012;**13**. doi:10.1186/1471-2369-13-10

11 Reddy D V., Gunasekar A. Chronic kidney disease in two coastal districts of Andhra Pradesh, India: Role of drinking water. *Environ Geochem Health* 2013;**35**:439–54. doi:10.1007/s10653-012-9506-7

12 Jayasumana C, Paranagama P, Agampodi S, *et al.* Drinking well water and occupational exposure to Herbicides is associated with chronic kidney disease, in Padavi-Sripura, Sri Lanka -No section-. *Environ Heal A Glob Access Sci Source* 2015;**14**. doi:10.1186/1476-069X-14-6

13 Wesseling C, Van Wendel De Joode B, Crowe J, *et al.* Mesoamerican nephropathy: Geographical distribution and time trends of chronic kidney disease mortality between 1970 and 2012 in Costa Rica. *Occup Environ Med* 2015;**72**:714–21. doi:10.1136/oemed-2014-102799

14 Garcia-Garcia G, Jha V, World Kidney Day Steering Committee. Environmental and occupational factors in CKD. *Occup Environ Med* 2015;**72**:238. doi:10.1136/oemed-2015-102859

15 Robey RB. Cyclical dehydration-induced renal injury and Mesoamerican nephropathy: as sweet by any other name? *Kidney Int* 2014;**86**:226–9. doi:10.1038/ki.2014.47

16 Jha V, Modi G. Uncovering the rising kidney failure deaths in India. *Lancet Glob Heal* 2017;**5**:e14–5. doi:10.1016/S2214-109X(16)30299-6

17 Dare AJ, Fu SH, Patra J, *et al.* Renal failure deaths and their risk factors in India 2001–13: nationally representative estimates from the Million Death Study. *Lancet Glob Heal* 2017;**5**:e89–95. doi:10.1016/S2214-109X(16)30308-4

18 Chatterjee R. Occupational Hazard. *Science (80-)* 1026;**352**:24–7.

19 Ganguli A. Uddanam Nephropathy/Regional Nephropathy in India: Preliminary Findings and a Plea for Further Research. *Am J Kidney Dis* 2016;**68**:344–8. doi:10.1053/j.ajkd.2016.04.012

20 Nair M, Ali MK, Ajay VS, *et al.* CARRS Surveillance study: Design and methods to assess burdens from multiple perspectives. *BMC Public Health* 2012;**12**:1. doi:10.1186/1471-2458-12-

For peer review only

For peer review only - http://bmjopen.bmj.com/site/about/guidelines.xhtml

701

21 Mohan S, Jarhyan P, Ghosh S, *et al.* UDAY: Protocol of a Comprehensive Diabetes and Hypertension Prevention and Management Program in India. *BMJ open* 2018;**8**:e015919. doi:e015919. doi: 10.1136/bmjopen-2017-015919

22 Prabhakaran D, Roy A, Praveen PA, *et al.* 20-Year Trend of Cardiovascular Disease Risk Factors. *Glob Heart* Published Online First: 2017. doi:10.1016/j.gheart.2016.11.004

23 World Health Organization. STEPS Manual. 2015.

24 Levey AS, Stevens LA, Schmid CH, *et al.* A new equation to estimate glomerular filtration rate. *Ann Intern Med* 2009;**150**:604–12.

25 Patel SA, Deepa M, Shivashankar R, *et al.* Comparison of multiple obesity indices for cardiovascular disease risk classification in South Asian adults: The CARRS Study. *PLoS One* 2017;**12**:e0174251.

26 Anand S, Shivashankar R, Ali MK, *et al.* Prevalence of chronic kidney disease in two major Indian cities and projections for associated cardiovascular disease. *Kidney Int* 2015;**88**:178–85. doi:10.1038/ki.2015.58

27 Greenland S, Daniel R, Pearce N, *et al.* Outcome modelling strategies in epidemiology: traditional methods and basic alternatives. *Int J Epidemiol* 2016;:1–11. doi:10.1093/ije/dyw040

28 Census of India. 2011.http://censusindia.gov.in/ (accessed 1 Aug 2018).

29 Longvah T, Ananthan R, Bhaskarachary K, *et al.* Indian Food Composition tables. Hyderabad: 2017.

30 Herman WH. Do race and ethnicity impact hemoglobin A1c independent of glycemia? *J Diabetes Sci Technol* 2009;**3**:656–60. doi:10.1177/193229680900300406

31 Modesti PA, Reboldi G, Cappuccio FP, *et al.* Panethnic Differences in Blood Pressure in Europe: A Systematic Review and Meta-Analysis. *PLoS One* 2016;**11**:e0147601. doi:10.1371/journal.pone.0147601

32 Verhave JC, Hillege HL, Burgerhof JGM, *et al.* The association between atherosclerotic risk factors and renal function in the general population. *Kidney Int* 2005;**67**:1967–73. doi:10.1111/j.1523-1755.2005.00296.x

33 Bailey PK, Tomson CRV, Kinra S, *et al.* The effect of rural-to-urban migration on renal function in an Indian population: Cross-sectional data from the Hyderabad arm of the Indian Migration Study. *BMC Nephrol* 2013;**14**. doi:10.1186/1471-2369-14-240

34 Lunyera J, Mohottige D, von Isenburg M, *et al.* CKD of uncertain etiology: A systematic review. *Clin J Am Soc Nephrol* 2016;**11**:379–85. doi:10.2215/CJN.07500715

35 Peel MC, Finlayson BL, McMahon TA. Updated world map of the Koppen-Geiger climate classification. *Hydrol Earth Syst Sci* 2007;**11**:1633–1644.

36 Norwegian Meteorological Institute and the Norwegian Broadcasting Corporation. Yr.

37 Levey AS, Coresh J. Chronic kidney disease. *Lancet* 2012;**379**:165–80. doi:10.1016/S0140-6736(11)60178-5

38 Eastwood JB, Kerry SM, Plange-Rhule J, *et al.* Assessment of GFR by four methods in adults in Ashanti, Ghana: the need for an eGFR equation for lean African populations. *Nephrol Dial Transplant* 2010;**25**:2178–87. doi:10.1093/ndt/gfp765

39 Teo BW, Xu H, Wang D, *et al.* GFR estimating equations in a multiethnic Asian population. *Am J Kidney Dis* 2011;**58**:56–63. doi:10.1053/j.ajkd.2011.02.393

40 Singh AK, Farag YMK, Mittal B V., *et al.* Epidemiology and risk factors of chronic kidney disease in India - Results from the SEEK (Screening and Early Evaluation of Kidney Disease) study. *BMC Nephrol* 2013;**14**:1. doi:10.1186/1471-2369-14-114

41 Barai S, Bandopadhayaya GP, Patel CD, *et al.* Do healthy potential kidney donors in India have an average glomerular filtration rate of 81.4 ml/min? *Nephron - Physiol* 2005;**101**:21–6. doi:10.1159/000086038

42 Srinivas S, Annigeri RA, Mani MK, *et al.* Estimation of glomerular filtration rate in South Asian healthy adult kidney donors. *Nephrology* 2008;**13**:440–6. doi:10.1111/j.1440-1797.2008.00967.x

For peer review only - http://bmjopen.bmj.com/site/about/guidelines.xhtml

43 Caplin B, Jakobsson K, Glaser J, *et al.* International Collaboration for the Epidemiology of eGFR in Low and Middle Income Populations - Rationale and core protocol for the Disadvantaged Populations eGFR Epidemiology Study (DEGREE). *BMC Nephrol* 2017;**18**:1–8. doi:10.1186/s12882-016-0417-1

For peer review only

For peer review only - http://bmjopen.bmj.com/site/about/guidelines.xhtml

477 **FIGURES LEGENDS**

478 **Figure 1** Study areas

479 **Figure 2** Study flowchart

480 **Figure 3** Prevalence ratio of eGFR<60 by age group between rural and urban areas

For peer review only

For peer review only - http://bmjopen.bmj.com/site/about/guidelines.xhtml

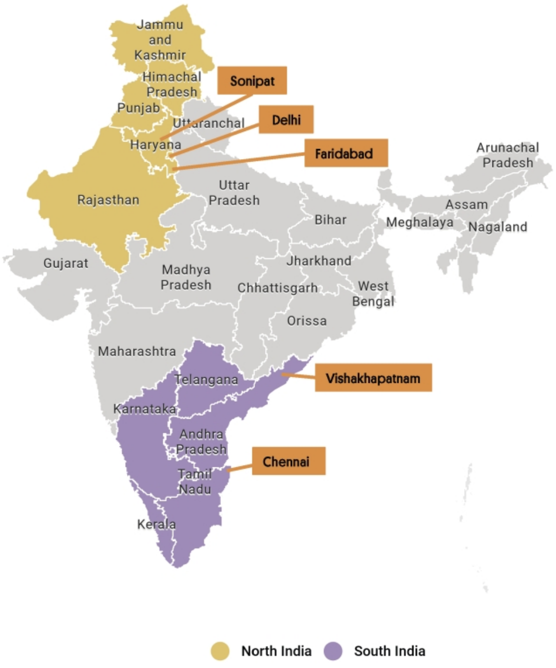
Jammu and Kashmir
Himachal Pradesh
Punjab
Haryana
Uttaranchal
Rajasthan
Uttar Pradesh
Bihar
Meghalaya
Assam
Nagaland
Arunachal Pradesh
Gujarat
Madhya Pradesh
Chhattisgarh
Jharkhand
West Bengal
Orissa
Maharashtra
Telangana
Karnataka
Andhra Pradesh
Tamil Nadu
Kerala
Sonipat
Delhi
Faridabad
Vishakhapatnam
Chennai
North India
South India

Study areas

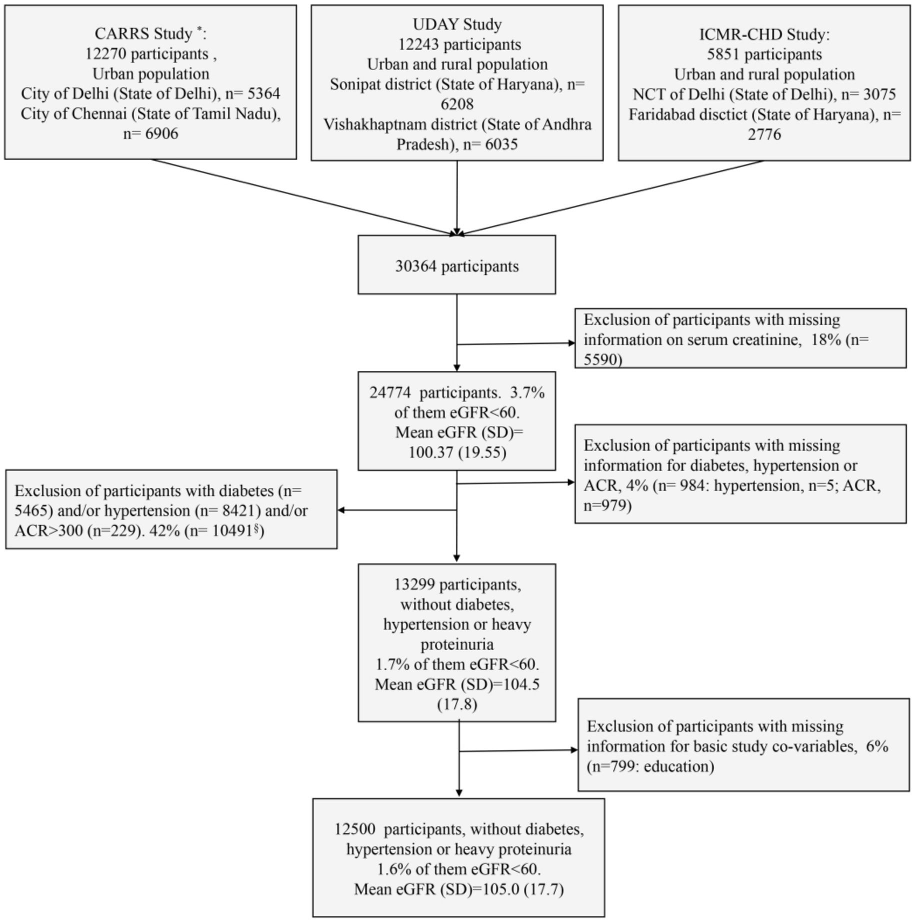

* The original sample size in the CARRS study is 12271, one transgender person was excluded for the current analysis; § 2353 participants with diabetes only; 5185 participants with hypertension only; 35 participants with ACR>30 only; 2724 participants with diabetes, and hypertension; 35 participants with diabetes and ACR>30; 47 participants with hypertension and ACR>30; 112 participants with diabetes, hypertension and ACR>30.

Study flowchart

299x319mm (300 x 300 DPI)

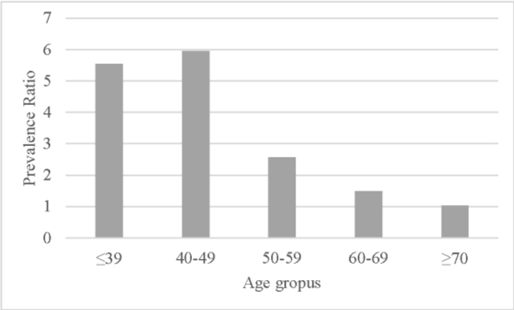

Prevalence ratio of eGFR<60 by age group between rural and urban areas

420x594mm (300 x 300 DPI)

SUPPLEMENTARY MATERIAL

Content

Table S1. Sociodemographic and anthropometric characteristics of overall study participants (prior to exclusion of population with diabetes, hypertension and proteinuria)

Table S2. Associations between sociodemographic and anthropometric characteristics and estimated glomerular filtration rate (eGFR) and eGFR<60 by sex

Table S3. Multiple regression analysis of sociodemographic and anthropometric characteristics associated with eGFR and eGFR<60 including study participants with proteinuria (but without diabetes or hypertension)

Table S4. Multiple regression analysis of sociodemographic and anthropometric characteristics associated with eGFR and eGFR<60 including fasting plasma glucose, HbA1c and systolic blood pressure

For peer review only

Table S1. Sociodemographic and anthropometric characteristics of overall study participants (prior to exclusion of population with diabetes, hypertension and proteinuria)

Variable	n (%) [a] n=24774	eGFR categories, n(%) [b]			
		mean (SD)	≥90	90-60	<60
**Socio-demographic factors**					
Age (years)					
<39	9007 (36)	112.9 (14.9)	8248 (92)	716 (8)	43 (0)
40-49	6924 (28)	101.9 (14.8)	5617 (81)	1215 (18)	92 (1)
50-59	4524 (18)	92.9 (15.2)	2997 (66)	1378 (30)	149 (3)
60-69	3045 (12)	82.8 (17.1)	1410 (46)	1315 (43)	320 (11)
>=70	1274 (5)	72.0 (17.3)	164 (13)	806 (63)	304 (24)
Sex					
Female	13433 (54)	102.6 (19.5)	10404 (77)	2585 (19)	444 (3)
Male	11341 (46)	97.7 (19.3)	8032 (71)	2845 (25)	464 (4)
Education (number of years)					
0	4794 (19)	97.7 (20.2)	3458 (72)	1075 (22)	261 (5)
5	3194 (13)	101.7 (19.4)	2456 (77)	625 (20)	113 (4)
10	8855 (36)	103.2 (18.9)	6995 (79)	1620 (18)	240 (3)
> 10	6358 (26)	100.0 (19.2)	4638 (73)	1538 (24)	182 (3)
Missing data	1573 (6)		889 (57)	572 (36)	112 (7)
Area [d]					
Urban	17732 (72)	102 (19.5)	13577 (77)	3602 (20)	553 (3)
Rural	7042 (28)	96.3 (19.1)	4859 (69)	1828 (26)	355 (5)
Latitude [e]					
North	13570 (55)	98.1 (19.1)	9599 (71)	3439 (25)	532 (4)
South	11204 (45)	103.1 (19.7)	8837 (79)	1991 (18)	376 (3)
**Life-style factors**					
Current smoking					
No	18402 (74)	101.5 (19.6)	13920 (76)	3838 (21)	644 (3)
Yes	6372 (26)	97.1 (19.1)	4516 (71)	1592 (25)	264 (4)
Alcohol consumption ever					
No	19588 (79)	100.9 (19.6)	14671 (75)	4203 (21)	714 (4)
Yes	5186 (21)	98.5 (19.1)	3765 (73)	1227 (24)	194 (4)
Vegetarian					
No	15043 (61)	102.7 (19.7)	11721 (78)	2835 (19)	487 (3)
Yes	9731 (39)	96.8 (18.9)	6715 (69)	2595 (27)	421 (4)
**Biological factors**					
Body mass index (kg/m^2)					
Underweight (≤18.5)	10297 (42)	100.1 (19.6)	7626 (74)	2284 (22)	387 (4)
Normal (>18.5 - ≤25)	2403 (10)	101.58 (20.5)	1838 (76)	471 (20)	94 (4)
Overweight (>25 - ≤30)	7221 (29)	99.9 (18.8)	5309 (74)	1680 (23)	232 (3)
Obese (>30)	3286 (13)	99.3 (19.2)	2392 (73)	766 (23)	128 (4)
Missing data	1567 (6)		1271 (81)	229 (15)	67 (4)
Fat free mass (kg)					
1st tertile (≤37)	7141 (29)	101.9 (20.1)	5481 (77)	1381 (19)	279 (4)
2nd tertile (>37 - <45)	7141 (29)	101.3 (19.1)	5419 (76)	1487 (21)	235 (3)

3rd tertile (≥45)	7141 (29)	98.3 (18.6)	5110 (72)	1797 (25)	234 (3)
Missing data	3351 (14)		2426 (72)	765 (23)	160 (5)

[a] Percentages in columns; [b] percentages in rows; [d] Urban areas include Delhi, Chennai and Sonipat district. Rural areas include Sonipat, Vishakhapatnam and Faridabad districts; [e] North areas include Delhi, Sonipat and Faridabad district. South areas include Chennai and Vishakhapatnam districts.

For peer review only

Table S2. Associations between sociodemographic and anthropometric characteristics and estimated glomerular filtration rate (eGFR) and eGFR<60 by sex

	Men, n=5 434			Women, n=7 066		
**Variable**		**eGFR**	**eGFR<60**		**eGFR**	**eGFR<60**
	**n (%)**	**estimate (95%CI)** [a]	**OR (95% CI)** [a]	**n (%)**	**estimate (95%CI)** [a]	**OR (95% CI)** [a]
Age (years) [b]						
<39	2335 (43)	0.00 (ref)	1.00 (ref)	3786 (54)	0.00 (ref)	1.00 (ref)
40-49	1568 (29)	-9 (-9.97, -8.03)	2.36 (1.2, 4.62)	1908 (27)	-12.52 (-13.29, -11.76)	4.5 (1.95, 10.36)
50-59	843 (16)	-16.84 (-18.03, -15.65)	3.82 (1.91, 7.66)	863 (12)	-21.51 (-22.53, -20.48)	11.78 (5.2, 26.68)
60-69	479 (9)	-25.35 (-26.83, -23.86)	13.07 (6.97, 24.49)	414 (6)	-30.05 (-31.46, -28.64)	32.95 (14.87, 73.02)
>=70	209 (4)	-34.26 (-36.4, -32.12)	31.08 (16.33, 59.17)	95 (1)	-34.78 (-37.6, -31.96)	43.43 (15.93, 118.37)
Education (number of completed years)						
0	823 (15)	0.00 (ref)	1.00 (ref)	1997 (28)	0.00 (ref)	1.00 (ref)
≤5	703 (13)	3.28 (1.82, 4.74)	0.24 (0.13, 0.46)	1006 (14)	0.73 (-0.27, 1.73)	0.81 (0.42, 1.56)
6-≤10	2363 (43)	1.68 (0.51, 2.84)	0.31 (0.20, 0.48)	2454 (35)	0.67 (-0.13, 1.48)	0.43 (0.21, 0.86)
> 10	1545 (28)	-1.35 (-2.6, -0.1)	0.27 (0.15, 0.47)	1609 (23)	-2.39 (-3.27, -1.5)	0.76 (0.40, 1.46)
Area [c]	3583 (66)					
Urban	1851 (34)	0.00 (ref)	1.00 (ref)	4911 (70)	0.00 (ref)	1.00 (ref)
Rural		-4.02 (-4.85, -3.19)	2.72 (1.84, 4.01)	2155 (30)	-3.69 (-4.36, -3.02)	1.99 (1.26, 3.14)
Latitude [d]						
North	2861 (53)	0.00 (ref)	1.00 (ref)	3402 (48)	0.00 (ref)	1.00 (ref)
South	2573 (47)	-1.52 (-2.3, -0.74)	1.76 (1.21, 2.56)	3664 (52)	2.58 (1.96, 3.19)	1.30 (0.83, 2.05)
Current tobacco consumption						
No	2804 (52)	0.00 (ref)	1.00 (ref)	6553 (93)	0.00 (ref)	1.00 (ref)
Yes	2630 (48)	1.15 (0.36, 1.93)	1.32 (0.91, 1.92)	513 (7)	-1.93 (-3.14, -0.73)	1.54 (0.87, 2.73)
Alcohol consumption ever						
No	3035 (56)	0.00 (ref)	1.00 (ref)	7059 (100)	0.00 (ref)	1.00 (ref)

Yes	2399 (44)	-0.71 (-1.49, 0.06)	1.57 (1.08, 2.27)	7 (0)	-9.29 (-18.97, 0.4)	1.00 (1.00, 1.00)
Vegetarian						
No	3576 (66)	0.00 (ref)	1.00 (ref)	4396 (62)	0.00 (ref)	1.00 (ref)
Yes	1858 (34)	0.65 (-0.18, 1.48)	0.61 (0.41, 0.90)	2670 (38)	-2.11 (-2.75, -1.47)	0.70 (0.44, 1.11)
Body mass index (kg/m2)						
Underweight (≤18.5)	2888 (56)	0.00 (ref)	1.00 (ref)	2991 (44)	0.00 (ref)	1.00 (ref)
Normal (>18.5 - ≤25)	812 (16)	4.05 (2.92, 5.18)	0.69 (0.42, 1.14)	764 (11)	1.61 (0.57, 2.65)	1.07 (0.57, 2.03)
Overweight (>25 - ≤30)	1209 (23)	-1.7 (-2.68, -0.73)	0.71 (0.42, 1.21)	2104 (31)	-0.11 (-0.84, 0.62)	0.67 (0.38, 1.20)
Obese (>30)	243 (5)	-0.71 (-2.61, 1.18)	0.36 (0.09, 1.50)	907 (13)	-0.64 (-1.61, 0.33)	0.55 (0.23, 1.31)
Fat free mass (kg)						
1st tertile (≤37)	361 (8)	0.00 (ref)	1.00 (ref)	3833 (58)	0.00 (ref)	1.00 (ref)
2nd tertile (>37 - <45)	1351 (28)	-0.42 (-2.10, 1.25)	0.78 (0.44, 1.38)	2535 (39)	-1.39 (-2.04, -0.74)	0.67 (0.38, 1.17)
3rd tertile (≥45)	3093 (64)	-3.75 (-5.35, -2.16)	0.50 (0.28, 0.90)	208 (3)	-1.36 (-3.17, 0.45)	0.58 (0.08, 4.25)

[a] Adjusted for age; [b] Not adjusted for age; [c] Urban areas include Delhi, Chennai and Sonipat district. Rural areas include Sonipat, Vishakhapatnam and Faridabad districts; [d] North areas include Delhi, Sonipat and Faridabad district. South areas include Chennai and Vishakhapatnam districts.

view only

Table S3. Multiple regression analysis of sociodemographic characteristics associated with eGFR and eGFR<60 including study participants with proteinuria (but without diabetes or hypertension), n=12533

	**eGFR**	**eGFR<60**
**Variable**	**Coefficient (95%CI)** [a]	**OR (95%CI)** [a]
Area [b]		
Urban	0.00 (ref)	1.00 (ref)
Rural	-4.59 (-5.14, -4.03)	1.93 (1.40, 2.66)
Latitude [c]		
North	0.00 (ref)	1.00 (ref)
South	0.29 (-0.21, 0.78)	1.33 (0.98, 1.80)
Education (number of years)		
0	0.00 (ref)	1.00 (ref)
5	0.83 (0, 1.66)	0.55 (0.35, 0.87)
10	0.04 (-0.64, 0.72)	0.51 (0.35, 0.76)
> 10	-3.81 (-4.58, -3.04)	0.66 (0.40, 1.07)
Alcohol consumption ever		
No	0.00 (ref)	1.00 (ref)
Yes	-0.78 (-1.52, -0.05)	1.23 (0.85, 1.79)
Sex		
Female	0.00 (ref)	1.00 (ref)
Male	-2.86 (-3.46, -2.26)	1.38 (0.96, 1.98)
Age (per 10 years)	-9.12 (-9.34, -8.91)	2.23 (2.00, 2.49)

[a] Variables mutually adjusted, [b] Urban areas include Delhi, Chennai and Sonipat district. Rural areas include Sonipat, Vishakhapatnam and Faridabad districts; [c] North areas include Delhi, Sonipat and Faridabad district. South areas include Chennai and Vishakhapatnam districts.

For peer review only

For peer review only - http://bmjopen.bmj.com/site/about/guidelines.xhtml

Table S4. Multiple regression analysis of sociodemographic characteristics associated with eGFR and eGFR<60 including plasma fasting glucose, HbA1c and systolic blood pressure

	eGFR	eGFR<60
**Variable**	**Coefficient (95%CI) [a]**	**OR (95%CI) [a]**
Area [b]		
Urban	0.00 (ref)	1.00 (ref)
Rural	-4.94 (-5.51, -4.38)	2.29 (1.64, 3.20)
Latitude [c]		
North	0.00 (ref)	1.00 (ref)
South	0.23 (-0.26, 0.72)	1.30 (0.95, 1.77)
Education (number of years)		
0	0.00 (ref)	1.00 (ref)
5	1.03 (0.20, 1.86)	0.49 (0.31, 0.79)
10	0.19 (-0.49, 0.87)	0.47 (0.32, 0.71)
> 10	-3.53 (-4.30, -2.76)	0.62 (0.38, 1.02)
Alcohol consumption ever		
No	0.00 (ref)	1.00 (ref)
Yes	-0.72 (-1.46, -0.01)	1.32 (0.90, 1.93)
Sex		
Female	0.00 (ref)	1.00 (ref)
Male	-2.69 (-3.29, -2.09)	1.47 (1.01, 2.12)
Age (per 10 years)	-8.93 (-9.16, -8.70)	2.11 (1.89, 2.38)
Fasting plasma glucose (mg/dl)	-0.06 (-0.08, -0.04)	1.01 (1.00, 1.02)
Hb1Ac (%)	0.03 (-0.56, 0.62)	1.95 (1.34, 2.85)
Systolic blood pressure (mm Hg)	-0.06 (-0.84, -0.04)	1.0 (0.99, 1.02)

[a] Variables mutually adjusted, [b] Urban areas include Delhi, Chennai and Sonipat district. Rural areas include Sonipat, Vishakhapatnam and Faridabad districts; [c] North areas include Delhi, Sonipat and Faridabad district. South areas include Chennai and Vishakhapatnam districts.

For peer review only - http://bmjopen.bmj.com/site/about/guidelines.xhtml

STROBE Statement—Checklist of items that should be included in reports of ***cross-sectional studies***

	Item No	Recommendation	Page/line where the checklist items are located in the paper
**Title and abstract**	1	(*a*) Indicate the study's design with a commonly used term in the title or the abstract	Title and abstract (page 2, lines 29-30)
		(*b*) Provide in the abstract an informative and balanced summary of what was done and what was found	Page 2 , lines 27-45
**Introduction**			
Background/rationale	2	Explain the scientific background and rationale for the investigation being reported	Page 4, lines 68-90
Objectives	3	State specific objectives, including any prespecified hypotheses	Page 4-5, lines 91-95
**Methods**			
Study design	4	Present key elements of study design early in the paper	Page 5, lines 103-111, table 1
Setting	5	Describe the setting, locations, and relevant dates, including periods of recruitment, exposure, follow-up, and data collection	Table 1 and page 6, line 123
Participants	6	(*a*) Give the eligibility criteria, and the sources and methods of selection of participants	Page 5, lines 112-121
Variables	7	Clearly define all outcomes, exposures, predictors, potential confounders, and effect modifiers. Give diagnostic criteria, if applicable	Page 6, lines 149-171
Data sources/ measurement	8*	For each variable of interest, give sources of data and details of methods of assessment (measurement). Describe comparability of assessment methods if there is more than one group	Page 6, lines 123-147
Bias	9	Describe any efforts to address potential sources of bias	Page 6, lines 123-127, 134-138, 145-147
Study size	10	Explain how the study size was arrived at	Figure 1
Quantitative variables	11	Explain how quantitative variables were handled in the analyses. If applicable, describe which groupings were chosen and why	Page 6, lines 123-147
Statistical methods	12	(*a*) Describe all statistical methods, including those used to control for confounding	Page 6, 154-160
		(*b*) Describe any methods used to examine subgroups and interactions	Page 6, line 156; page 6, lines 162-171
		(*c*) Explain how missing data were addressed	Page 6, lines 160-162
		(*d*) If applicable, describe analytical methods taking account of sampling strategy	-
		(*e*) Describe any sensitivity analyses	Page 10, lines 233-235, 139-242
**Results**			
Participants	13*	(a) Report numbers of individuals at each stage of study—eg numbers potentially eligible, examined for	Figure 1

For peer review only

		eligibility, confirmed eligible, included in the study, completing follow-up, and analysed	
		(b) Give reasons for non-participation at each stage	Figure 1
		(c) Consider use of a flow diagram	Figure 1
Descriptive data	14*	(a) Give characteristics of study participants (eg demographic, clinical, social) and information on exposures and potential confounders	Table 2.; page 7-8, lines 177-185
		(b) Indicate number of participants with missing data for each variable of interest	Page 6, lines 160-162
Outcome data	15*	Report numbers of outcome events or summary measures	Page 8, line 187-201
Main results	16	(*a*) Give unadjusted estimates and, if applicable, confounder-adjusted estimates and their precision (eg, 95% confidence interval). Make clear which confounders were adjusted for and why they were included	Page 8, lines 203-220; table 3 and table 4
		(*b*) Report category boundaries when continuous variables were categorized	Table 3 and Table 4
		(*c*) If relevant, consider translating estimates of relative risk into absolute risk for a meaningful time period	-
Other analyses	17	Report other analyses done—eg analyses of subgroups and interactions, and sensitivity analyses	Page 9, lines 221-231; page 10, lines 233-250
**Discussion**			
Key results	18	Summarise key results with reference to study objectives	Page 10, lines 252-263
Limitations	19	Discuss limitations of the study, taking into account sources of potential bias or imprecision. Discuss both direction and magnitude of any potential bias	Pages 12-13, lines 310-322
Interpretation	20	Give a cautious overall interpretation of results considering objectives, limitations, multiplicity of analyses, results from similar studies, and other relevant evidence	Pages 11-12, lines 264-309
Generalisability	21	Discuss the generalisability (external validity) of the study results	Pages 13, lines 323-325
**Other information**			
Funding	22	Give the source of funding and the role of the funders for the present study and, if applicable, for the original study on which the present article is based	Page 3, lines 59-66

*Give information separately for exposed and unexposed groups.

Note: An Explanation and Elaboration article discusses each checklist item and gives methodological background and published examples of transparent reporting. The STROBE checklist is best used in conjunction with this article (freely available on the Web sites of PLoS Medicine at http://www.plosmedicine.org/, Annals of Internal Medicine at http://www.annals.org/, and Epidemiology at http://www.epidem.com/). Information on the STROBE Initiative is available at www.strobe-statement.org.

For peer review only

Prevalence of and risk factors for chronic kidney disease of unknown aetiology in India: secondary data analysis of three population-based cross-sectional studies

Journal:	*BMJ Open*
Manuscript ID	bmjopen-2018-023353.R2
Article Type:	Research
Date Submitted by the Author:	26-Oct-2018
Complete List of Authors:	O'Callaghan Gordo, Cristina; Instituto de Salud Global Barcelona, Campus Mar<br>Shivashankar, Roopa; Public Health Foundation of India,<br>Anand, Shuchi; Stanford Hospital and Clinics,<br>Ghosh, Shreeparna; Public Health Foundation of India,<br>Glaser, Jason; La Isla Foundation; London School of Hygiene and Tropical Medicine, 3Department of Non-communicable Disease Epidemiology<br>Gupta, Ruby; Publichealth Foundation of India<br>Jakobsson , Kristina ; Lunds Universitet Arbets- och miljomedicin<br>Kondal, Dimple; Publichealth Foundation of India<br>Krishnan , Anand ; All India Institute of Medical Sciences Centre for Community Medicine<br>Mohan, Sailesh; Public Health Foundation of India,<br>Mohan, V; Madras Diabetes Research Foundation<br>Nitsch, Dorothea; LSHTM<br>PA , Praveen ; All India Institute of Medical Sciences, Department of Endocrinology and Metabolism<br>Tandon, Nikhil; All India Institute of Medical Sciences,<br>Narayan, K; Emory University School of Public Health, Global Health<br>Pearce, Neil; London School of Hygiene and Tropical Medicine<br>Caplin, Ben; University College London Medical School, Centre for Nephrology,<br>Prabhakaran, Dorairaj; Public Health Foundation of India, Centre for Control of Chronic Conditions and Injuries
**Primary Subject Heading**:	Epidemiology
Secondary Subject Heading:	Global health
Keywords:	EPIDEMIOLOGY, NEPHROLOGY, Chronic renal failure < NEPHROLOGY, PUBLIC HEALTH

TITLE PAGE

Title: Prevalence of and risk factors for chronic kidney disease of unknown aetiology in India: secondary data analysis of three population-based cross-sectional studies

Authors names and affiliations:

Cristina O'Callaghan-Gordo[1,2,3,4*]; Roopa Shivashankar[5, 6*]; Shuchi Anand [7]; Shreeparna Ghosh[5]; Jason Glaser[4, 8]; Ruby Gupta[5]; Kristina Jakobsson[9,10]; Dimple Kondal[5, 6]; Anand Krishnan[11]; Sailesh Mohan[5]; Viswanathan Mohan[12, 13]; , Dorothea Nitsch[14]; Praveen PA[6, 15]; Nikhil Tandon[15]; K M Venkat Narayan[16]; Neil Pearce[4, 17]; Ben Caplin[18**]; Dorairaj Prabhakaran[5, 6**].

[1] ISGlobal, Barcelona, Spain; [2] Universitat Pompeu Fabra (UPF), Barcelona, Spain; [3] CIBER Epidemiología y Salud Pública (CIBERESP), Madrid, Spain; [4] Department of Medical Statistics, London School of Hygiene and Tropical Medicine, London, UK; [5] Public Health Foundation of India (PHFI), New Delhi, India; [6] Centre for Control of Chronic Conditions (4Cs), New Delhi, India; [7] Stanford University School of Medicine; [8] La Isla Network; [9] Occupational and Environmental Medicine, Sahlgrenska Academy, Gothenburg University, Sweden; [10] Occupational and Environmental Medicine, Lund University, Sweden; [11] Centre for Community Medicine, All India Institute of Medical Sciences, New Delhi.; [12] Diabetes Research, Madras Diabetes Research Foundation, Chennai, India; [13] Dr. Mohan's Diabetes Specialities Centre, Chennai, India; [14] Department of Epidemiology and Population Health, London School of Hygiene and Tropical Medicine, , London, UK; [15] Department of Endocrinology and Metabolism, All India Institute of Medical Sciences, New Delhi, India; [16] Emory Global Diabetes Research Center, Rollins School of Public Health, Emory University, Atlanta, GA, USA; [17] Centre for Global NCDs, London School of Hygiene and Tropical Medicine, London, UK; [18] Centre for Nephrology, University College London Medical School, London, UK.

[*] Joint first authors; [**] Joint last authors

Corresponding author's name and email address:

Cristina O'Callaghan-Gordo, **cristina.ocallaghan@isglobal.org**

For peer review only - http://bmjopen.bmj.com/site/about/guidelines.xhtml

Word count: 3919

For peer review only

For peer review only - http://bmjopen.bmj.com/site/about/guidelines.xhtml

ABSTRACT

Objectives: To assess whether chronic kidney disease of unknown aetiology (CKDu) is present in India and to identify risk factors for it using population-based data and standardised methods.

Design: Secondary data analysis of three population-based cross-sectional studies conducted between 2010-2014.

Setting: Urban and rural areas of Northern India (states of Delhi and Haryana) and Southern India (states of Tamil Nadu and Andhra Pradesh)

Participants: 12,500 individuals without diabetes, hypertension or heavy proteinuria

Outcome measures: Mean estimated the glomerular filtration rate (eGFR) and prevalence of eGFR below 60ml/min per 1.73m^2 (eGFR<60) in individuals without diabetes, hypertension or heavy proteinuria (proxy definition of CKDu).

Results: The mean eGFR was 105.0±17.8 ml/min per 1.73m^2. The prevalence of eGFR<60 was 1.6% (95%CI=1.4, 1.7), but this figure varied markedly between areas, being highest in rural areas of Southern Indian [4.8% (3.8, 5.8)]. In Northern India, older age was the only risk factor associated with lower mean eGFR and eGFR<60 [regression coefficient (95%CI)= -0.94 (0.97, 0.91); OR (95%CI)=1.10 (1.08, 1.11)]. In Southern India, risk factors for lower mean eGFR and eGFR<60 respectively were residence in a rural area [-7.78 (-8.69, -6.86); 4.95 (2.61, 9.39)], older age [-0.90 (-0.93, -0.86); 1.06 (1.04, 1.08)] and less education [-0.94 (-1.32, -0.56); 0.67 (0.50, 0.90) for each five years at school].

Conclusions: CKDu is present in India and is not confined to Central America and Sri Lanka. Identified risk factors are consistent with risk factors previously reported for CKDu in Central America and Sri Lanka.

KEYWORDS

Epidemiology; Chronic kidney disease; Chronic kidney disease of unknown aetiology; India; Rural population

ARTICLE SUMMARY

Strengths and limitations of this study

- The use of a random selection of population-based participants allows the estimation of CKDu prevalence in the general population.

For peer review only - http://bmjopen.bmj.com/site/about/guidelines.xhtml

- A large sample size including participants from different areas of India (urban and rural, and Northern and Southern India) increases the representativeness of the results.
- The use of standardized definitions of CKDu facilitates international comparisons of CKDu prevalence and risk factors.
- The prevalence of eGFR<60 observed in this study is likely to be underestimated; however, this is unlikely to have biased the internal comparisons conducted in this study.

FUNDING

This work was supported in part by grant MR/P02386X/1 from the United Kingdom Medical Research Council under the Global Challenges Research Fund. It was also supported by grants from the Colt Foundation and the La Isla Foundation. The CARRS study was funded with federal funds from the National Heart, Lung, and Blood Institute, National Institutes of Health, under Contract No. HHSN2682009900026C. UDAY study was funded by Eli Lilly Foundation. ICMR-CHD study was funded by the Indian Council Medical Research (ICMR). The Centre for Global NCDs is supported by the Wellcome Trust Institutional Strategic Support Fund (097834/Z/11/B). CO-G was supported by a Sara Borrell postdoctoral fellowship awarded from the Carlos III National Institute of Health, Spain (CD13/00072).

For peer review only

For peer review only - http://bmjopen.bmj.com/site/about/guidelines.xhtml

INTRODUCTION

High prevalence of CKDu has mainly been reported in the last decades amongst the working age populations of agricultural communities of tropical/subtropical regions, specifically in Central America and Sri Lanka [1–3]. In Nicaragua and El Salvador, the estimated prevalence of estimated glomerular filtration rate (eGFR; the clinical measure of kidney function) below 60ml/min per 1.73m^2 (eGFR<60), in the absence of diabetes and hypertension, was 10-20% [4–6]. It has been suggested that CKDu may also be highly prevalent in other low and middle income countries (LMICs), including India [7–11]. However, it is not clear in which other regions of the world CKDu occurs, whether the underlying aetiology is the same in different regions and what the risk factors are. Currently, there is no consensus but factors such as heat stress, strenuous work, climatic conditions, agrochemical use, heavy metal exposure and infections have been suggested as risk factors [1,12–15].

Data on CKDu from India are scarce. The recent report of verbal autopsy data from India suggests CKD of all causes is a growing problem. However, it does not provide accurate population-based data on CKDu [16,17]. Existing reports indicate that CKDu may be common but it is difficult to be definite about this because of the absence of population-based studies using standardised and comparable methods. Data from the Indian CKD Registry, a hospital based registry of incident cases of CKD between 2006-2010, found that CKDu was the second commonest form of CKD after diabetic nephropathy [10]. However, this is restricted to referred cases and therefore may not be representative of the general population. There are also sporadic reports of high numbers of CKDu cases among agricultural communities of the South Eastern Indian states of Andhra Pradesh and Odisha (reviewed by Chatterejee [18] and Ganguli [19]). However, population-based data have not been reported for India.

We conducted a secondary analysis of representative sample surveys conducted in India between 2010-2014. Given the absence of a clear case definition for CKDu it is necessary to make a presumptive diagnosis based on measures/estimates of GFR in the absence of known risk factors for kidney disease. The overall aim of the current study was to use a methodology which is comparable to previous studies elsewhere in the world (particularly in Central America) to assess the extent to which reduced kidney function is a problem in India, and which areas and subpopulations are most affected. We therefore: (i) assessed the distribution eGFR and prevalence of eGFR below 60ml/min per 1.73m^2 (eGFR<60) in Indian populations restricted to those without known risk factors for CKD, i.e.

For peer review only - http://bmjopen.bmj.com/site/about/guidelines.xhtml

diabetes, hypertension or heavy proteinuria; ii) compared these outcomes in North and South India and in urban and rural populations; and (iii) identified the risk factors associated with these outcomes.

METHODS

Study population

We used cross-sectional data from three population-based studies conducted in India: the "Centre for Cardiometabolic Risk Reduction in South Asia" cohort study (CARRS study) [20], the "Implementing a Comprehensive Diabetes Prevention and Management Program" study (UDAY study) [21] and the "prevalence of coronary heart disease repeat survey" study funded by the Indian Council of Medical Research (ICMR-CHD study) [22]. Details on study design and selection of participants from the CARRS, UDAY and ICMR-CHD studies have been previously described [20–22] and are summarized in Table 1. Participants from CARRS, UDAY and ICMR-CHD studies provided informed consent prior to participation. The three studies obtained ethical clearance from the corresponding institutions.

For peer review only

For peer review only - http://bmjopen.bmj.com/site/about/guidelines.xhtml

Table 1. Design and methods of the three studies included in the current analysis

	**CARRS**		**UDAY**				**ICMR-CHD**	
**Latitude (North/South)**	North	South	North		South		North	
**Residence (Urban/Rural)**	Urban		Urban	Rural	Urban	Rural	Urban	Rural
**District (and State)**	Delhi (state of Delhi)	Chennai (state of Tamil Nadu)	Sonipat (state of Haryana)		Vishakhapatnam (state of Andhra Pradesh)		National Capital Territory of Delhi (state of Delhi)	Faridabad (state of Haryana)
**Household sampling**	Multistage cluster random (wards - census enumeration blocks - households)		Multistage cluster random (Census Enumeration blocks (urban) or villages (rural) - households)				Multistage cluster random (wards - census enumeration blocks - households)	Simple cluster random (based on Health and Demographic Surveillance System)
**Individual sampling**	1 man and 1 woman from each household (selected by Kish method, [23].) [b]		1 man and 1 woman from each household (selected by Kish method, [23].) [b]				All adults	
**Age groups included**	≥ 20		≥ 30				≥ 30	
**Exclusion criteria**	Pregnant, bedridden and participants who were unable to comprehend the questionnaires due cognitive deficiencies were excluded							
**Study period**	October 2010 - November 2011		July 2014 - December 2014				August 2010 - January 2012	
**Laboratory** [a]	PHFI [c]	MDRF [d]	PHFI [c]				PHFI [c]	

[a] Study laboratories participated in Randox International Quality Assurance Scheme (RIQAS) for clinical chemistry and HbA1c during the entire study periods. [b] In households where only eligible men or only eligible women were present, we selected just one adult. [c] Public Health Foundation of India; [d] Madras Diabetes Research Foundation

For peer review only

For the current analyses, we excluded participants with missing information on serum creatinine, as this variable was necessary to estimate eGFR. As the focus of our study was CKDu, we excluded participants with known risk factors for CKD (i.e. diabetes and hypertension) or evidence of primary glomerular disease (as assessed by heavy proteinuria) or with missing information for these risk factors. We also excluded participants with missing information on basic co-variables (education) for all the analyses conducted. A study flowchart is presented in Figure 1. We classified participants as having: diabetes, if plasma fasting glucose was ≥126 mg/dl, or glycated haemoglobin A1c (HbA1c) was ≥6.5%, or self-reported diabetes; hypertension, if systolic blood pressure was ≥140 mm Hg, or diastolic blood pressure was ≥90 mm Hg, or self-reported hypertension; and heavy proteinuria, if the albumin/creatinine ratio (ACR) in urine was ≥300 mg/g. We used the CKD-EPI equation to estimate GFR (eGFR) [24].

Data collection and laboratory analyses

Data collection was conducted between October 2010 and December 2014. All three studies used a standardized questionnaire to collect data on age, sex, completed years of education (0, ≤5, >5 - ≤ 10, >10), alcohol intake (ever, never) and dietary habits (vegetarian yes, no). Weight, height and body composition were measured using stadiometers (SECA 214 in the three studies) and electronic bioimpedance measuring instruments (Tanita BC 418 in CARRS and ICMR-CHD studies, and Tanita BC 601 in UDAY study). Body mass index (BMI, kg/m^2) was calculated and categorized (≤18.5: underweight; >18.5 - ≤25: normal weight; >25 - ≤30: overweight; >30: obese) and fat free mass was derived from bioelectric impedance analysis (BIA). In CARRS and ICMR-CHD studies, fat free mass (Kg) was directly measured as previously described [25], whereas in UDAY study, fat free mass was estimated from the percentage of total body fat. To estimate total fat free mass from the percentage of body fat, we calculated the amount of total body fat by multiplying the percentage of body fat by the weight of the participant, and from that value we estimated the amount of fat free mass by subtracting the weight of total body fat from the total weight of the participant. Blood pressure was measured using electronic sphygmomanometers (OMRON (HEM-7080) in CARRS and ICMR-CHD studies, and OMRON (HEM 7200) in UDAY study), as previously reported [20,26]. Stadiometers, electronic bioimpedance measuring instruments, and electronic sphygmomanometes were calibrated before each study, and no re-calibration was needed during the duration of different studies. A fasting venous blood sample was used to measure glucose levels, HbA1c and serum creatinine levels and urine sample to measure albuminuria and creatinuria [20]. Glucose levels were measured using hexokinase/kinetic methods, HbA1c using high-performance liquid

For peer review only - http://bmjopen.bmj.com/site/about/guidelines.xhtml

chromatography, serum creatinine using the rate-blanked and compensated kinetic Jaffe method, traceable to isotope dilution mass spectrometry, and albuminuria using immune turbidmetric method [20]. Samples from UDAY, ICMR-CHD, and samples from CARRS from Delhi were analysed at Public Health Foundation of India (PHFI) laboratory and samples from CARRS from Chennai were analysed at Madras Diabetes Research Foundation (MDRF) laboratory. Both PHFI and MDRF laboratories used the same methodologies and protocols to analyse the samples and participated in Randox International Quality Assurance Scheme (RIQAS) for clinical chemistry and HbA1c during the entire study periods. Data from the three studies were homogenized and merged in a single data set.

Statistical analyses

We reported mean eGFR and prevalence of eGFR<60 according to different characteristics of the study populations. UDAY and CARRS studies did not involve fully random population samples (since sampling was based on households, with one participant per household) and the proportions of study participants with particular outcomes (e.g. eGFR<60), will not be exactly the same (but very similar) to what would have been obtained with genuine random population samples; thus in this paper we refer to the prevalence in the study participants, not overall population prevalence estimates. We used linear regression models to estimate the associations between potential risk factors and eGFR and logistic regression models to estimate the associations between potential risk factors and eGFR<60. We also repeated the analyses separately for males and females. Variables associated with eGFR in the basic analyses (adjusted for age and sex) were considered for the multiple regression analysis. In the final multiple regression model, we included all variables that were of a priori interest and/or had shown independent associations with eGFR. We then checked for multicollinearity for each variable in the multiple regression analyses in comparison with the basic analyses [27]. 6% of participants had missing values for basic co-variables (i.e. education) and were excluded from the analysis. 5% and 9% of participants had missing values for BMI and for fat free mass respectively. These participants were included in the main analysis, but we excluded them to compare models non-adjusted and adjusted for these variables. We calculated prevalence ratios of eGFR<60 for rural versus urban areas in different age groups. Urban areas were defined as "all places with a municipality, corporation, cantonment board or notified town area committee, etc., and all other places which satisfied the following criteria: a minimum population of 5,000; at least 75 per cent of the male main working population engaged in non-agricultural pursuits; and a density of population of at least 400 persons per km^2", according to the 2011 Census of India definition [28]. Finally, we estimated potential

For peer review only - http://bmjopen.bmj.com/site/about/guidelines.xhtml

interactions between urban (versus rural) residence and latitude (Northern India (i.e. states of Delhi and Haryana) versus Southern India (states of Tamil Nadu and Andhra Pradesh). Classification of latitude was done in concordance with the classification of major geographical areas on India defined by the Indian Council of Medical Research [29], Figure 1. We conducted all analyses using Stata version 14 (StataCorp, College Station, TX, USA).

Patient and Public Involvement

Patients were not involved in the design of this analysis.

RESULTS

Characteristics of study participants

12,500 people were eligible for the current analyses (Figure 2). Table 2 summarizes the socio-demographic and anthropometric characteristics of the 12,500 study participants included in this analysis (the same information including participants with known risk factors for CKD (n=24,774) in supplementary material Table S1). The mean (standard deviation (±SD)) age of participants was 41.5±11.6 years. 88% (4,805/5,434) of the male population was formally employed; 76% (5,346/7,066) of women worked on house duties (i.e. housewives). The mean BMI was 24±5.0 kg/m^2 and mean fat free mass was 42±15 kg/m^2. The mean fasting plasma glucose was 91.9±12.3 mg/dl and the mean HbA1c was 5.5±0.4 %. The mean systolic and diastolic blood pressures were 114±12 mm Hg and 74±9 mm Hg, respectively. The median (inter quartile range, IQR) albumin/creatinine ratio (ACR) was 2.4 (4.3) mg/g (after exclusion of those with ACR>300mg/g, n=1,208).

Mean eGFR and prevalence of eGFR<60

The mean eGFR was 105.0±17.8 ml/min per 1.73m^2. The mean eGFR was lower at increasing ages, in males, in inhabitants from rural areas and in those from Northern India, in participants with no formal education, and in participants who reported tobacco consumption, alcohol intake and being vegetarian (Table 2). We observed differences in mean eGFR depending on the area, being 104.5±17.6 in urban areas of Northern India, 100.3±16.2 in rural areas of Northern India, 110.9±15.7 in urban areas of Southern India and 97.4±19.8 in the rural area of Southern India.

For peer review only - http://bmjopen.bmj.com/site/about/guidelines.xhtml

For peer review only

184 The prevalence of eGFR<60 among the study population was 1.6% (95% confidence interval (95% CI)=1.4, 1.9).
185 Seventeen per cent (95% CI=16, 17) of study participants had eGFR≥60 - <90 ml/min per $1.73m^2$ and 82% [95%
186 confidence interval (95% CI)=81, 82] had eGFR≥90 ml/min per $1.73m^2$. The prevalences of different categories of
187 eGFR differed by formal education, tobacco consumption, alcohol intake and vegetarianism (Table 2). Also, we
188 observed marked differences in the prevalence of eGFR<60 depending on the area, being 1.4 % (95% CI=1.1, 1.8) in
189 urban areas of Northern India, 1.9 (95% CI=1.4, 2.6) in rural areas of Northern India, 0.43% (95% CI=0.03, 0.07) in
190 urban areas of Southern India and 4.8 % (95% CI=3.9, 5.9) in the rural area of Southern India. The prevalence ratio of
191 eGFR<60 for rural versus urban residence was higher in participants younger than 50 years (prevalence ratio in age
192 group ≤39=5.5, and prevalence ratio in age group 40 - 49=5.8) than in older participants (Figure 3).

For peer review only - http://bmjopen.bmj.com/site/about/guidelines.xhtml

Table 2. Sociodemographic and anthropometric characteristics of study participants (population without diabetes, hypertension or heavy proteinuria)

**Variable**	**n (%) [a] n=12,500**	**eGFR**	**eGFR categories, n(%) [b]**		
		**mean (SD)**	**≥90**	**90 - 60**	**<60**
**Socio-demographic**					
Age (years)					
<39	6121 (49)	113.8 (14.6)	5656 (92)	443 (7)	22 (0)
40 - 49	3476 (28)	102.5 (14.2)	2864 (82)	572 (16)	40 (1)
50 - 59	1706 (14)	93.9 (14.3)	1163 (68)	503 (29)	40 (2)
60 - 69	893 (7)	85.3 (16.2)	463 (52)	368 (41)	62 (7)
≥70	304 (2)	77.5 (15.1)	62 (20)	201 (66)	41 (13)
Sex					
Female	7066 (57)	107.9 (17.1)	6039 (85)	945 (13)	82 (1)
Male	5434 (43)	101.3 (17.9)	4169 (77)	1142 (21)	123 (2)
Education (number completed years)					
0	2820 (23)	100.7 (19.0)	2165 (77)	551 (20)	104 (4)
≤5	1709 (14)	105.9 (17.3)	1412 (83)	273 (16)	24 (1)
6 - ≤10	4817 (39)	107.2 (16.8)	4095 (85)	675 (14)	47 (1)
>10	3154 (25)	105.0 (17.5)	2536 (80)	588 (19)	30 (1)
Area [c]					
Urban	8494 (68)	107.8 (16.1)	7247 (85)	1171 (14)	76 (1)
Rural	4006 (32)	99.0 (18.0)	2961 (74)	916 (23)	129 (3)
Latitude [d]					
North	6263 (50)	103.0 (17.2)	4967 (79)	1197 (19)	99 (2)
South	6237 (50)	107.0 (18.1)	5241 (84)	890 (14)	106 (2)
**Life-style factors**					
Current tobacco consumption					
No	9357 (75)	106.8 (17.3)	7836 (84)	1406 (15)	115 (1)
Yes	3143 (25)	99.8 (18.1)	2372 (75)	681 (22)	90 (3)
Alcohol consumption ever					

For peer review only - http://bmjopen.bmj.com/site/about/guidelines.xhtml

No	10094 (81)	105.9 (17.4)	8362 (83)	1589 (16)	143 (1)	
Yes	2406 (19)	101.1 (18.5)	1846 (77)	498 (21)	62 (3)	[a]
Vegetarian						
No	7972 (64)	107.0 (18.0)	6690 (84)	1154 (14)	128 (2)	
Yes	4528 (36)	101.6 (16.6)	3518 (78)	933 (21)	77 (2)	
**Biological factors**						
Body mass index (kg/m^2)						
Underweight (≤18.5)	5879 (47)	104.2 (17.9)	4734 (81)	1029 (18)	116 (2)	
Normal (>18.5 - ≤25)	1576 (13)	104.7 (19.3)	1283 (81)	257 (16)	36 (2)	
Overweight (>25 - ≤30)	3313 (27)	105.0 (16.9)	2710 (82)	568 (17)	35 (1)	
Obese (>30)	1150 (9)	105.5 (16.4)	948 (82)	194 (17)	8 (1)	
Missing data	582 (5)		533 (92)	39 (7)	10 (2)	
Fat free mass (kg)						
1st tertile (≤37)	3746 (30)	106.6 (18.1)	3146 (84)	532 (14)	68 (2)	
2nd tertile (>37 - <45)	3801 (30)	105.9 (17.2)	3145 (83)	601 (16)	55 (1)	
3rd tertile (≥45)	3834 (31)	102.1 (17.0)	2981 (78)	801 (21)	52 (1)	
Missing data	1119 (9)		936 (84)	153 (14)	30 (3)	

Percentages in columns; [b] percentages in rows; [c] Urban areas include Delhi, Chennai and Sonipat district. Rural areas include Sonipat, Vishakhapatnam and Faridabad districts; [d] North areas include Delhi, Sonipat and Faridabad district. South areas include Chennai and Vishakhapatnam districts.

For peer review only - http://bmjopen.bmj.com/site/about/guidelines.xhtml

199 **Risk factors for lower eGFR and eGFR<60**

200 As expected, age was an important risk factor for reduced eGFR: eGFR was 9.30 ml/min per 1.73 m^2 (95%CI= -9.51,
201 -9.09, model adjusted for sex) lower for each additional 10 years of age. Additionally, being male, living in a rural
202 setting, and consuming alcohol were associated with decreased mean eGFR (Table 3). Similarly, the odds of eGFR<60
203 also increased with age [OR per 10 years, adjusted for sex (95%CI)=2.34 (2.12, 2.59)] and being male, living in a rural
204 setting, living in Southern India and consuming alcohol were also associated with eGFR<60 (Table 3). In general, risk
205 factors for decreased mean eGFR and for eGFR<60 were similar for men and women (supplementary material, Table
206 S2), but few differences were observed. Regarding mean eGFR, living in Southern India was associated with decreased
207 mean eGFR in men and with increased mean eGFR in women; tobacco consumption was associated with increased
208 mean eGFR in men and with decreased mean eGFR in women; vegetarianism was associated with decreased mean
209 eGFR in women but not in men; and being overweight was associated with decreased mean eGFR but in men but not
210 in women. Regarding risk of eGFR<60, living in Southern India was associated with increased risk of eGFR<60 in
211 men but not in women.

For peer review only

For peer review only - http://bmjopen.bmj.com/site/about/guidelines.xhtml

Table 3. Associations between sociodemographic and anthropometric characteristics and eGFR and eGFR<60

	eGFR	eGFR<60
Variable	Coefficient (95 CI) [a]	OR (95 CI) [a]
Age (years) [b]		
<39	0.00 (ref)	1.00 (ref)
40 - 49	-11.08 (-11.68, -10.47)	3.15 (1.87, 5.32)
50 - 59	-19.43 (-20.20, -18.65)	6.41 (3.80, 10.83)
60 - 69	-27.84 (-28.86, -26.82)	19.68 (12.01, 32.26)
≥70	-35.04 (-36.71, -33.37)	39.23 (22.87, 67.23)
Sex [c]		
Female	0.00 (ref)	1.00 (ref)
Male	-3.55 (- 4.05, -3.06)	1.33 (0.99, 1.78)
Education (number of completed years)		
0	0.00 (ref)	1.00 (ref)
≤5	1.92 (1.09, 2.76)	0.41 (0.26, 0.65)
6 - ≤10	1.27 (0.61, 1.93)	0.36 (0.25, 0.53)
> 10	-1.86 (-2.59, -1.14)	0.40 (0.26, 0.62)
Area [d]		
Urban	0.00 (ref)	1.00 (ref)
Rural	-3.84 (-4.37, -3.32)	2.39 (1.78, 3.22)
Latitude [e]		
North	0.00 (ref)	1.00 (ref)
South	0.86 (0.37, 1.35)	1.55 (1.16, 2.07)
Current tobacco consumption		
No	0.00 (ref)	1.00 (ref)
Yes	0.38 (-0.26, 1.02)	1.39 (1.01, 1.91)
Alcohol consumption ever		

For peer review only - http://bmjopen.bmj.com/site/about/guidelines.xhtml

No	0.00 (ref)	1.00 (ref)
Yes	-0.81 (-1.55, -0.08)	1.57 (1.09, 2.27)
Vegetarian		
No	0.00 (ref)	1.00 (ref)
Yes	-0.99 (-1.50, -0.47)	0.65 (0.48, 0.88)
Body mass index (kg/m2)		
Underweight (≤18.5)	2.96 (2.20, 3.73)	0.81 (0.55, 1.20)
Normal (>18.5 - ≤25)	0.00 (ref)	1.00 (ref)
Overweight (>25 - ≤30)	-0.75 (-1.34, -0.16)	0.68 (0.46, 1.01)
Obese (>30)	-0.71 (-1.59, 0.17)	0.47 (0.23, 0.98)
Fat free mass (kg)		
1st tertile (≤37)	0.00 (ref)	1.00 (ref)
2nd tertile (>37 - <45)	-0.91 (-1.54, -0.28)	0.69 (0.47, 1.03)
3rd tertile (≥45)	-3.90 (-4.77, -3.04)	0.49 (0.31, 0.80)

[a] Adjusted for age and sex; [b] Adjusted just for sex; [c] Adjusted just for age; [d] Urban areas include Delhi, Chennai and Sonipat district. Rural areas include Sonipat, Vishakhapatnam and Faridabad districts; [e] North areas include Delhi, Sonipat and Faridabad district. South areas include Chennai and Vishakhapatnam districts.

For peer review only

For peer review only - http://bmjopen.bmj.com/site/about/guidelines.xhtml

216 In the multiple regression analyses, decreased mean eGFR remained associated with older age, being male, living in a rural setting, and alcohol consumption (Table 4). Risk of eGFR<60 remained associated with older age, being male and living in a rural setting, and having no formal education (Table 4). We adjusted all the multiple regression models for fat free mass and vegetarianism to assess the possibility that differences observed between urban and rural participants were due to differences in diet and/or body composition. These adjustments had little effect on the results (Table 4).

For peer review only

For peer review only - http://bmjopen.bmj.com/site/about/guidelines.xhtml

Table 4. Multiple regression analyses of sociodemographic characteristics associated with eGFR and eGFR<6).

	eGFR Coefficient (95% CI)			eGFR<60 OR (95% CI)		
Variable	Model 1[a]	Model 2[b]	Model 3[c]	Model 1[a]	Model 2[b]	Model 3[c]
Area [d]						
Urban	0.00 (ref)	0.00 (ref)	0.00 (ref)	1.00 (ref)	1.00 (ref)	1.00 (ref)
Rural	-4.57 (-5.13, -4.02)	-3.94 (-4.53, -3.36)	-4.10 (-4.70, -3.51)	1.99 (1.43, 2.76)	1.61 (1.12, 2.30)	1.65 (1.14, 2.37)
Latitude [e]						
North	0.00 (ref)	0.00 (ref)	0.00 (ref)	1.00 (ref)	1.00 (ref)	1.00 (ref)
South	0.31 (-0.18, 0.80)	-0.10 (-0.61, 0.41)	0.26 (-0.37, 0.89)	1.33 (0.98, 1.81)	1.60 (1.14, 2.32)	1.33 (0.86, 2.04)
Education (number of completed years)						
0	0.00 (ref)	0.00 (ref)	0.00 (ref)	1.00 (ref)	1.00 (ref)	1.00 (ref)
≤5	0.94 (0.01, 1.77)	1.16 (0.30, 2.02)	1.18 (0.32, 2.04)	0.50 (0.31, 0.80)	0.44 (0.26, 0.74)	0.45 (0.26, 0.75)
6 - ≤10	0.04 (-0.64, 0.72)	0.21 (-0.49, 0.91)	0.21 (-0.50, 0.92)	0.50 (0.34, 0.75)	0.38 (0.24, 0.60)	0.39 (0.25, 0.62)
>10	-3.81 (-4.6, -3.0)	-3.81 (-4.60, -3.02)	-3.78 (-4.59, -2.97)	0.68 (0.42, 1.11)	0.61 (0.36, 1.03)	0.65 (0.38, 1.11)
Alcohol consumption ever						
No	0.00 (ref)	0.00 (ref)	0.00 (ref)	1.00 (ref)	1.00 (ref)	1.00 (ref)
Yes	-0.85 (-1.58, -0.12)	-0.69 (-1.47, 0.08)	-0.63 (-1.41, 0.15)	1.28 (0.88, 1.87)	1.18 (0.78, 1.79)	1.15 (0.76, 1.74)

For peer review only - http://bmjopen.bmj.com/site/about/guidelines.xhtml

Sex						
Female	0.00 (ref)	0.00 (ref)	0.00 (ref)	1.00 (ref)	1.00 (ref)	1.00 (ref)
Male	-2.85 (-3.44, -2.25)	-3.00 (-3.62, -2.38)	-2.52 (-3.18, -1.86)	1.39 (0.96, 2.01)	1.49 (1.00, 2.21)	1.50 (0.97, 2.31)
Age (per 10 years)	-9.10 (-9.32, -8.88)	-9.09 (-9.32, -8.86)	-9.15 (-9.38, -8.91)	2.21 (1.98, 2.47)	2.25 (2.00, 2.55)	2.27 (2.00, 2.57)
Fat free mass (kg)			-0.04 (-0.06, -0.02)			1.0 (0.98, 1.02)
Vegetarian						
No			0.00 (ref)			1.00 (ref)
Yes			0.66 (-0.03, 1.35)			0.74 (0.47, 1.18)

[a] Model 1 included the following variables: area, latitude, education, alcohol consumption, sex and age; n=12,500**;** [b] Model 2 included the same variables than model 1. Participants with missing information on fat free mass were excluded from the analysis, n=11,381; [c] Model 3 included the same variables than model 1 plus fat free mass and vegetarianism, n=11,381. [d] Urban areas include Delhi, Chennai and Sonipat district. Rural areas include Sonipat, Vishakhapatnam and Faridabad districts; [e] North areas include Delhi, Sonipat and Faridabad district. South areas include Chennai and Vishakhapatnam districts.

For peer review only - http://bmjopen.bmj.com/site/about/guidelines.xhtml

We observed an interaction between the effects of latitude (North/South) and urban/rural residence in association with reduced eGFR (p-value for interaction<0.001). The mean eGFR was lower in rural settings in both Northern and Southern India (controlling for age, sex, education and alcohol intake). However, this decrease was much more marked in Southern India. In Northern India, rural residence, formal education (and duration) and age were the only other risk factor associated with reduced eGFR. In Southern India, being male was also a risk factor for reduced eGFR, whereas formal education was only a risk factor for reduced eGFR among those with more than 10 years of schooling (Table 5). We also observed an interaction between the effects of latitude (North/South) and urban/rural residence in association with eGFR<60 (p-value likelihood-ratio test for interaction<0.001). In Northern India, eGFR<60 was not associated with urban/rural residence, and older age was the only factor associated with eGFR<60. In Southern India, rural residence was the strongest risk factor for eGFR<60 but older age and lower years of formal education also increased the risk of eGFR<60 (Table 5).

For peer review only

For peer review only - http://bmjopen.bmj.com/site/about/guidelines.xhtml

Table 5. Multivariate analysis of sociodemographic characteristics associated with eGFR and with eGFR<60 according to latitude [a]

	eGFR (n=12,500)		eGFR<60(n=12,500)	
	North (n=6263) [a]	South (n=6237) [b]	North (n=6263) [a]	South (n=6237) [b]
Variables	Coefficient (95% CI)	Coefficient (95% CI)	OR (95% CI)	OR (95% CI)
Area [c]				
Urban	0.00 (ref)	0.00 (ref)	1.00 (ref)	1.00 (ref)
Rural	-1.42 (-2.15, -0.70)	-7.90 (-8.81, -7.00)	0.88 (0.57, 1.37)	4.68 (2.50, 8.77)
Education (number of completed years)				
0	0.00 (ref)	0.00 (ref)	1.00 (ref)	1.00 (ref) **
≤5	-1.32 (-2.58, -0.05)	1.05 (-0.06, 2.16)	1.16 (0.57, 2.35)	0.40 (0.20, 0.80)
6-≤10	-3.50 (-4.48, -2.52)	0.28 (-0.74, 1.30)	1.34 (0.74, 2.41)	0.35 (0.16, 0.74)
> 10	-6.93 (-7.97, -5.89)	-2.85 (-4.03, -1.67)	1.34 (0.69, 2.58)	0.61 (0.24, 1.57)
Alcohol consumption ever				
No	0.00 (ref)	0.00 (ref)	1.00 (ref)	1.00 (ref)
Yes	-0.54 (-1.55, 0.47)	-0.06 (-1.11, 0.99)	1.09 (0.62, 1.92)	1.36 (0.74, 2.17)
Sex				
Female	0.00 (ref)	0.00 (ref)	1.00 (ref)	1.00 (ref)
Male	-0.17 (-0.96, 0.63)	-5.40 (-6.29, -4.51)	0.97 (0.59, 1.59)	1.58 (0.91, 2.75)
Age (per 10 years)	-9.26 (-9.55, -8.97)	-8.96 (-9.28, -8.64)	2.51 (2.15, 2.93)	2.10 (1.77, 2.50)

** Likelihood ratio test for linear trend <0.05, OR (95% CI)=0.68 (0.51, 0.91). [a] North areas include Delhi, Sonipat and Faridabad district. [b] South areas include Chennai and Vishakhapatnam districts. [c] Urban areas include Delhi, Chennai and Sonipat district. Rural areas include Sonipat, Vishakhapatnam and Faridabad districts

For peer review only - http://bmjopen.bmj.com/site/about/guidelines.xhtml

Sensitivity analyses

We performed a sensitivity analysis including those with ACR>300 (but without hypertension or diabetes, n=33) as we were concerned that those with CKDu might develop proteinuria at more advanced CKD stages. However, this did not alter the mean eGFR (mean eGFR among the overall study population=105.0±17.8, mean eGFR in this sensitivity analysis=105.0±17.8), nor the estimated prevalence of eGFR<60 (prevalence among the overall study population=1.6%; prevalence in this sensitivity analysis =1.7%). The findings on risk factors were also similar to the findings from the primary analyses (supplementary material, Table S3).

Given concerns about potentially different thresholds to define diabetes and high blood pressure in different ethnic groups [30,31], we performed a further sensitivity analysis including fasting plasma glucose, HbA1c and systolic blood pressure in the multivariate model (even though there is evidence for both causation and reverse causation between these factors and CKD [32]). Systolic blood pressure and fasting plasma glucose were associated with reduced eGFR in this non diabetic population, but inclusion of these variables did not alter the coefficients for the associations with other risk factors observed in the primary analysis (supplementary material, Table S4). HbA1c was associated with eGFR<60 in this non diabetic population but inclusion of this variable did not alter the OR for other risk factors observed in the primary analysis (supplementary material, Table S4). Therefore, although the relationship between sub-clinical diabetes and impaired kidney function requires further prospective investigation, there is no evidence that the excess risk of low eGFR (i.e. lower mean eGFR and higher prevalence of eGFR<60) in rural Southern India is associated with either impaired fasting glucose or higher blood pressure.

DISCUSSION

We report the distribution of eGFR in people without diabetes, hypertension or heavy proteinuria and estimate the prevalence of CKDu in our study population, including participants from urban and rural settings. This is the first population-based evidence, using standardised methods, which indicates that CKDu is present in India and is not confined to Central America and Sri Lanka. We found that the rural population from Southern India (Vishakhapatnam district) had the highest risk of decreased eGFR (lower mean eGFR and higher prevalence of eGFR<60). Risk factors of decreased eGFR were different between Southern and Northern India. In Southern

For peer review only - http://bmjopen.bmj.com/site/about/guidelines.xhtml

India, rural residence, older age and being male were risk factors for both lower mean eGFR and eGFR<60; education was associated with decreased risk for eGFR<60 but not with lower mean eGFR. In Northern India, older age was the only risk factor for both lower mean eGFR and eGFR<60; rural residence and years of formal education were associated with lower mean eGFR but not with eGFR<60. In summary, in Southern India, older age, being male and rural residence were the main risk factors for decreased eGFR, whereas in Norther India older age was the main risk factors for decreased eGFR.

As in Central America, the risk of low eGFR was higher in rural settings than in urban settings. This is in concordance with a previous study from Hyderabad (India), that has provided evidence of a higher risk of low eGFR in a rural population compared to urban-migrant and to urban population [33], and with various studies from other LMICs that have provided evidence of clusters of CKDu among the rural population [2,3]. Exposure to some of the suggested potential risk factors for CKDu such as agricultural work and agrochemical exposure, amongst others [34], may be greater in rural settings. Such exposures may also differ between Southern and Northern India, and potentially explain the differences observed between these areas. The associations between urban/rural residence and lower mean eGFR was much more marked in Southern India than in Northern India, and the associations between urban/rural residence and eGFR<60 was only observed in Southern India. The higher prevalence ratio (for eGFR<60) in the working age population compared to older age groups is consistent with the hypothesis that deceased in eGFR could be potentially explained by occupational exposures. The suggestive sex differences may also support this hypothesis. However, we did not have detailed data on occupation that allowed us to explore these associations in greater detail.

The higher risk of low eGFR in Southern India (Chennai and Vishakhapatnam districts) observed in our study is consistent with the clusters of CKDu cases previously reported in the Southern Indian states of Andhra Pradesh and Odisha [11,18,19]. Visakhapatnam district (state of Andhra Pradesh) and Chennai district (state of Tamil Nadu) have a similar climate than these areas where CKDu clusters have previously reported [35]. In these districts, mean temperatures range from 18 ºC to 37 ºC and rainfall occurs mainly between June and December [36]. On the other hand, sites from Norther India included in the study (Delhi (state of Delhi), Sonipat and Faridabad (Haryana state)), have a different climate. In these districts mean temperature ranges

For peer review only - http://bmjopen.bmj.com/site/about/guidelines.xhtml

297 from 8 ºC to 39 ºC and precipitation occurs mainly between July and August [36]. A previous study conducted
298 in Costa Rica found a spatial correlation between rates of CKD mortality and temperature and rainfall [13].

299 About 5% of the rural population of Vishakhapatnam (Andra Pradesh, Southern India) without diabetes,
300 hypertension or proteinuria had eGFR<60. This figure is almost as high as the prevalence observed in the USA
301 (i.e. 6.7%) including people with diabetes, hypertension or proteinuria [37]. Moreover, the estimates of GFR in
302 our study are likely to be underestimated. The CKD-EPI equation has been standardised for the white and Afro-
303 American population [24], but its validity for other ethnic groups has been questioned [38,39]. Previous studies
304 using CKD-EPI equation to estimate GFR in Indian populations reported mean eGFR values similar to the mean
305 eGFR reported in our study (i.e. 104.9 ± 25.52 ml/min/1.73 m^2) [40]. However, two studies conducted among
306 healthy kidney donors in India (population similar to those included in this analysis) have reported mean
307 (measured) GFR between 81.4 and 95.5 ml/min per 1.73 m^2 [41,42], suggesting that the CKD-EPI equation
308 substantially overestimates eGFR in the Indian population. Therefore, the prevalence of eGFR<60 observed in
309 this study is likely to be substantially underestimated (although this is unlikely to have biased the internal
310 comparisons, e.g. between urban and rural settings). The use of a conservative definition of the population
311 susceptible to CKDu, may have also underestimated the prevalence of eGFR<60 in our study, as the population
312 with diabetes, hypertension or glomerular disease may also have reduced eGFR due to other ('unknown')
313 causes. To estimate the actual prevalence of reduced eGFR, future studies should include validated methods to
314 estimate GFR in the Indian population. We were concerned that the validity of CKD-EPI among the Indian
315 population may be also compromised by differences in muscular mass and meat consumption between
316 population groups within India. We adjusted the analyses for fat free mass and vegetarianism, but this did not
317 alter the results, suggesting no confounding effect by these variables.

318 Our study has at least three potential limitations. First, we only had one measure of eGFR, and therefore we
319 could not differentiate acute kidney injury (AKI) from CKD. This is a common limitation in epidemiological
320 studies, as it is challenging to obtain more than one measure of eGFR at least 3 months apart in large population-
321 based investigations. Therefore, we may have misclassified some cases of AKI as reduced eGFR, and therefore
322 overestimate the prevalence of this condition. Nevertheless, there is no a priori reason to think that potential
323 misclassification was different according to the evaluated risks factors. Second, the three population-based

For peer review only - http://bmjopen.bmj.com/site/about/guidelines.xhtml

studies included in this analysis used different sampling strategies. CARRS and UDAY studies included only one man and one woman from all the eligible participants of selected households, whereas ICMR-CHD included all eligible adults from each selected household. This could have slightly biased our results (including our prevalence estimates) if risk factors potentially associated with CKDu were different between households inhabited only by a man and a women or by extended families. Third, information on other potential risk factors for CKDu, such as infections by leptospora or hantavirus infection, or use of nonsteroidal anti-inflammatory drugs (NSAIDs) was not available.

The main strengths of the study are the use of a random selection of population-based participants and a large sample size including participants from different areas of India (urban and rural, and Northern and Southern India). Moreover, we used the definitions proposed in DRGREE study [43], that aims to allow international comparisons of CKDu prevalence and help in the description of risk factors and in identifying the causes and mechanisms leading to CKDu.

In conclusion, our findings indicate that reduced eGFR, consistent with the definition of CKDu, is common in rural settings of Southern India (Vishakhapatnam district). This results support the hypothesis that the epidemic of CKDu, initially described in agricultural communities of Central America and Sri Lanka, may be common in other rural communities of tropical/subtropical countries. This has important implications for global health, since it indicates that CKDu may have a substantial public health burden globally that has been previously unrecognised. Population-based studies in other tropical/subtropical countries are required to assess the global patterns of burden of disease from CKDu [43].

AUTHOR CONTRIBUTIONS AND ACKNOWLEDGEMENTS

CO-G, BC, NP and DP designed the work; RS, SA, SG, RG, AK, SM, VM, PPA, NT, and KMN collected the data; CO-G and DK conducted the analysis of the data; CO-G, RS, SA, JG, KJ, DN, SM, KMN, NP, BC, and DP interpreted the data of the work. CO-G, RS, BC, and NP drafted the manuscript; RS, SA, SG, JG, RG, KJ, DK, AK, SM, VM, DN, PPA, NT, KMN, and DP revised the manuscript for important intellectual content, provided comments and suggested revisions. All authors approved the final version for publication.

For peer review only

349 We thank Manolis Kogevinas for his comments on the advanced version of the manuscript and Marta Solano 350 for preparing Figure 1.

351 **CONFLICTS OF INTERESTS**

352 The authors declare that they have no competing interests

353 **DATA SHARING STATEMENT**

354 The datasets used and/or analysed during the current study are available from Public Health Foundation of India 355 (PHFI) on reasonable request. Interested investigators should contact PHFI. Computing code can be obtained 356 from the corresponding author.

For peer review only

For peer review only - http://bmjopen.bmj.com/site/about/guidelines.xhtml

REFERENCES

1 Wesseling C, Crowe J, Hogstedt C, *et al.* Mesoamerican Nephropathy: Report from the First International Research Workshop on MeN. Heredia, Costa Rica: 2013.

2 Correa-Rotter R, Wesseling C, Johnson RJ. CKD of unknown origin in Central America: the case for a Mesoamerican nephropathy. *Am J Kidney Dis* 2014;**63**:506–20. doi:10.1053/j.ajkd.2013.10.062

3 Jayatilake N, Mendis S, Maheepala P, *et al.* Chronic kidney disease of uncertain aetiology: Prevalence and causative factors in a developing country. *BMC Nephrol* 2013;**14**:1. doi:10.1186/1471-2369-14-180

4 Lebov JF, Valladares E, Pena R, *et al.* A population-based study of prevalence and risk factors of chronic kidney disease in Leon, Nicaragua. *Can J kidney Heal Dis* 2015;**2**:6. doi:10.1186/s40697-015-0041-1

5 Peraza S, Wesseling C, Aragon A, *et al.* Decreased kidney function among agricultural workers in El Salvador. *Am J Kidney Dis* 2012;**59**:531–40. doi:10.1053/j.ajkd.2011.11.039

6 Torres C, Aragón A, González M, *et al.* Decreased kidney function of unknown cause in Nicaragua: a community-based survey. *Am J Kidney Dis* 2010;**55**:485–96. doi:10.1053/j.ajkd.2009.12.012

7 Seck SM, Doupa D, Gueye L, *et al.* Prevalence of chronic kidney disease and associated factors in senegalese populations: a community-based study in saint-louis. *Nephrourol Mon* 2014;**6**:e19085. doi:10.5812/numonthly.19085

8 Barsoum RS. Burden of chronic kidney disease: North Africa. *Kidney Int Suppl* 2013;**3**:164–6. doi:10.1038/kisup.2013.5

9 El Minshawy O, Ghabrah T, El Bassuoni E. End-stage renal disease in Tabuk Area, Saudi Arabia: an epidemiological study. *Saudi J Kidney Dis Transpl* 2014;**25**:192–5.

10 Rajapurkar MM, John GT, Kirpalani AL, *et al.* What do we know about chronic kidney disease in India: First report of the Indian CKD registry. *BMC Nephrol* 2012;**13**. doi:10.1186/1471-2369-13-10

11 Reddy D V., Gunasekar A. Chronic kidney disease in two coastal districts of Andhra Pradesh, India: Role of drinking water. *Environ Geochem Health* 2013;**35**:439–54. doi:10.1007/s10653-012-9506-7

12 Jayasumana C, Paranagama P, Agampodi S, *et al.* Drinking well water and occupational exposure to Herbicides is associated with chronic kidney disease, in Padavi-Sripura, Sri Lanka -No section-. *Environ Heal A Glob Access Sci Source* 2015;**14**. doi:10.1186/1476-069X-14-6

13 Wesseling C, Van Wendel De Joode B, Crowe J, *et al.* Mesoamerican nephropathy: Geographical distribution and time trends of chronic kidney disease mortality between 1970 and 2012 in Costa Rica. *Occup Environ Med* 2015;**72**:714–21. doi:10.1136/oemed-2014-102799

14 Garcia-Garcia G, Jha V, World Kidney Day Steering Committee. Environmental and occupational factors in CKD. *Occup Environ Med* 2015;**72**:238. doi:10.1136/oemed-2015-102859

15 Robey RB. Cyclical dehydration-induced renal injury and Mesoamerican nephropathy: as sweet by any other name? *Kidney Int* 2014;**86**:226–9. doi:10.1038/ki.2014.47

16 Jha V, Modi G. Uncovering the rising kidney failure deaths in India. *Lancet Glob Heal* 2017;**5**:e14–5. doi:10.1016/S2214-109X(16)30299-6

17 Dare AJ, Fu SH, Patra J, *et al.* Renal failure deaths and their risk factors in India 2001–13: nationally representative estimates from the Million Death Study. *Lancet Glob Heal* 2017;**5**:e89–95. doi:10.1016/S2214-109X(16)30308-4

18 Chatterjee R. Occupational Hazard. *Science (80-)* 1026;**352**:24–7.

19 Ganguli A. Uddanam Nephropathy/Regional Nephropathy in India: Preliminary Findings and a Plea for Further Research. *Am J Kidney Dis* 2016;**68**:344–8. doi:10.1053/j.ajkd.2016.04.012

20 Nair M, Ali MK, Ajay VS, *et al.* CARRS Surveillance study: Design and methods to assess burdens from multiple perspectives. *BMC Public Health* 2012;**12**:1. doi:10.1186/1471-2458-12-701

For peer review only - http://bmjopen.bmj.com/site/about/guidelines.xhtml

21 Mohan S, Jarhyan P, Ghosh S, *et al.* UDAY: Protocol of a Comprehensive Diabetes and Hypertension Prevention and Management Program in India. *BMJ open* 2018;**8**:e015919. doi:e015919. doi: 10.1136/bmjopen-2017-015919

22 Prabhakaran D, Roy A, Praveen PA, *et al.* 20-Year Trend of Cardiovascular Disease Risk Factors. *Glob Heart* Published Online First: 2017. doi:10.1016/j.gheart.2016.11.004

23 World Health Organization. STEPS Manual. 2015.

24 Levey AS, Stevens LA, Schmid CH, *et al.* A new equation to estimate glomerular filtration rate. *Ann Intern Med* 2009;**150**:604–12.

25 Patel SA, Deepa M, Shivashankar R, *et al.* Comparison of multiple obesity indices for cardiovascular disease risk classification in South Asian adults: The CARRS Study. *PLoS One* 2017;**12**:e0174251.

26 Anand S, Shivashankar R, Ali MK, *et al.* Prevalence of chronic kidney disease in two major Indian cities and projections for associated cardiovascular disease. *Kidney Int* 2015;**88**:178–85. doi:10.1038/ki.2015.58

27 Greenland S, Daniel R, Pearce N, *et al.* Outcome modelling strategies in epidemiology: traditional methods and basic alternatives. *Int J Epidemiol* 2016;:1–11. doi:10.1093/ije/dyw040

28 Census of India. 2011.http://censusindia.gov.in/ (accessed 1 Aug 2018).

29 Longvah T, Ananthan R, Bhaskarachary K, *et al.* Indian Food Composition tables. Hyderabad: 2017.

30 Herman WH. Do race and ethnicity impact hemoglobin A1c independent of glycemia? *J Diabetes Sci Technol* 2009;**3**:656–60. doi:10.1177/193229680900300406

31 Modesti PA, Reboldi G, Cappuccio FP, *et al.* Panethnic Differences in Blood Pressure in Europe: A Systematic Review and Meta-Analysis. *PLoS One* 2016;**11**:e0147601. doi:10.1371/journal.pone.0147601

32 Verhave JC, Hillege HL, Burgerhof JGM, *et al.* The association between atherosclerotic risk factors and renal function in the general population. *Kidney Int* 2005;**67**:1967–73. doi:10.1111/j.1523-1755.2005.00296.x

33 Bailey PK, Tomson CRV, Kinra S, *et al.* The effect of rural-to-urban migration on renal function in an Indian population: Cross-sectional data from the Hyderabad arm of the Indian Migration Study. *BMC Nephrol* 2013;**14**. doi:10.1186/1471-2369-14-240

34 Lunyera J, Mohottige D, von Isenburg M, *et al.* CKD of uncertain etiology: A systematic review. *Clin J Am Soc Nephrol* 2016;**11**:379–85. doi:10.2215/CJN.07500715

35 Peel MC, Finlayson BL, McMahon TA. Updated world map of the Koppen-Geiger climate classification. *Hydrol Earth Syst Sci* 2007;**11**:1633–1644.

36 Norwegian Meteorological Institute and the Norwegian Broadcasting Corporation. Yr.

37 Levey AS, Coresh J. Chronic kidney disease. *Lancet* 2012;**379**:165–80. doi:10.1016/S0140-6736(11)60178-5

38 Eastwood JB, Kerry SM, Plange-Rhule J, *et al.* Assessment of GFR by four methods in adults in Ashanti, Ghana: the need for an eGFR equation for lean African populations. *Nephrol Dial Transplant* 2010;**25**:2178–87. doi:10.1093/ndt/gfp765

39 Teo BW, Xu H, Wang D, *et al.* GFR estimating equations in a multiethnic Asian population. *Am J Kidney Dis* 2011;**58**:56–63. doi:10.1053/j.ajkd.2011.02.393

40 Singh AK, Farag YMK, Mittal B V., *et al.* Epidemiology and risk factors of chronic kidney disease in India - Results from the SEEK (Screening and Early Evaluation of Kidney Disease) study. *BMC Nephrol* 2013;**14**:1. doi:10.1186/1471-2369-14-114

41 Barai S, Bandopadhayaya GP, Patel CD, *et al.* Do healthy potential kidney donors in India have an average glomerular filtration rate of 81.4 ml/min? *Nephron - Physiol* 2005;**101**:21–6. doi:10.1159/000086038

42 Srinivas S, Annigeri RA, Mani MK, *et al.* Estimation of glomerular filtration rate in South Asian

For peer review only

For peer review only - http://bmjopen.bmj.com/site/about/guidelines.xhtml

healthy adult kidney donors. *Nephrology* 2008;**13**:440–6. doi:10.1111/j.1440-1797.2008.00967.x

43 Caplin B, Jakobsson K, Glaser J, *et al.* International Collaboration for the Epidemiology of eGFR in Low and Middle Income Populations - Rationale and core protocol for the Disadvantaged Populations eGFR Epidemiology Study (DEGREE). *BMC Nephrol* 2017;**18**:1–8. doi:10.1186/s12882-016-0417-1

For peer review only

For peer review only - http://bmjopen.bmj.com/site/about/guidelines.xhtml

454 **FIGURES LEGENDS**

455 **Figure 1** Study areas

456 **Figure 2** Study flowchart

457 **Figure 3** Prevalence ratio of eGFR<60 for rural versus urban residence in different age groups

For peer review only

For peer review only - http://bmjopen.bmj.com/site/about/guidelines.xhtml

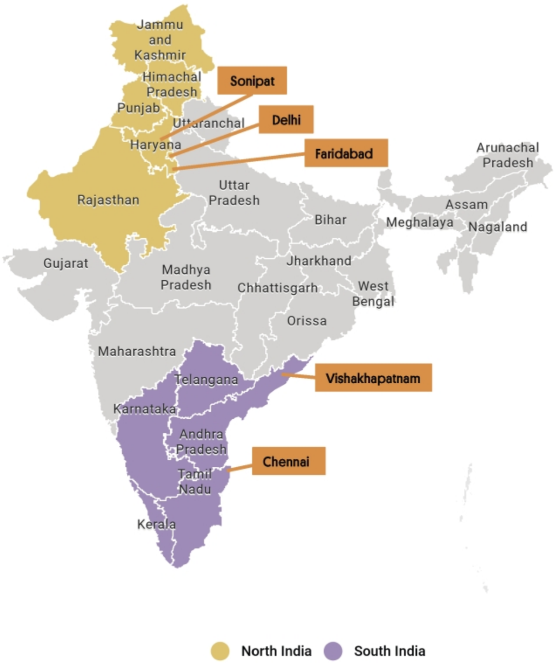
Jammu and Kashmir
Himachal Pradesh
Punjab
Uttaranchal
Haryana
Rajasthan
Uttar Pradesh
Bihar
Arunachal Pradesh
Assam
Meghalaya
Nagaland
Gujarat
Madhya Pradesh
Jharkhand
Chhattisgarh
West Bengal
Orissa
Maharashtra
Telangana
Karnataka
Andhra Pradesh
Tamil Nadu
Kerala
Sonipat
Delhi
Faridabad
Vishakhapatnam
Chennai
North India
South India

Study areas

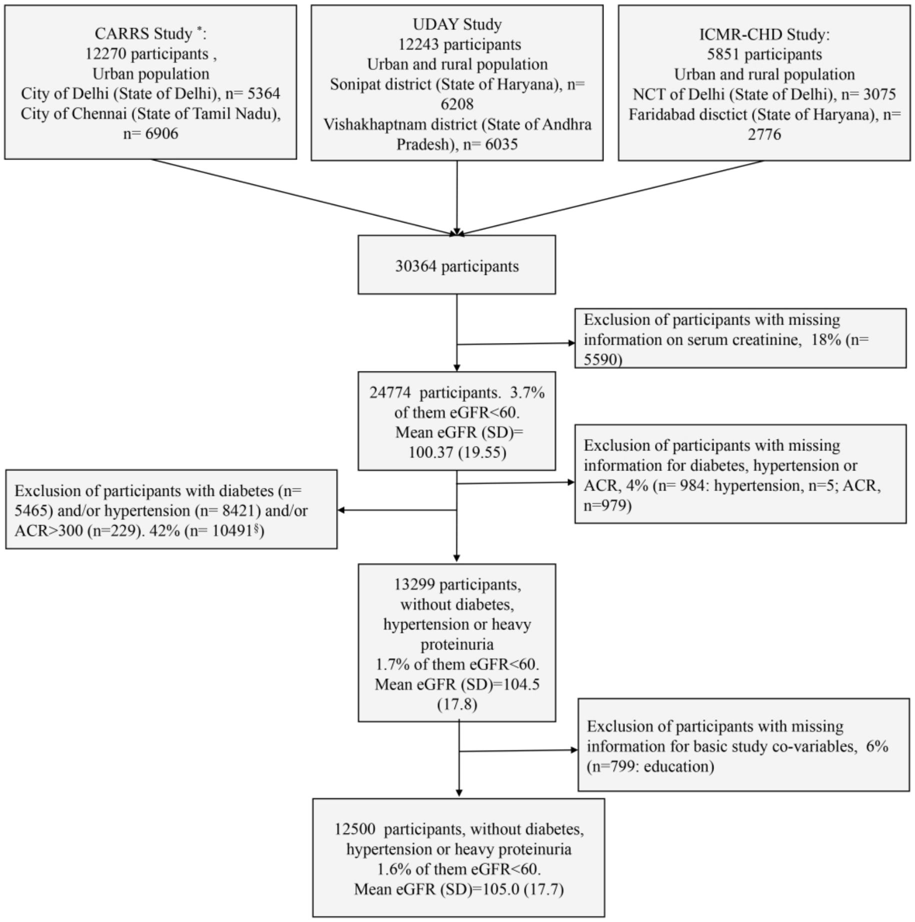

* The original sample size in the CARRS study is 12271, one transgender person was excluded for the current analysis; § 2353 participants with diabetes only; 5185 participants with hypertension only; 35 participants with ACR>30 only; 2724 participants with diabetes, and hypertension; 35 participants with diabetes and ACR>30; 47 participants with hypertension and ACR>30; 112 participants with diabetes, hypertension and ACR>30.

Study flowchart

299x319mm (300 x 300 DPI)

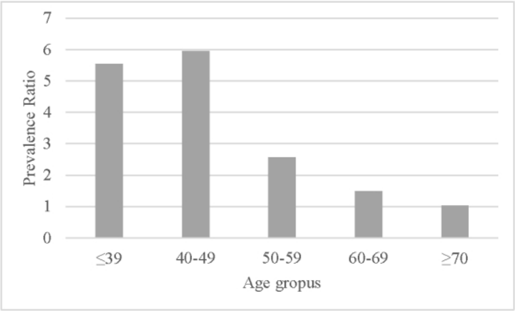

Prevalence ratio of eGFR<60 by age group between rural and urban areas

420x594mm (300 x 300 DPI)

SUPPLEMENTARY MATERIAL

Content

Table S1. Sociodemographic and anthropometric characteristics of overall study participants (prior to exclusion of population with diabetes, hypertension and proteinuria)

Table S2. Associations between sociodemographic and anthropometric characteristics and estimated glomerular filtration rate (eGFR) and eGFR<60 by sex

Table S3. Multiple regression analysis of sociodemographic and anthropometric characteristics associated with eGFR and eGFR<60 including study participants with proteinuria (but without diabetes or hypertension)

Table S4. Multiple regression analysis of sociodemographic and anthropometric characteristics associated with eGFR and eGFR<60 including fasting plasma glucose, HbA1c and systolic blood pressure

For peer review only

Table S1. Sociodemographic and anthropometric characteristics of overall study participants (prior to exclusion of population with diabetes, hypertension and proteinuria)

**Variable**	**n (%)** [a] **n=24774**	**eGFR categories, n(%)** [b]			
		**mean (SD)**	**≥90**	**90 - 60**	**<60**
**Socio-demographic factors**					
Age (years)					
<39	9007 (36)	112.9 (14.9)	8248 (92)	716 (8)	43 (0)
40 - 49	6924 (28)	101.9 (14.8)	5617 (81)	1215 (18)	92 (1)
50 - 59	4524 (18)	92.9 (15.2)	2997 (66)	1378 (30)	149 (3)
60 - 69	3045 (12)	82.8 (17.1)	1410 (46)	1315 (43)	320 (11)
≥70	1274 (5)	72.0 (17.3)	164 (13)	806 (63)	304 (24)
Sex					
Female	13433 (54)	102.6 (19.5)	10404 (77)	2585 (19)	444 (3)
Male	11341 (46)	97.7 (19.3)	8032 (71)	2845 (25)	464 (4)
Education (number of years)					
0	4794 (19)	97.7 (20.2)	3458 (72)	1075 (22)	261 (5)
5	3194 (13)	101.7 (19.4)	2456 (77)	625 (20)	113 (4)
10	8855 (36)	103.2 (18.9)	6995 (79)	1620 (18)	240 (3)
>10	6358 (26)	100.0 (19.2)	4638 (73)	1538 (24)	182 (3)
Missing data	1573 (6)		889 (57)	572 (36)	112 (7)
Area [d]					
Urban	17732 (72)	102 (19.5)	13577 (77)	3602 (20)	553 (3)
Rural	7042 (28)	96.3 (19.1)	4859 (69)	1828 (26)	355 (5)
Latitude [e]					
North	13570 (55)	98.1 (19.1)	9599 (71)	3439 (25)	532 (4)
South	11204 (45)	103.1 (19.7)	8837 (79)	1991 (18)	376 (3)
**Life-style factors**					
Current smoking					
No	18402 (74)	101.5 (19.6)	13920 (76)	3838 (21)	644 (3)
Yes	6372 (26)	97.1 (19.1)	4516 (71)	1592 (25)	264 (4)
Alcohol consumption ever					
No	19588 (79)	100.9 (19.6)	14671 (75)	4203 (21)	714 (4)
Yes	5186 (21)	98.5 (19.1)	3765 (73)	1227 (24)	194 (4)
Vegetarian					
No	15043 (61)	102.7 (19.7)	11721 (78)	2835 (19)	487 (3)
Yes	9731 (39)	96.8 (18.9)	6715 (69)	2595 (27)	421 (4)
**Biological factors**					
Body mass index (kg/m^2)					
Underweight (≤18.5)	10297 (42)	100.1 (19.6)	7626 (74)	2284 (22)	387 (4)
Normal (>18.5 - ≤25)	2403 (10)	101.58 (20.5)	1838 (76)	471 (20)	94 (4)
Overweight (>25 - ≤30)	7221 (29)	99.9 (18.8)	5309 (74)	1680 (23)	232 (3)
Obese (>30)	3286 (13)	99.3 (19.2)	2392 (73)	766 (23)	128 (4)
Missing data	1567 (6)		1271 (81)	229 (15)	67 (4)
Fat free mass (kg)					
1st tertile (≤37)	7141 (29)	101.9 (20.1)	5481 (77)	1381 (19)	279 (4)
2nd tertile (>37 - <45)	7141 (29)	101.3 (19.1)	5419 (76)	1487 (21)	235 (3)

3rd tertile (≥45)	7141 (29)	98.3 (18.6)	5110 (72)	1797 (25)	234 (3)
Missing data	3351 (14)		2426 (72)	765 (23)	160 (5)

[a] Percentages in columns; [b] percentages in rows; [d] Urban areas include Delhi, Chennai and Sonipat district. Rural areas include Sonipat, Vishakhapatnam and Faridabad districts; [e] North areas include Delhi, Sonipat and Faridabad district. South areas include Chennai and Vishakhapatnam districts.

For peer review only

Table S2. Associations between sociodemographic and anthropometric characteristics and estimated glomerular filtration rate (eGFR) and eGFR<60 by sex

	Men, n=5 434			Women, n=7 066		
**Variable**		**eGFR**	**eGFR<60**		**eGFR**	**eGFR<60**
	**n (%)**	**estimate (95%CI) [a]**	**OR (95% CI) [a]**	**n (%)**	**estimate (95%CI) [a]**	**OR (95% CI) [a]**
Age (years) [b]						
<39	2335 (43)	0.00 (ref)	1.00 (ref)	3786 (54)	0.00 (ref)	1.00 (ref)
40-49	1568 (29)	-9 (-9.97, -8.03)	2.36 (1.2, 4.62)	1908 (27)	-12.52 (-13.29, -11.76)	4.5 (1.95, 10.36)
50-59	843 (16)	-16.84 (-18.03, -15.65)	3.82 (1.91, 7.66)	863 (12)	-21.51 (-22.53, -20.48)	11.78 (5.2, 26.68)
60-69	479 (9)	-25.35 (-26.83, -23.86)	13.07 (6.97, 24.49)	414 (6)	-30.05 (-31.46, -28.64)	32.95 (14.87, 73.02)
≥70	209 (4)	-34.26 (-36.4, -32.12)	31.08 (16.33, 59.17)	95 (1)	-34.78 (-37.6, -31.96)	43.43 (15.93, 118.37)
Education (number of completed years)						
0	823 (15)	0.00 (ref)	1.00 (ref)	1997 (28)	0.00 (ref)	1.00 (ref)
≤5	703 (13)	3.28 (1.82, 4.74)	0.24 (0.13, 0.46)	1006 (14)	0.73 (-0.27, 1.73)	0.81 (0.42, 1.56)
6-≤10	2363 (43)	1.68 (0.51, 2.84)	0.31 (0.20, 0.48)	2454 (35)	0.67 (-0.13, 1.48)	0.43 (0.21, 0.86)
>10	1545 (28)	-1.35 (-2.6, -0.1)	0.27 (0.15, 0.47)	1609 (23)	-2.39 (-3.27, -1.5)	0.76 (0.40, 1.46)
Area [c]	3583 (66)					
Urban	1851 (34)	0.00 (ref)	1.00 (ref)	4911 (70)	0.00 (ref)	1.00 (ref)
Rural		-4.02 (-4.85, -3.19)	2.72 (1.84, 4.01)	2155 (30)	-3.69 (-4.36, -3.02)	1.99 (1.26, 3.14)
Latitude [d]						
North	2861 (53)	0.00 (ref)	1.00 (ref)	3402 (48)	0.00 (ref)	1.00 (ref)
South	2573 (47)	-1.52 (-2.3, -0.74)	1.76 (1.21, 2.56)	3664 (52)	2.58 (1.96, 3.19)	1.30 (0.83, 2.05)
Current tobacco consumption						
No	2804 (52)	0.00 (ref)	1.00 (ref)	6553 (93)	0.00 (ref)	1.00 (ref)
Yes	2630 (48)	1.15 (0.36, 1.93)	1.32 (0.91, 1.92)	513 (7)	-1.93 (-3.14, -0.73)	1.54 (0.87, 2.73)
Alcohol consumption ever						
No	3035 (56)	0.00 (ref)	1.00 (ref)	7059 (100)	0.00 (ref)	1.00 (ref)

Yes	2399 (44)	-0.71 (-1.49, 0.06)	1.57 (1.08, 2.27)	7 (0)	-9.29 (-18.97, 0.4)	1.00 (1.00, 1.00)
Vegetarian						
No	3576 (66)	0.00 (ref)	1.00 (ref)	4396 (62)	0.00 (ref)	1.00 (ref)
Yes	1858 (34)	0.65 (-0.18, 1.48)	0.61 (0.41, 0.90)	2670 (38)	-2.11 (-2.75, -1.47)	0.70 (0.44, 1.11)
Body mass index (kg/m2)						
Underweight (≤18.5)	2888 (56)	0.00 (ref)	1.00 (ref)	2991 (44)	0.00 (ref)	1.00 (ref)
Normal (>18.5 - ≤25)	812 (16)	4.05 (2.92, 5.18)	0.69 (0.42, 1.14)	764 (11)	1.61 (0.57, 2.65)	1.07 (0.57, 2.03)
Overweight (>25 - ≤30)	1209 (23)	-1.7 (-2.68, -0.73)	0.71 (0.42, 1.21)	2104 (31)	-0.11 (-0.84, 0.62)	0.67 (0.38, 1.20)
Obese (>30)	243 (5)	-0.71 (-2.61, 1.18)	0.36 (0.09, 1.50)	907 (13)	-0.64 (-1.61, 0.33)	0.55 (0.23, 1.31)
Fat free mass (kg)						
1st tertile (≤37)	361 (8)	0.00 (ref)	1.00 (ref)	3833 (58)	0.00 (ref)	1.00 (ref)
2nd tertile (>37 - <45)	1351 (28)	-0.42 (-2.10, 1.25)	0.78 (0.44, 1.38)	2535 (39)	-1.39 (-2.04, -0.74)	0.67 (0.38, 1.17)
3rd tertile (≥45)	3093 (64)	-3.75 (-5.35, -2.16)	0.50 (0.28, 0.90)	208 (3)	-1.36 (-3.17, 0.45)	0.58 (0.08, 4.25)

[a] Adjusted for age; [b] Not adjusted for age; [c] Urban areas include Delhi, Chennai and Sonipat district. Rural areas include Sonipat, Vishakhapatnam and Faridabad districts; [d] North areas include Delhi, Sonipat and Faridabad district. South areas include Chennai and Vishakhapatnam districts.

view only

Table S3. Multiple regression analysis of sociodemographic characteristics associated with eGFR and eGFR<60 including study participants with proteinuria (but without diabetes or hypertension), n=12533

	**eGFR**	**eGFR<60**
**Variable**	**Coefficient (95%CI) [a]**	**OR (95%CI) [a]**
Area [b]		
Urban	0.00 (ref)	1.00 (ref)
Rural	-4.59 (-5.14, -4.03)	1.93 (1.40, 2.66)
Latitude [c]		
North	0.00 (ref)	1.00 (ref)
South	0.29 (-0.21, 0.78)	1.33 (0.98, 1.80)
Education (number of years)		
0	0.00 (ref)	1.00 (ref)
5	0.83 (0, 1.66)	0.55 (0.35, 0.87)
10	0.04 (-0.64, 0.72)	0.51 (0.35, 0.76)
>10	-3.81 (-4.58, -3.04)	0.66 (0.40, 1.07)
Alcohol consumption ever		
No	0.00 (ref)	1.00 (ref)
Yes	-0.78 (-1.52, -0.05)	1.23 (0.85, 1.79)
Sex		
Female	0.00 (ref)	1.00 (ref)
Male	-2.86 (-3.46, -2.26)	1.38 (0.96, 1.98)
Age (per 10 years)	-9.12 (-9.34, -8.91)	2.23 (2.00, 2.49)

[a] Variables mutually adjusted, [b] Urban areas include Delhi, Chennai and Sonipat district. Rural areas include Sonipat, Vishakhapatnam and Faridabad districts; [c] North areas include Delhi, Sonipat and Faridabad district. South areas include Chennai and Vishakhapatnam districts.

eview only

For peer review only - http://bmjopen.bmj.com/site/about/guidelines.xhtml

Table S4. Multiple regression analysis of sociodemographic characteristics associated with eGFR and eGFR<60 including plasma fasting glucose, HbA1c and systolic blood pressure

	eGFR	eGFR<60
**Variable**	**Coefficient (95%CI)** [a]	**OR (95%CI)** [a]
Area [b]		
Urban	0.00 (ref)	1.00 (ref)
Rural	-4.94 (-5.51, -4.38)	2.29 (1.64, 3.20)
Latitude [c]		
North	0.00 (ref)	1.00 (ref)
South	0.23 (-0.26, 0.72)	1.30 (0.95, 1.77)
Education (number of years)		
0	0.00 (ref)	1.00 (ref)
5	1.03 (0.20, 1.86)	0.49 (0.31, 0.79)
10	0.19 (-0.49, 0.87)	0.47 (0.32, 0.71)
>10	-3.53 (-4.30, -2.76)	0.62 (0.38, 1.02)
Alcohol consumption ever		
No	0.00 (ref)	1.00 (ref)
Yes	-0.72 (-1.46, -0.01)	1.32 (0.90, 1.93)
Sex		
Female	0.00 (ref)	1.00 (ref)
Male	-2.69 (-3.29, -2.09)	1.47 (1.01, 2.12)
Age (per 10 years)	-8.93 (-9.16, -8.70)	2.11 (1.89, 2.38)
Fasting plasma glucose (mg/dl)	-0.06 (-0.08, -0.04)	1.01 (1.00, 1.02)
Hb1Ac (%)	0.03 (-0.56, 0.62)	1.95 (1.34, 2.85)
Systolic blood pressure (mm Hg)	-0.06 (-0.84, -0.04)	1.0 (0.99, 1.02)

[a] Variables mutually adjusted, [b] Urban areas include Delhi, Chennai and Sonipat district. Rural areas include Sonipat, Vishakhapatnam and Faridabad districts; [c] North areas include Delhi, Sonipat and Faridabad district. South areas include Chennai and Vishakhapatnam districts.

For peer review only - http://bmjopen.bmj.com/site/about/guidelines.xhtml

STROBE Statement—Checklist of items that should be included in reports of ***cross-sectional studies***

	Item No	Recommendation	Page/line where the checklist items are located in the paper
**Title and abstract**	1	(*a*) Indicate the study's design with a commonly used term in the title or the abstract	Title and abstract (page 2, lines 29-30)
		(*b*) Provide in the abstract an informative and balanced summary of what was done and what was found	Page 2 , lines 27-45
**Introduction**			
Background/rationale	2	Explain the scientific background and rationale for the investigation being reported	Page 4, lines 68-90
Objectives	3	State specific objectives, including any prespecified hypotheses	Page 4-5, lines 91-95
**Methods**			
Study design	4	Present key elements of study design early in the paper	Page 5, lines 103-111, table 1
Setting	5	Describe the setting, locations, and relevant dates, including periods of recruitment, exposure, follow-up, and data collection	Table 1 and page 6, line 123
Participants	6	(*a*) Give the eligibility criteria, and the sources and methods of selection of participants	Page 5, lines 112-121
Variables	7	Clearly define all outcomes, exposures, predictors, potential confounders, and effect modifiers. Give diagnostic criteria, if applicable	Page 6, lines 149-171
Data sources/ measurement	8*	For each variable of interest, give sources of data and details of methods of assessment (measurement). Describe comparability of assessment methods if there is more than one group	Page 6, lines 123-147
Bias	9	Describe any efforts to address potential sources of bias	Page 6, lines 123-127, 134-138, 145-147
Study size	10	Explain how the study size was arrived at	Figure 1
Quantitative variables	11	Explain how quantitative variables were handled in the analyses. If applicable, describe which groupings were chosen and why	Page 6, lines 123-147
Statistical methods	12	(*a*) Describe all statistical methods, including those used to control for confounding	Page 6, 154-160
		(*b*) Describe any methods used to examine subgroups and interactions	Page 6, line 156; page 6, lines 162-171
		(*c*) Explain how missing data were addressed	Page 6, lines 160-162
		(*d*) If applicable, describe analytical methods taking account of sampling strategy	-
		(*e*) Describe any sensitivity analyses	Page 10, lines 233-235, 139-242
**Results**			
Participants	13*	(a) Report numbers of individuals at each stage of study—eg numbers potentially eligible, examined for	Figure 1

For peer review only

		eligibility, confirmed eligible, included in the study, completing follow-up, and analysed	
		(b) Give reasons for non-participation at each stage	Figure 1
		(c) Consider use of a flow diagram	Figure 1
Descriptive data	14*	(a) Give characteristics of study participants (eg demographic, clinical, social) and information on exposures and potential confounders	Table 2.; page 7-8, lines 177-185
		(b) Indicate number of participants with missing data for each variable of interest	Page 6, lines 160-162
Outcome data	15*	Report numbers of outcome events or summary measures	Page 8, line 187-201
Main results	16	(*a*) Give unadjusted estimates and, if applicable, confounder-adjusted estimates and their precision (eg, 95% confidence interval). Make clear which confounders were adjusted for and why they were included	Page 8, lines 203-220; table 3 and table 4
		(*b*) Report category boundaries when continuous variables were categorized	Table 3 and Table 4
		(*c*) If relevant, consider translating estimates of relative risk into absolute risk for a meaningful time period	-
Other analyses	17	Report other analyses done—eg analyses of subgroups and interactions, and sensitivity analyses	Page 9, lines 221-231; page 10, lines 233-250
**Discussion**			
Key results	18	Summarise key results with reference to study objectives	Page 10, lines 252-263
Limitations	19	Discuss limitations of the study, taking into account sources of potential bias or imprecision. Discuss both direction and magnitude of any potential bias	Pages 12-13, lines 310-322
Interpretation	20	Give a cautious overall interpretation of results considering objectives, limitations, multiplicity of analyses, results from similar studies, and other relevant evidence	Pages 11-12, lines 264-309
Generalisability	21	Discuss the generalisability (external validity) of the study results	Pages 13, lines 323-325
**Other information**			
Funding	22	Give the source of funding and the role of the funders for the present study and, if applicable, for the original study on which the present article is based	Page 3, lines 59-66

*Give information separately for exposed and unexposed groups.

Note: An Explanation and Elaboration article discusses each checklist item and gives methodological background and published examples of transparent reporting. The STROBE checklist is best used in conjunction with this article (freely available on the Web sites of PLoS Medicine at http://www.plosmedicine.org/, Annals of Internal Medicine at http://www.annals.org/, and Epidemiology at http://www.epidem.com/). Information on the STROBE Initiative is available at www.strobe-statement.org.

For peer review only - http://bmjopen.bmj.com/site/about/guidelines.xhtml

For peer review only
